# Assessing Species Delimitation in *Entamoeba* (Amoebozoa: Endamoebidae) Using the Small Subunit rRNA Gene: Its Application to the *Entamoeba polecki* Complex

**DOI:** 10.3390/microorganisms14020360

**Published:** 2026-02-03

**Authors:** Lorena Esteban-Sánchez, Francisco Ponce-Gordo

**Affiliations:** Department of Parasitology, Faculty of Pharmacy, Complutense University, Plaza Ramón y Cajal s/n, 28040 Madrid, Spain; lorees01@ucm.es

**Keywords:** *Entamoeba*, species delimitation criteria, SSU rRNA gene, taxonomy

## Abstract

In this study, we address the criteria for the identification of species and subspecific categories (ribosomal lineages) within the genus *Entamoeba* using an evolutionary species concept and an integrative taxonomic framework, in which genetic evidence may allow species differentiation in the absence of differences in traditional criteria such as morphology or host specificity. Rather than relying on distance-based phylogenetic analyses, we apply quantitative criteria based on genetic divergence and on the presence of structural modifications (compensatory base changes) in the secondary structure of the SSU rRNA molecule. This approach is more conservative than Birky’s 4× rule and may fail to discriminate recently diverged lineages; however, when its criteria are fulfilled, they provide high-assurance evidence of species-level divergence. To apply these criteria, we assembled a curated set of reference SSU rDNA sequences for those *Entamoeba* species identified at the species level (bearing valid specific epithets) for which sequence data are currently available, providing a standardized framework for the accurate assignment of published sequences. We determined their SSU rRNA secondary structures using previously published experimental data and in silico analyses. Using this framework, we show that *Entamoeba polecki*, *Entamoeba struthionis*, and *Entamoeba chattoni*, previously regarded as synonyms, represent distinct species.

## 1. Introduction

Describing, delimiting, and naming species is usually referred to as alpha taxonomy. Although these terms are often used interchangeably, species identification and species delimitation represent conceptually distinct processes in taxonomy [1]. Identification refers to the assignment of an individual organism or sample to a previously described species, typically based on diagnostic features such as morphology, genetic markers, or ecological traits, and is grounded in comparison with existing taxonomic frameworks. In contrast, delimitation involves determining whether a group of organisms constitutes a distinct species in the first place. This process requires the evaluation of multiple characteristics of the population under study, for example, morphological traits, genetic divergence, reproductive isolation, phylogenetic distinctiveness, or ecological separation from other populations. Species delimitation should be based on a consistent set of criteria that are aligned with a defined species concept and is therefore a more fundamental and interpretative task, often preceding formal description and naming [2]. It is particularly critical in groups in which cryptic diversity or limited morphological differentiation complicates identification, as occurs in many protozoa [3,4].

Species delimitation in the protozoan genus *Entamoeba* (Amoebozoa: Endamoebidae) has long been, and remains, a matter of debate. The family name Endamoebidae is used here because Entamoebidae Chatton, 1925 was proposed without a properly fixed type genus, whereas Endamoebidae Calkins, 1926 was validly established in accordance with the nomenclatural rules in force both at the time and today [5]. This genus comprises predominantly endobiont amoebae inhabiting the digestive tract of vertebrates, ranging from fishes to mammals and birds [6,7]; a few species are free-living or may exhibit a dual free-living/endobiont lifestyle. Most *Entamoeba* species are considered commensals, although some display pathogenic potential [6]. In endobiont species, the trophozoite represents the active stage, whereas the cyst constitutes the dormant transmission stage to a new host. The only confirmed exception to date is *Entamoeba gingivalis*, which inhabits the human buccal cavity and does not form cysts [8].

Only a limited number of morphological traits are available for species delimitation in *Entamoeba*, mainly trophozoite and cyst size, as well as the morphology and number of nuclei in cysts. Additional criteria, such as pathogenicity, geographic origin, and host species, were also considered key taxonomic characters in studies conducted during the 20th century [6]. However, these supplementary criteria are not always reliable and have often proven inconsistent for species delimitation and identification. This has led to the synonymization of several described species [9,10], while many others remain in an uncertain taxonomic status owing to the lack of subsequent comparative studies since their original description [6].

With the development of molecular analytical methods, the small subunit rRNA (SSU rRNA) gene has become the genetic marker most commonly used in taxonomic and epidemiological studies of *Entamoeba*. This marker evolves relatively rapidly in this genus, providing sufficient resolution to differentiate taxa using this gene alone [11]. The incorporation of genetic data has confirmed the validity of several species that had previously been regarded as synonyms [12,13,14] and has supported the description of new species [15,16,17,18,19]. Nevertheless, no standardized criteria have been established to assess genetic divergence for species delimitation in *Entamoeba*. Depending on the authors’ approaches, genetic differences may be subordinated to less robust criteria, such as host range.

Increasing sampling and molecular characterization of *Entamoeba* isolates from both previously known and newly identified host species have revealed extensive genetic diversity in the SSU rRNA gene and have broadened the recognized host range of several species [20,21,22]. However, this has not been accompanied by a corresponding increase in the number of formally described or confirmed species, largely because morphological data were unavailable, as explicitly noted by the respective authors. A non-standard nomenclature was therefore proposed to group isolates according to their placement in phylogenetic trees and their affinity with previously described species [20,21]. Nonetheless, key concepts such as “well-supported phylogenetic clusters” and “strong affinity with previously described species” were only loosely defined [20] (p. 531), potentially leading to inconsistencies in species delimitation. Since then, no further comprehensive proposals or revisions of species delimitation criteria in *Entamoeba* have been put forward.

The present study aims to evaluate objective and reproducible criteria for species delimitation in *Entamoeba* based on the SSU rRNA gene, by integrating measures of genetic variability with structural features of the SSU rRNA molecule. This approach is intended to provide a framework applicable to future taxonomic, biodiversity, and epidemiological studies. The validity and usefulness of these criteria are assessed by applying them to two sets of species. The first includes the tetranucleate cyst-forming species *E. histolytica*, *E. nuttalli*, *E. dispar* (which display high genetic similarity yet are widely accepted as distinct species [20]), *E. bangladeshi* and *E. moshkovskii*. The second set comprises the uninucleate cyst-forming species *E. polecki*, *E. chattoni*, and *E. struthionis*, which, despite showing lower genetic similarity, have been regarded as synonyms because they are phylogenetically related and share host ranges [13]. Within each group, species are morphologically indistinguishable. While *E. histolytica*, *E. nuttalli*, and *E. moshkovskii* are pathogenic or potentially pathogenic, the remaining species are generally considered non-pathogenic [6,17,23].

## 2. Materials and Methods

### 2.1. Sequence Retrieval

All SSU rRNA gene (SSU rDNA) sequences longer than 400 bp and identified by the original authors to the species level within the genus *Entamoeba* (i.e., excluding records annotated as *Entamoeba* sp.) were retrieved from the GenBank database (https://www.ncbi.nlm.nih.gov/genbank/; accessed on 30 January 2025) as of 30 December 2024. Search queries included the term “*Entamoeba*” in combination with “SSU,” “small subunit,” “18S,” or “16S.” When sequences extended into the internal transcribed spacer (ITS) region, this portion was trimmed prior to analysis.

### 2.2. Verification of Species Assignment

Previous work has identified several *Entamoeba* sequences deposited in GenBank with incorrect species-level assignments [22]. To minimize the impact of misannotations, all sequences assigned to a given species were verified by comparison with reference sequences, defined as those first published for each species either in the original species description or in subsequent authoritative studies (Table 1), and were reannotated when necessary.

For *E. polecki*, two grouping schemes were applied. In the first scheme, *E. polecki* sensu Prowazek, 1912 (hereafter *E. polecki* sensu stricto, s.s.) was treated as distinct from *E. chattoni* and *E. struthionis*, and sequences were compared with their respective reference sequences. After correct assignment, all sequences were subsequently grouped as *E. polecki* following the synonymy proposed by Clark et al. (2006) [13] (hereafter *E. polecki* sensu lato, s.l.).

For verification of species assignment, separate alignments were generated for sequences annotated in GenBank as belonging to the same species, using MUSCLE [30] as implemented in MEGA X [31] with default parameters. These alignments were preliminary and were subjected only to minimal manual editing to ensure the correct alignment of conserved regions; variable regions were not refined at this stage. Sequences displaying nucleotide differences clustered within a short region (defined here as at least two differences within a five-position sliding window) were flagged for further evaluation and were subsequently compared with the reference sequences (Table 1) using the BLASTN algorithm (available at the NCBI website https://blast.ncbi.nlm.nih.gov/Blast.cgi) and were reassigned when necessary. Divergent fragments restricted to the 3′ or 5′ ends were considered likely PCR or sequencing artifacts and were trimmed. When a discordant, unalignable fragment was detected in the alignment, the corresponding region was manually extracted and analyzed using the BLASTn algorithm to assess its possible origin. Only species represented by at least five sequences after assignment verification were retained for subsequent analyses.

### 2.3. Secondary Structure Determination and Sequence Alignment Refinement

The secondary (2D) structure of the SSU rRNA of *E. histolytica* was derived from its ribosomal three-dimensional (3D) model (Protein Data Bank entry 9V24) using DSSR version 2.5.0-2025 [32]. Because the experimental 3D model lacked atomic coordinates for some regions, the resulting 2D structure was incomplete, and the helix configurations proposed for these regions were tentative [33]. Three-dimensional models of the missing regions were therefore generated in silico using AlphaFold3 (https://alphafoldserver.com/welcome; accessed on 3 February 2025) [34], and the corresponding 2D structures were extracted with DSSR. In addition, independent 2D structure predictions were generated using the RNAfold utility from the ViennaRNA Package v2.6.3 [35], as implemented in the RNAfold WebServer (http://rna.tbi.univie.ac.at/cgi-bin/RNAWebSuite/RNAfold.cgi; last accessed on 25 June 2025), with default parameters.

The *E. histolytica* SSU rRNA secondary-structure models obtained from AlphaFold3 and RNAfold were edited and compared with the published proposal [33] using 4SALE [36]. Discrepancies among the models were detected only in a region involving positions 203–251 of the *E. histolytica* sequence: a single helix involving this fragment was proposed in the incomplete experimental model, whereas two helices were predicted by both the in silico 2D and 3D models. The most plausible configuration was evaluated through comparative analysis of homologous SSU rRNA sequences from other *Entamoeba* species and by covariation testing (Supplementary File S1). Based on these analyses, a two-helix conformation was selected for this region and adopted in the final 2D structure model.

The complete secondary-structure model was subsequently propagated to the reference sequences of the remaining *Entamoeba* species using the “model” option available in the ITS2 Database website (https://its2.bioapps.biozentrum.uni-wuerzburg.de/; accessed on 8 July 2025) [37]. A combined sequence-structure alignment was then generated in 4SALE. For sequences in which certain regions showed partial or no helix transfer, or where structural inconsistencies across species were detected (e.g., absence of helices or homologous fragments involved in different structural elements), the affected regions were re-modelled using the RNAfold WebServer and manually corrected in 4SALE. For graphical representation, secondary-structure diagrams were generated using RNAviz 2.0 [38] (Supplementary File S2).

### 2.4. Intraspecific Genetic Variability: Nucleotide Diversity and Distance Analyses

To assess within-species genetic variability, nucleotide diversity (π) [39] was estimated for all *Entamoeba* species included in Table 1 for which five or more sequences were available. The π parameter, defined as the average number of nucleotide differences per site between two randomly chosen sequences, provides a summary measure of intraspecific genetic variation. Pairwise genetic distances were calculated in MEGA X using the Tamura–Nei substitution model with a gamma distribution (G = 0.1), which was selected based on both the Akaike Information Criterion (AIC) and the Bayesian Information Criterion (BIC). Statistical confidence was assessed using 1000 bootstrap replicates.

To minimize potential bias arising from short sequence overlaps, an overlap-filtered dataset was generated by processing the distance matrix with custom Python scripts (Python v3.13.5 [40]; Biopython v1.85 [41]), in which all pairwise comparisons involving sequences overlapping fewer than 100 nucleotide positions were excluded. Because valid distances were not available for all sequence pairs after filtering, nucleotide diversity (π) was calculated following Nei and Li (1979) [39] as the arithmetic mean of all valid pairwise distances. Basic descriptive statistics, including mean, standard deviation, and minimum and maximum values, were computed in R version 4.3.3 [42].

To determine a threshold separating intra- from interspecific genetic variability, the *E. histolytica*-like clade (*E. histolytica*, *E. nuttalli*, and *E. dispar*) was used as a reference group. These species are phylogenetically closely related, represent a recently diverged monophyletic lineage [43], and are well-established taxonomic entities [20,21]. Sequence data from the phylogenetically related species *E. bangladeshi*, for which only a limited number of sequences are available, and *E. moshkovskii*, which is considered a species complex [23], were also included in these analyses.

To evaluate potential biases associated with incomplete sequence coverage, additional Python scripts were used to generate a length-filtered dataset of pairwise genetic distances, retaining only sequences covering at least 75% of the alignment (approximately 1500 bp). Nucleotide diversity (π) was recalculated using the filtered distances. In both the overlap-filtered and length-filtered datasets, combined-species alignments were constructed by starting with *E. histolytica* and progressively adding other species according to their phylogenetic proximity (i.e., *E. histolytica* + *E. nuttalli*; *E. histolytica* + *E. nuttalli* + *E. dispar*; and so forth). Nucleotide diversity (π) was recalculated for each cumulative grouping, generating an ordered series of π values corresponding to increasing phylogenetic breadth.

To further characterize shifts in the distribution of pairwise genetic distances across species combinations, non-parametric statistical analyses were performed in R using the tidyverse [44], rstatix [45], and clinfun [46] packages. For each alignment (single-species and combined), summary statistics (mean and standard deviation) were computed, and differences in pairwise genetic distances among combinations were evaluated using Kruskal–Wallis tests, followed by pairwise Wilcoxon rank-sum tests with Holm correction. The presence of a monotonic trend in genetic distance across successive combinations was assessed using the Jonckheere–Terpstra test, with significance evaluated by Monte Carlo resampling (5000 permutations) for large datasets. Bootstrap 95% confidence intervals for the mean were estimated from 1000 resampled datasets per alignment. All statistical analyses and visualizations were conducted in RStudio version 2025.09 (Posit Software PBC, Boston, MA, USA).

To identify the point at which nucleotide diversity increased beyond the level compatible with intraspecific variability, a change-point analysis was applied based on the minimization of the within-segment sum of squared errors (SSE). To assess whether the observed improvement in model fit (ΔSSE) exceeded that expected by chance, a permutation test with 10,000 replicates was implemented in R, under the null hypothesis that the observed series of π values exhibited no structured ordering. Because *E. moshkovskii* may represent a species complex, its data were excluded from this calibration step but were subsequently used for a posteriori comparison. The π value at the inferred change point (π*) was retained as an empirical threshold distinguishing intraspecific variability from the increased variability observed when multiple species are combined.

Using the π* value derived from the *E. histolytica*-like clade, its applicability was then evaluated for *E. moshkovskii* and for the species included within *E. polecki* s.l. For the latter group, nucleotide diversity was calculated for each species individually and for cumulative species combinations (*E. polecki*–*E. struthionis*; *E. polecki*–*E. struthionis*–*E. chattoni*), using two datasets defined by the filtering criteria applied (sequence overlap and ≥75% alignment coverage).

In addition to the π-based threshold analysis, the “4× rule” proposed by Birky and colleagues [47] was also applied. This rule states that the sequence divergence between individuals belonging to different clades (e.g., species or ribosomal lineages) should be at least four times greater than the mean pairwise divergence observed within each clade. Although originally formulated for assigning individuals to species rather than for direct species-level comparisons, it was used here as a complementary, ratio-based criterion to evaluate species boundaries within the *E. histolytica*-like clade (including *E. bangladeshi* and *E. moshkovskii*) and among the species included in *E. polecki* s.l. Between-species pairwise genetic distances used in this analysis were extracted from the previously generated distance matrices by retaining only values corresponding to interspecific comparisons (species A–species B). These datasets were then subjected to the same overlap- and length-based filtering procedures described above. For each species pair (A, B) in the *E. histolytica*-like clade and in *E. polecki* s.l., the mean interspecific genetic distance (K_AB_) was calculated from all pairwise comparisons between sequences of species A and B. Following Birky et al. (2005) [47], θ_AB_ was defined as the higher of the two within-species nucleotide diversity values (θ_AB_ = max[π_A_, π_B_]), and the ratio R_AB_ = K_AB_/θ_AB_ was computed. Species pairs were considered to satisfy the 4× rule when R_AB_ ≥ 4. For each species pair, 95% bootstrap confidence intervals for within- and between-species mean genetic distances, as well as for the R = K/θ ratio, were estimated by resampling distance values with replacement (1000 bootstrap replicates). All analyses were conducted in RStudio.

### 2.5. Species Delimitation by Computational Analyses and Structural Characteristics of the SSU rRNA

Additional analyses were performed to evaluate the performance of computational species-partitioning tools and to assess features of the SSU rRNA secondary structure that may be informative for species delimitation. Their performance was evaluated using the three species of the *E. histolytica*-like clade and was also applied to the species included in *E. polecki* s.l. Analyses were conducted on the complete SSU rRNA gene as well as separately on each of its four major structural domains (5′ major, central, 3′ major, and 3′ minor domains; [48]) to examine whether sequence variability and interspecific differences are uniformly distributed across the gene.

To ensure reliable and meaningful comparisons, both for the complete gene and for individual domains, only sequences covering at least 75% of the corresponding alignment were retained for comparative analyses. This filtering criterion was applied to minimize the impact of fragmentary data and to ensure the robustness of diversity estimates and structural comparisons. Under these conditions, any pair of sequences shared at least 50% overlap, and sequences covering only non-overlapping regions were excluded from the analyses.

#### 2.5.1. Population Structure Analysis

To evaluate the degree of genetic differentiation among groups, an Analysis of Molecular Variance (AMOVA) was performed in Arlequin v3.5 [49] for each species group, starting with the most closely related taxa within each set (*E. histolytica* and *E. nuttalli* in one case, *E. polecki* and *E. struthionis* in the other). AMOVA partitions the total genetic variance into components attributable to differences within and among predefined groups. The significance of variance components was assessed using 10,000 permutations. The resulting Φ_ST_ statistic, representing the proportion of total genetic variance explained by differences among species, was used to evaluate whether the proposed lineages correspond to genetically structured and differentiated populations. High and statistically significant Φ_ST_ values support the interpretation that the lineages represent evolutionarily independent entities.

In addition, species delimitation was assessed using multiple distance-based partitioning methods implemented in ASAP (Assemble Species by Automatic Partitioning; [50]), using the web interface (https://bioinfo.mnhn.fr/abi/public/asap, accessed on 4 July 2025, The web-based version of the program was hosted by the Muséum National d’Histoire Naturelle (Paris) and was operational during the study, but the server is currently offline. The local version can be downloaded at https://www.itaxotools.org/download.html (accessed on 29 January 2026), although it has less interactive features). Analyses were performed under the Jukes–Cantor substitution model with default parameters. ASAP analyses were conducted separately within the *E. histolytica*-like clade and within *E. polecki* s.l., in order to avoid mixing distinct evolutionary scales, which could result in partitions driven by deep phylogenetic divergences rather than providing informative fine-scale species partitioning.

#### 2.5.2. Mapping of Variable Positions on RNA Structure, Identification of Compensatory Base Changes, and Barcode Search

For each species within each group (the *E. histolytica*-like clade and *E. polecki* s.l.), a consensus sequence was generated based on the majority rule at each alignment position. To facilitate accurate positional mapping of sequence variation, these consensus sequences retained the full alignment length; insertion–deletion (indel) positions were preserved even when a nucleotide was present in only a single sequence. The SSU rRNA secondary structures for each species were modelled as described in Section 2.3. Variable positions among sequences within each species were subsequently mapped onto the corresponding secondary-structure diagrams, allowing visualization of the distribution and clustering of mutations across structural elements.

To map sequence differences among species, a separate consensus sequence was generated for each species using the majority rule at each position and retaining only alignment positions with at least 50% sequence coverage to ensure reliable interspecific comparisons. For positions at which two alternative nucleotides were present and the minor variant occurred in more than 25% of the sequences, the corresponding IUPAC ambiguity code was assigned. These interspecific consensus sequences were aligned, and variable positions were mapped onto the 2D secondary-structure models to identify sequence fragments and structural motifs potentially informative for species delimitation.

The presence of compensatory base changes (CBC; defined as paired substitutions at both nucleotides of a base-paired position that preserve the secondary structure of the rRNA molecule) was investigated using 4SALE. CBCs and related structural changes were recorded and evaluated as indicators of evolutionary divergence among lineages.

## 3. Results

### 3.1. Sequence Identification

A total of 1844 SSU rDNA sequences were downloaded. Of these, 98 sequences (5.31% of the total) were excluded for various reasons. These included sequences corresponding to the host organism rather than to *Entamoeba* (e.g., the sequence *E. histolytica* ON086988, from a human sample, showed 99.5% identity to the human ribosomal sequence MF164261), as well as sequences with doubtful taxonomic assignment. Examples of the latter include sequences MN749976 and MN749979, both derived from horse isolates, which are identical to each other but were assigned to two different species (*E. moshkovskii* and *E. ecuadoriensis*). Due to their low similarity (approximately 87%) to the reference sequences of these species, or to any other species within the genus, a reliable assignment was not possible and were discarded.

Among the remaining 1746 accepted sequences, chimeric artifacts were detected in some cases and were trimmed accordingly. These artifacts consisted of sequence repeats, including direct repeats (e.g., sequence MK142736, positions 1–48 and 540–584) and inverted repeats or reverse-complement palindromes (e.g., sequence MK142735, positions 5–199).

Overall, 215 sequences (12.32%) were reassigned to a different species. Of these, 88 sequences corresponded to *E. chattoni* (51) and *E. struthionis* (37), which had originally been identified as *E. polecki* s.l. One sequence initially identified as *E. hartmanni* was reassigned to *E. chattoni*. The remaining 126 reassigned sequences had been originally annotated as *E. histolytica* but were shown to correspond instead to *E. nuttalli* (113 sequences), *E. dispar* (10 sequences), and *E. moshkovskii* (3 sequences) (Table 2).

### 3.2. Nucleotide Diversity and Distance Analysis

Intraspecific nucleotide diversity (π) ranged from 0.00123 in *E. struthionis* to 0.15670 in *E. coli* (Table 2). In the analysis of the *E. histolytica*-like clade, nucleotide diversity increased progressively as sequences from additional species were incorporated into cumulative alignments. In both datasets filtered by sequence overlap and by sequence length, individual species exhibited low intraspecific π values, which increased as additional species were included in the analyses. The length-filtered dataset yielded substantially lower π values for individual species (except for *E. moshkovskii*) but similar or higher values when species were combined (Table 3). Trend analyses confirmed a significant monotonic increase in pairwise genetic distances across successive species combinations in both datasets (Jonckheere–Terpstra test, *p* < 0.001), consistent with increasing phylogenetic breadth (Table 4 and Table 5).

A global comparison of the empirical distance distributions (Table 4 and Table 5) revealed highly significant differences among all single-species and mixed-species combinations (Kruskal–Wallis test, χ^2^_overlap-filtered dataset_ = 62,633, χ^2^_length-filtered dataset_ = 2061, df = 8, *p* < 0.001 for both datasets). Pairwise Wilcoxon rank-sum tests further showed that all comparisons of nucleotide diversity in single species vs. mixed-species alignments were highly significant after Holm correction (*p*.adj < 0.001), indicating that the distributions of pairwise distances within single-species are consistently narrower and shifted towards lower values comoared with those obtained from mixed-species alignments. Comparisons among mixed-species combinations were also consistently significant but provided limited additional information beyond the monotonic trend already demonstrated by the Jonckheere–Terpstra test.

When species were progressively combined in the alignments, the SSE-based change-point analysis (Table 6) consistently placed the optimal breakpoint, in both the overlap-filtered and the length-filtered datasets, between the two-species combination (*E. histolytica* + *E. nuttalli*) and the three-species combination (*E. histolytica* + *E. nuttalli* + *E. dispar*). This result supports the use of π values in the range of 0.010–0.012 as a conservative threshold separating within-species variability from variability observed in mixed-lineages alignments. Notably, the π values estimated for *E. moshkovskii* and for cumulative species combinations within the *E. polecki* group fall within, or clearly exceed, this range (Table 3). However, permutation tests did not provide statistical support for the inferred breakpoint, as the observed reduction in SSE was not significantly greater than expected by chance.

When species boundaries within the *E. histolytica*-like clade and the *E. polecki* s.l. group were evaluated using the 4× rule (Table 7 and Table 8), all species pairs exceeded the 4× threshold except *E. histolytica*–*E. nuttalli* and, marginally, *E. histolytica*–*E. dispar* when the overlap-filtered distance dataset was used. In both cases, the K/θ ratio fell below or close to the 4× criterion. These results coincided with marked differences in within-species nucleotide diversity depending on the dataset applied (Table 3), which may have inflated θ values in the *E. histolytica* comparisons and, consequently, reduced the corresponding K/θ ratios.

### 3.3. Species Delimitation—Population Structure

In the initial AMOVA, the most closely related species within each group were compared (*E. histolytica*–*E. nuttalli*, and *E. polecki* s.s.–*E. struthionis*) (Table 9). In both cases, the proportion of genetic variation attributable to differences between species exceeded the variation observed within species, resulting in very high Φ_ST_ values (>0.95). Because these pairwise comparisons already indicated strong genetic structuring consistent with species-level differentiation, more distantly related species within each group (i.e., *E. dispar* and *E. chattoni*, respectively) were not included in subsequent AMOVA analyses.

AMOVA conducted for both species pairs across the structural domains of the SSU rRNA molecule (Table 10) showed that interspecific variance was consistently greater than intraspecific variance and statistically significant in all regions for both species groups, with the exception of Region IV in the *E. histolytica*–*E. nuttalli* comparison, where no detectable differentiation was observed. The magnitude of the Φ_ST_ values indicated moderate to high genetic differentiation between *E. histolytica* and *E. nuttalli*, and extremely high differentiation between *E. polecki* s.s. and *E. struthionis*.

**Figure 1:**
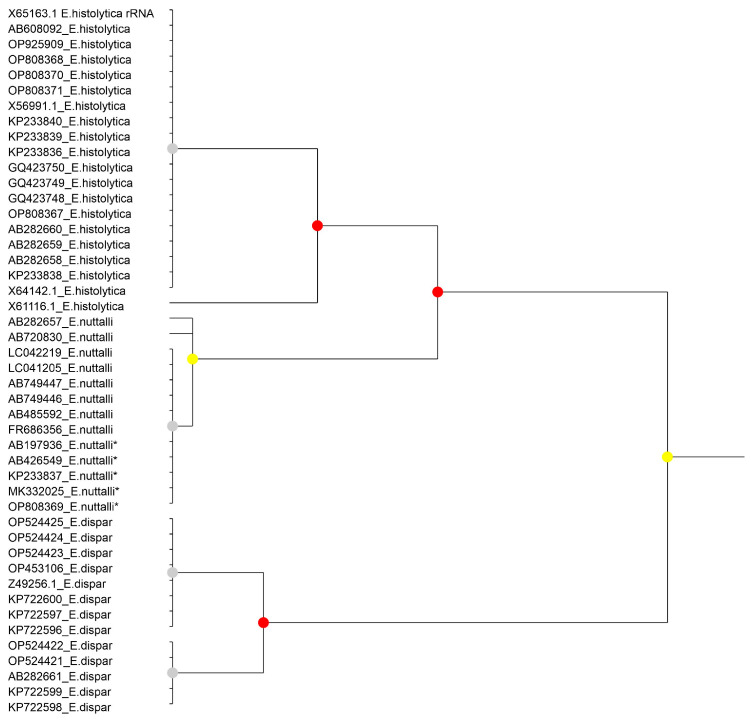
Results of the partitioning analysis of *Entamoeba histolytica*-*Entamoeba nuttalli*-*Entamoeba dispar* in ASAP using sequences covering >75% of the SSU-rRNA molecule. Sequences marked with asterisk (*) were identified in Genbank as belonging to another species but were reannotated in this study. Color dots at the nodes indicate group probability: red, <0.05; yellow: >0.1; grey, not applicable.

**Figure 2:**
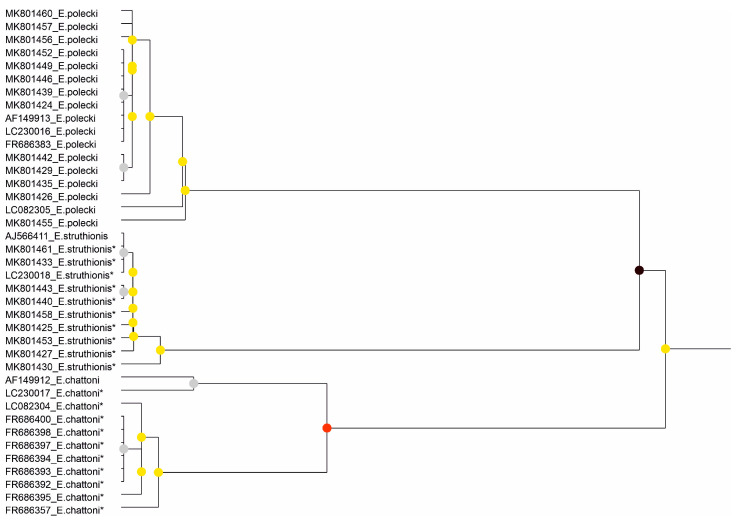
Results of the partitioning analysis of *Entamoeba polecki*-*Entamoeba chattoni*-*Entamoeba struthionis* in ASAP using sequences covering >75% of the SSU-rRNA molecule. Sequences marked with asterisk (*) were identified in Genbank as belonging to another species but were reannotated in this study. Color dots at the nodes indicate group probability: black, <0.001; red, <0.05; yellow: >0.1; grey, not applicable.

The results of species partitioning using ASAP varied depending on whether the complete SSU rDNA sequence or individual structural domains were analysed. When the complete sequence was used for the *E. histolytica*–*E. nuttalli*–*E. dispar* group, the three species were recovered as distinct partitions (groups with *p* < 0.05; Figure 1). However, domain-based analyses (Supplementary File S3) showed no differentiation in Region IV. In the remaining three domains, *E. dispar* consistently formed a separate group, whereas *E. histolytica* and *E. nuttalli* did not form homogeneous and clearly separated clusters. In contrast, for the species included in the *E. polecki* s.l. group, all three species were clearly differentiated both in analyses based on the complete SSU rDNA sequence (*p* < 0.05 or *p* < 0.001; Figure 2) and in analyses of the individual structural domains (Supplementary File S4).

### 3.4. Mapping of Variable Positions on RNA Structure

At the intraspecific level, sequence variation in the SSU rDNA of species belonging to the *E. histolytica*-like clade and to *E. polecki* s.l. was distributed across the entire gene. When all alignment positions showing any nucleotide difference in at least one sequence were considered, the number of variable positions was very high. However, when only positions at which the same variation was present in at least two sequences were taken into account, the number of variable positions decreased markedly (Supplementary File S5), both for the *E. histolytica*-like dataset and for the *E. polecki* s.l. group.

**Figure 3:**
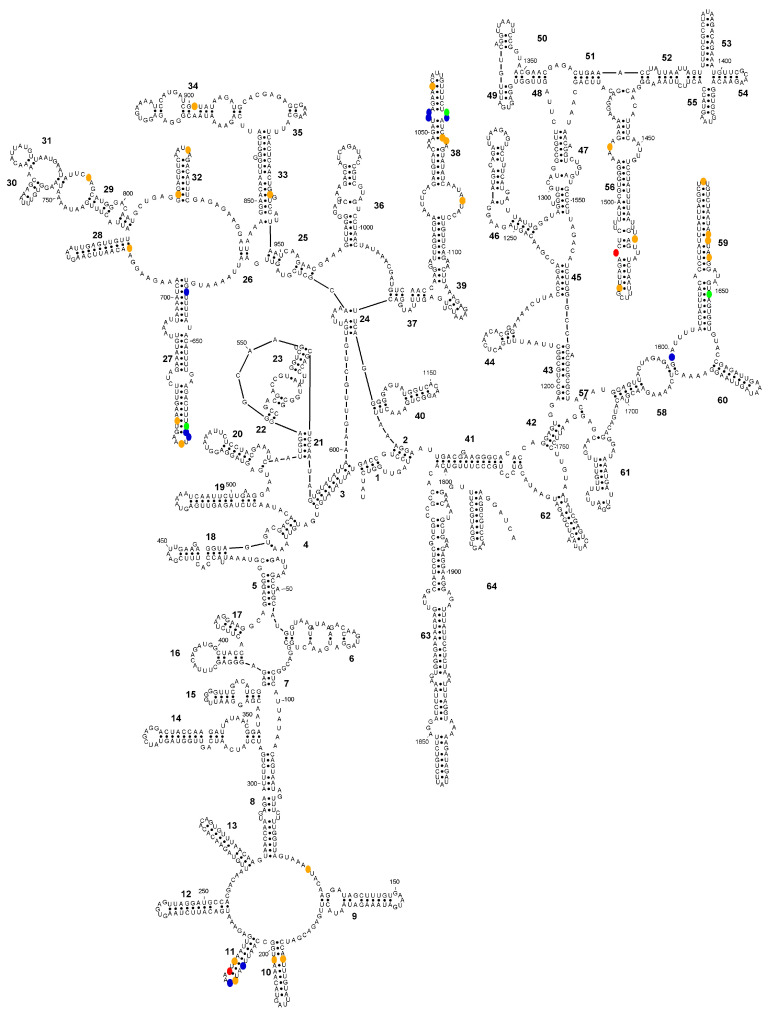
Secondary structure diagram of the SSU-rRNA of *Entamoeba histolytica* (consensus sequence) showing the positions (in colour) where the consensus sequences of *E. histolytica*, *Entamoeba nuttalli* and *Entamoeba dispar* differ. Colour codes: blue, *E. histolytica* is different; green, *E. nuttalli* is different; orange, *E. dispar* is different; red, all sequences differ.

**Figure 4:**
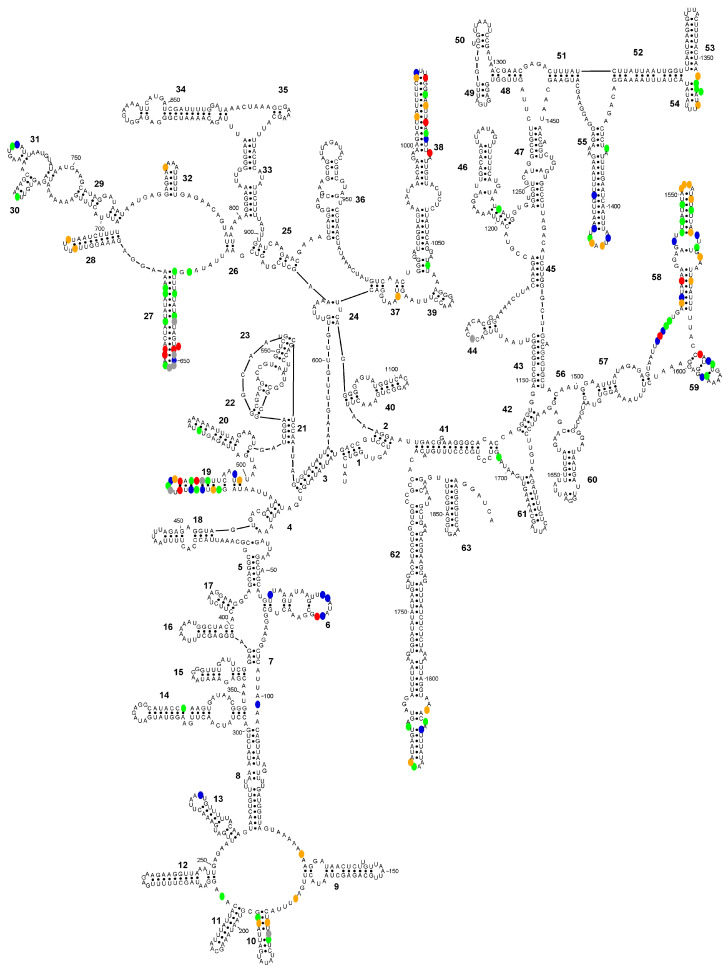
Secondary structure diagram of the SSU-rRNA of *Entamoeba polecki* sensu stricto (consensus sequence) showing the positions (in colour) where the consensus sequences of *E. polecki* s.s., *E. chattoni* and *E. struthionis* differ. Colour codes: blue, *E. polecki* s.s is different; green, *E. chattoni* is different; orange, *E. struthionis* is different; red, all sequences differ; grey, ambiguities present in *E. chattoni* sequence.

In interspecific comparisons based on consensus sequences, the number of differences was substantially higher among species within the *E. polecki* s.l. group than among those in the reduced *E. histolytica*-like group (Figure 3 and Figure 4). Within the latter group, most interspecific differences corresponded to variations in *E. dispar* relative to the other two species (*E. histolytica* and *E. nuttalli*) (Figure 3).

### 3.5. Species Delimitation—CBC Analysis and Barcode Search

**Figure 5:**
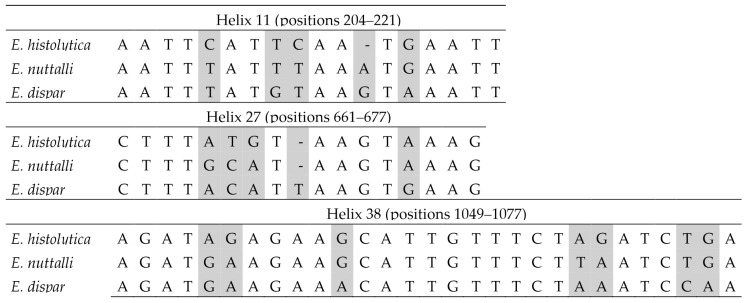
Barcodes in the SSU-rDNA sequence for the differentiation between *Entamoeba histolytica*, *Entamoeba nuttalli* and *Entamoeba dispar*. Base positions and helix numbers are according to the secondary structure in Figure 3. Differential positions are shadowed.

**Figure 6:**
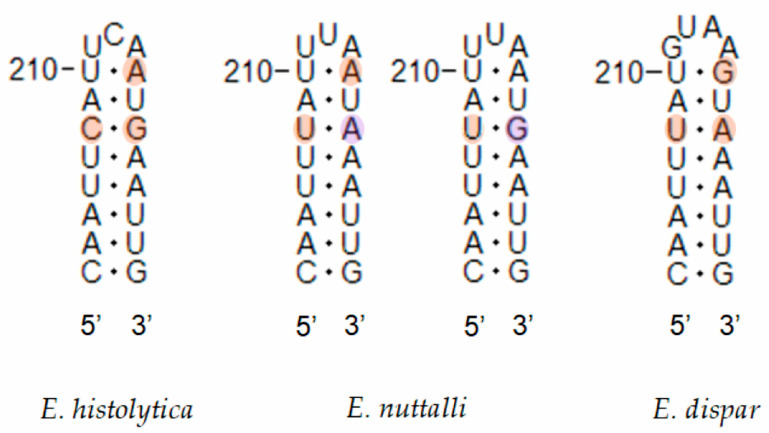
Secondary structure diagrams of the SSU rRNA helices showing compensatory base changes between *Entamoeba histolytica*, *Entamoeba nuttalli* and *Entamoeba dispar*. Base positions and helix numbers are according to the secondary structure in Figure 3.

Within the *E. histolytica*-like clade, the three species can be distinguished using sequence barcodes located in helices 11, 27 and 38 (Figure 5). No CBCs were detected among sequences belonging to the same species. In interspecific comparisons, a single CBC was identified in helix 11 that distinguishes *E. dispar* from the other two species (Figure 6). In this helix, an A/G ambiguity occurs at position 216 in the *E. nuttalli* consensus sequence, which may generate alternative structural configurations. Of the 14 available *E. nuttalli* sequences that include this position, two contain A216, forming a CBC relative to *E. histolytica* (U208–A216 versus C208–G216). However, this base pair in those two *E. nuttalli* sequences is identical to that observed in *E. dispar* (U208–A217), meaning that these sequences do not form any CBC with respect to *E. dispar*. In the remaining 12 *E. nuttalli* sequences, position 216 is occupied by G, which generates a hemi-CBC (i.e., a substitution affecting only one nucleotide of the paired position). This configuration differs from both *E. histolytica* (U208–G216 versus C208–G216) and *E. dispar* (U208–G216 versus U208–A217) (Figure 6).

In the case of species included within *E. polecki* s.l., the number of positions at which the three species differ is considerably higher. No intra-specific CBCs were detected in *E. polecki* or *E. struthionis*. In *E. chattoni*, one sequence (PP064054) showed a relatively low similarity to the reference sequence AF149912 (95.05%; 519/546 identical positions); all 27 substitutions were concentrated within a 109-bp fragment located approximately 60 bp upstream of the 3′ end. This fragment spanned helices 16 to 19 in the secondary structure, disrupting 50% (3 out of 6) or the pairs in helix 16, 75% (3/4) in helix 17, 50% (2/4) in base of helix 18 and 18% (2/11) in helix 19, and generating a CBC in helix 19 relative to the reference sequence (Supplementary File S6). The remaining *E. chattoni* sequences could be divided into two groups based on visual inspection of the alignment; however, no CBCs were observed between their secondary structures.

**Figure 7:**
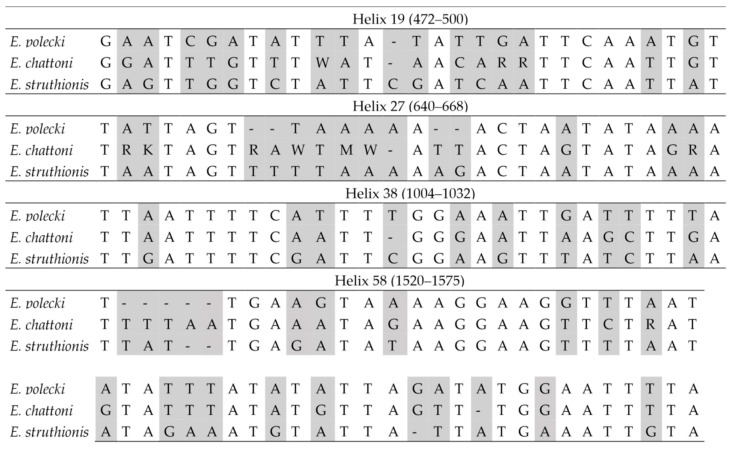
Barcodes in the SSU-rDNA sequence for the differentiation between *Entamoeba polecki*, *Entamoeba chattoni* and *Entamoeba struthionis*. Base positions and helix numbers are according to the secondary structure in Figure 4. Differential positions are shadowed.

**Figure 8:**
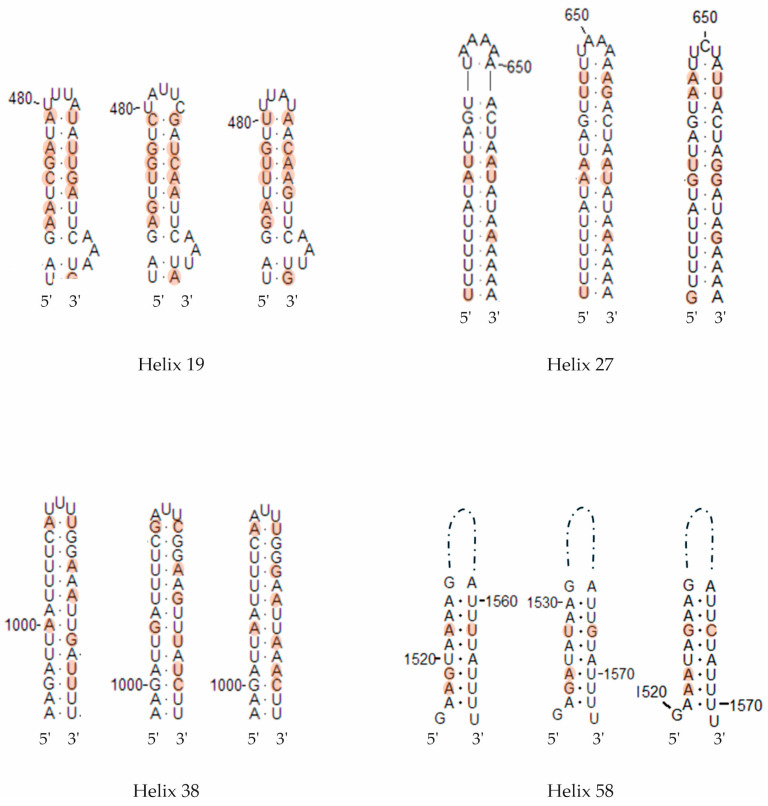
Secondary structure diagrams of the SSU rRNA helices showing compensatory base changes between *Entamoeba polecki* (left in each helix group), *Entamoeba chattoni* (center) and *Entamoeba struthionis* (right). Positions with differences between sequences are colored. Base positions and helix numbers are according to the secondary structure in Figure 4.

In interspecific comparisons within *E. polecki* s.l., six CBCs were detected between *E. polecki* and *E. struthionis*, five between *E. polecki* and *E. chattoni*, and seven between *E. struthionis* and *E. chattoni*. The most pronounced differences, both at the primary sequence level and in terms of CBCs, were concentrated in helices 19, 27, 38, and 58 (Figure 7 and Figure 8).

## 4. Discussion

Since the late 20th century, genetic data have been increasingly applied in taxonomic and epidemiological studies of *Entamoeba*. However, no clearly defined and uniformly applied criteria have been established across all putative species, a situation that has, in some cases, generated taxonomic controversy, such as the proposed synonymy of *E. chattoni* and *E. struthionis* with *E. polecki* [13,51]. The first explicit proposal for incorporating genetic data into the taxonomy of the genus [20] was primarily aimed at classifying existing variants rather than at defining explicit criteria for species delimitation.

In the present study, we show that objective criteria based on quantitative analyses of the SSU rRNA gene can be applied to species delimitation in *Entamoeba*, while also explicitly considering the limitations and potential pitfalls of this approach. When integrated with evidence from other commonly used criteria, particularly morphology and host association, these genetic and structural analyses provide a robust and objective framework for species delimitation and identification within the genus *Entamoeba*.

### 4.1. On the Species Concept on Entamoeba

In *Entamoeba*, as in many strictly or predominantly asexual protists, the choice of a species concept has direct practical consequences for how diversity is recognized and delimited. The biological species concept, which defines species as reproductively isolated interbreeding populations [52], is not applicable in practice, as meiotic sex and gene flow have neither been observed nor experimentally demonstrated in this genus. Although indirect evidence suggesting the possibility of sexual reproduction has been reported [53,54], this remains unconfirmed. Consequently, species concepts relying on reproductive isolation provide little guidance for species delimitation in *Entamoeba*.

Morphology-based species concepts are likewise insufficient, as illustrated by morphologically indistinguishable but widely accepted species such as *E. histolytica* and *E. dispar*. Ecological criteria offer limited improvement. Although host association might appear informative, host ranges are often broad and overlapping: the same *Entamoeba* species may infect phylogenetically distant hosts, as shown for *E. dispar* in humans, non-human primates [11], anteaters, and rheas [22], while a single host species may harbour multiple *Entamoeba* species. Humans, for example, can host numerous intestinal species (*E. polecki*, *E. chattoni*, *E. struthionis*, *E. hartmanni*, *E. dispar*, *E. bangladeshi*, *E. moshkovskii*, *E. histolytica*, *E. nuttalli*, and *E. coli*), as well as *E. gingivalis* in the oral cavity.

Geographic criteria are similarly of limited utility in parasitic taxa associated with humans and domestic animals, whose distributions have been extensively homogenized by human-mediated dispersal. Taken together, morphology, host specificity, and geography—whether considered individually or in combination—do not provide a general or consistent framework for species delimitation in *Entamoeba*, highlighting the need for alternative criteria grounded in genetic and evolutionary evidence.

Under these conditions, lineage-based species concepts are particularly appropriate. The evolutionary species concept [55], the general or unified species concept [56], and the pragmatic species concept [57] all define species as independently evolving metapopulation lineages. Within this framework, properties such as reproductive isolation, diagnosability, ecological divergence, or monophyly are treated as lines of evidence for lineage independence rather than as defining criteria. This perspective is well suited to asexual protists and allows the integration of genetic, morphological, ecological, and host-related data, consistent with the principles of integrative taxonomy [58].

Theoretical and empirical work supports the applicability of this approach to asexual organisms. Barraclough et al. (2003) [59] showed that strictly asexual lineages are expected to form distinct genotypic clusters that can be interpreted as independently evolving units. Building on this framework, Birky and colleagues developed explicit population-genetic criteria for species delimitation in asexual eukaryotes using DNA sequence data alone [47,60], demonstrating that species can be identified based on patterns of within-lineage coalescence and between-lineage divergence. The widespread occurrence of cryptic species further underscores the importance of genetic data as more than an auxiliary tool in taxonomy [61]. In *Entamoeba*, cryptic diversity has been proposed for several taxa [23,62].

Together, these considerations support the adoption of an explicitly evolutionary, lineage-based species concept for *Entamoeba*, in which genetic differences are accepted as valid and informative criteria for species delimitation. Within this framework, morphology, host range, and geographic distribution remain valuable when they differ among species, but species hypotheses may be formulated primarily on genetic evidence when these characters are ambiguous or non-diagnostic. This approach is fully consistent with integrative taxonomy, which emphasizes the use of multiple complementary datasets rather than reliance on any single class of characters [63,64,65].

### 4.2. On the Use of Genetic Data for Species Delimitation in Entamoeba

Genetic data are increasingly central to taxonomic practice, and in some protist groups, such as ciliates, gene sequence analysis is now considered essential for species descriptions and redescriptions [66]. It is, however, important to distinguish between the use of genetic markers for phylogenetic inference and for species delimitation. In sexual organisms, gene trees may differ from species trees due to recombination, gene flow, and incomplete lineage sorting [67]. In contrast, in asexual organisms all genes are effectively linked and evolve as a single unit, so the phylogeny inferred from a single gene is expected to reflect genome-wide evolutionary history [60].

Within this context, and under an evolutionary species concept, a single genetic marker may be sufficient to demonstrate independent evolutionary trajectories and delimit species in asexual organisms [68]. The critical questions then concern marker choice and the nature and magnitude of genetic differences that should be interpreted as evidence of species boundaries. Distance-based approaches, including genetic divergence thresholds and barcoding-gap analyses, represent one possible strategy, but have been criticized for relying on similarity measures rather than diagnostic characters [1,69,70]. Following the synthesis of Miralles et al. (2024) [58], genetic distances should therefore be viewed as an exploratory tool, while robust species delimitation requires the identification of explicit quantitative or qualitative characters supporting lineage independence.

A further issue is whether sequence data alone are sufficient for the formal description of a new species. The International Code of Zoological Nomenclature (the Code) [71] requires that species be diagnosed using characters stated in words (Art. 13.1.1), without restricting their nature. However, under the current Code regulations, species names must be anchored to a name-bearing type, typically a preserved specimen deposited in a public collection (Arts. 16.4, 72.5, 73.1). The Code allows hapantotypes (Art. 72.5.4), microscopic slides (Art. 72.5.5), and, under specific circumstances, illustrations or photographs of a single individual as holotypes (Art. 73.1.4; Declaration 45 [72]). Article 72.5.1 defines eligible name-bearing types broadly as “an animal [used in a broad sense, to include also microeukaryotes], or any part of an animal”. It can be argued that a physically deposited total DNA extract (traceable to the sampled individual(s) and curated in a recognized repository) may qualify as “part of an animal” under this provision and consequently it may theoretically serve as a type; there are no statements in Art. 73 or its recommendations that preclude it. However, sequences obtained by a copying process such as PCR before sequencing are not part of the organism nor does it fit other type-compatible categories, but they can be considered as a description of the organism DNA [73]. The practical implications of DNA-based taxonomy and the potential role of archived DNA material as taxonomic reference are currently under active discussion [74] and there are recommendations for future editions of Codes of bionomenclature for tightening of the definition of “species diagnosis” to ensure that all diagnoses, including DNA-based ones, are adequate [75]. At this moment, petitions to adopt DNA sequences as types are currently not being considered at least by the framers of the two Codes that cater to eukaryotes (International Commission of Zoological Nomenclature and International Commission of Nomenclature of Algae, Fungi and Plants) [75].

While total DNA extract could serve as type material, the type of DNA extract could also be problematic, especially in *Entamoeba* in which many species are not cultivable in vitro and the DNA is obtained from fecal samples in which other organisms (even, other *Entamoeba* species) are present. Again under the current Code, environmental (fecal) samples or the DNA extracted from them (eDNA) are questionable because they represent a mixture of organisms and it is challenging to distinguish the focal organisms from those of co-occurring species [74].

Accordingly, in *Entamoeba* and under the present Code, sequence data alone are not accepted as types [74,75] and consequently they are insufficient to support a valid nomenclatural act. Best practice remains to anchor new names to a deposited hapantotype, slides, or images, and if possible, an archived DNA extract deposited in a curated collection to facilitate future reinvestigation with additional methods. Establishing new taxa without preserved type material is permissible but discouraged and should be explicitly justified when unavoidable (Recommendation 73G [72]). In practice, illustrations or micrographs (often referred to as iconotypes, e-types, or phototypes; [76]) have been widely used and accepted in *Entamoeba* taxonomy. Following Stensvold et al. (2011) [20], morphological documentation should be considered mandatory for assigning a formal taxonomic name to a genetically identified lineage and can be fulfilled by depositing a hapantotype, slide, or explicitly designated illustration or photograph (typically of the cyst stage). When only gene sequence(s) and host data are available, the absence of a name-bearing type precludes formal species description, and by the moment, the use of ribosomal lineage (RL) terminology remains appropriate.

In this context, we propose replacing the current numerical RL designation system with a name-based system. This proposal is motivated by several considerations. First, names are easier to remember and communicate than numerical identifiers. Second, if an RL is later elevated to species rank, the proposed name may, if desired, serve as the basis for the specific epithet. Third, the use of names avoids discontinuities or gaps that may arise in a purely numerical system when lineages are reclassified or promoted.

### 4.3. On the Use of the SSU-rRNA Gene Sequences for Species Delimitation in Entamoeba: Limitations and Sources of Bias

Only a limited number of nuclear genetic markers have been explored in *Entamoeba*, and for most of them sequence data are available for only a small subset of species. At present, the SSU rRNA gene is the only marker widely used for species-level comparative analyses in this genus. This is largely due to its multicopy nature, the presence of conserved regions suitable for primer design, and its relatively rapid rate of evolution in *Entamoeba*, which results in long branches in phylogenetic trees and provides sufficient resolution for taxonomic differentiation using a single locus [11].

Before addressing the specific limitations associated with the use of the SSU rRNA gene in *Entamoeba*, it is important to note that intragenomic variation in multicopy genes may arise from multiple sources, thus leading to sequence differences between individuals/isolates that are not directly related to species divergence. In most eukaryotes, rRNA genes occur as tandem repeats and are often described as evolving under concerted evolution, i.e., the tendency of repeated copies to become homogenized through recombination-driven processes such as unequal crossing-over and gene conversion [77,78]. There is a general consensus that concerted evolution is the rule in multicopy genes, as the rRNA genes [79]; as a result, the variation is usually considered minimal within individuals but may be substantial between lineages and species [80]. However, evidence from broad comparative surveys and genomic analyses shows that rDNA homogenization is frequently incomplete and that substantial intragenomic rDNA variation can persist [81]. In asexual or predominantly clonal lineages, the absence of meiotic recombination may further reduce the efficiency of concerted evolution, favouring the long-term persistence of divergent intragenomic rRNA variants [81]. Experimental evidence further indicates that different rRNA gene copies within the same genome may be subject to different selective pressures, allowing heterogeneous variants to persist [82], and that intragenomic rRNA heterogeneity is not necessarily neutral, as differential expression of rRNA variants and functionally specialized ribosomes have been described in both prokaryotic and eukaryotic organisms [83,84,85]. Importantly, the intragenomic heterogeneity may include non-functional rDNA copies (pseudogenes) or partially degraded rDNA fragments originated by mutations [86] or as by-products of concerted evolution [87], which can be co-amplified and contribute to apparent within-taxon diversity if not recognized.

This general framework is particularly relevant for *Entamoeba*, where canonical assumptions about chromosomal tandem arrays may not apply because rRNA genes are located on extrachromosomal circular DNA molecules rather than in chromosomal arrays [88]. Such an unusual genomic organization is expected to modify rDNA homogenization dynamics and may facilitate the persistence of divergent intragenomic rRNA variants. In addition, further complexity arises because in protists, and almost invariably in *Entamoeba*, sequence data are derived from isolates composed of multiple clones rather than from single cells or clonal cultures; consequently, the observed variability may reflect a combination of intragenomic variation, intraclonal heterogeneity, and interclonal diversity. The origin of sequence heterogeneity within samples of the same species (whether arising from differences among individual genomes or from variability within single genomes) is of limited relevance for molecular systematics and species identification; the key issue is whether such variability can bias species delimitation, compromise diagnostic assignments, or affect phylogenetic inference [80].

Taken together, these sources of genetic heterogeneity define a complex background against which SSU rRNA variation must be interpreted. However, acknowledging the existence of multiple potential origins of sequence variability does not preclude the use of SSU rRNA data for species delimitation, but rather emphasizes the need to identify which sources of variation are most likely to introduce systematic bias under the conditions of the available data. In practice, this requires distinguishing background biological variability from methodological and annotation-related artefacts that can be explicitly detected, quantified, and mitigated.

A major limitation of SSU rRNA–based analyses, also affecting distance-based approaches in general [58], is the reliability of taxonomic identifications in public databases. A substantial proportion of *Entamoeba* sequences deposited in GenBank and annotated at the species level are incorrectly identified. In our dataset, more than 5% of the analysed sequences corresponded to organisms unrelated to *Entamoeba*, including host sequences, and excluding identifications within *E. polecki* s.l., more than 7% were assigned to the wrong species. Overall, approximately 12% of the analysed records were incorrectly annotated. Such levels of taxonomic error are consistent with previous reports documenting widespread misassignments in public databases [89,90,91,92,93,94], with species-level error rates reaching up to 17% [93]. The use of carefully curated reference datasets therefore represents a practical solution, allowing the application of objective criteria (e.g., the 4× rule or empirically derived distance cut-offs) to assign sequences to recognised species or RLs and to identify candidates for novel lineages.

A second issue concerns sequence quality. Most discrepancies between downloaded sequences and reference sequences were located at the 5′ and 3′ ends, consistent with well-known PCR and sequencing artefacts, including declining read quality and increased error rates toward fragment ends in both Sanger and next-generation sequencing [95,96,97], as well as base-calling errors commonly observed in chromatograms [98,99]. Primer mismatches and suboptimal PCR conditions further contribute to artefactual variation [100]. Also, variations in primer sequences from different studies may contribute to variability located in the sequence ends. To minimize these effects, highly variable terminal regions were trimmed when necessary, and genetic distances were calculated only between sequences overlapping by at least 100 nucleotides. Nevertheless, when short sequences are included, terminal artefacts may map onto internal alignment positions, artificially inflating apparent nucleotide variability. This explains why within-species genetic distances were substantially higher when both long and short sequences were analysed, particularly in *E. histolytica*, and why some species pairs failed the 4× criterion in overlap-filtered datasets. These failures are best explained by artefactual inflation of within-species diversity rather than by genuine evolutionary proximity.

Despite these limitations, genetic divergence remains a valid and informative preliminary tool for lineage exploration [58]. In strictly asexual organisms, within-lineage divergence is constrained, and deep divergence gaps are unlikely to arise through stochastic processes alone [47]. The 4× rule formalizes this expectation, indicating independent evolutionary trajectories when K/θ ≥ 4 [47,60]. However, the 4× rule was originally designed for assigning individuals to species, not for detecting cryptic diversity within nominal species. In this context, absolute distance cut-offs are useful for identifying potential independently evolving lineages. The π-based threshold of 0.010 proposed here is more conservative than the 4× rule and consequently may fail to resolve very recent splits; values below this threshold should therefore not be interpreted as evidence of conspecificity.

The proposed distance cut-off should be regarded as an empirical threshold reflecting a biologically meaningful transition between intra- and interspecific variability. Although the permutation test used to identify the breakpoint lacks power and relies on a biologically unrealistic null model, it provides methodological transparency. In contrast, non-parametric tests (Kruskal–Wallis, Jonckheere–Terpstra, and Wilcoxon) consistently revealed significant increases in genetic distances with the addition of further species, supporting the biological relevance of the inferred threshold.

As emphasized above, genetic distances should represent an initial exploratory step rather than a stand-alone criterion [58]. The current system for defining RLs and STs in *Entamoeba* [20,21] relies largely on bootstrap support in phylogenetic analyses and implicitly on genetic distances, without explicitly defined diagnostic characters. From a taxonomic perspective, this is problematic, as RLs and STs may at some moment be designated as distinct species [21]. To reduce this ambiguity, species delimitation should be based on comparative sequence analyses complemented by SSU rRNA secondary-structure information.

To ensure reliable alignments, secondary-structure information was explicitly used as a guide. The use of rRNA secondary structure to improve alignment accuracy has long been advocated [101,102,103,104,105]. The SSU rRNA secondary-structure model employed here, partially based on experimental data [33] and completed using congruent in silico predictions, is consistent with previous models [106] and transferable across *Entamoeba* species. Although minor variations occur (e.g., missing helices in *E. gingivalis* or *E. polecki* s.l.), overall structural conservation facilitates accurate indel placement in alignments of sequences of the same or closely related species and reduces artefactual inflation of genetic distances.

Mutational processes operate continuously, but fixation probabilities vary according to functional constraints [107,108]. In SSU rRNA, evolutionary rates vary among structural elements [109] and functionally critical regions are highly conserved, whereas higher variability is concentrated in expansion segments (ES1–ES12) or variable regions V1–V9 [110,111,112,113]. Several of these regions, notably V4 and V9 (ES6 and ES12), are commonly used in protist taxonomy [114,115,116,117,118]. In *Entamoeba*, interspecific variability is concentrated in ES2–ES3/V2, ES5/V3, ES6/V4, ES7/V5, ES9/V7, ES10/V8, and ES12/V9 [106]. By contrast, the intraspecific variability observed in this study was distributed across the entire gene, including conserved regions, strongly suggesting an artefactual origin linked to sequencing errors and short fragments. These patterns indicate that no single SSU rRNA region can serve as a universal barcoding fragment for *Entamoeba*. Recently diverged species may lack differences in predefined regions, whereas more divergent taxa may accumulate substitutions across multiple segments. Reliance on short fragments therefore risks overlooking valid species or generating artificial sublineages. This explains discrepancies among studies targeting different gene regions and reinforces the need for integrative, structure-aware analyses of the full SSU rRNA gene.

### 4.4. Application of SSU rRNA Gene Sequences to Species Delimitation in Entamoeba

Once divergence analyses (AMOVA, ASAP) suggest that sequences may represent distinct lineages, validation using non–distance-based criteria is required. Alfonso et al. (2012) [106] proposed the use of CBCs in *Entamoeba* SSU rRNA secondary structures, a criterion extensively applied in ITS2 analyses [119,120,121,122,123,124,125] but rarely explored in SSU rRNA [104,118]. In *Entamoeba*, we have observed that intraspecific variability did not result in random CBCs, except in a single partial sequence of *E. chattoni*, where variability was restricted to a highly conserved region in this genus [106], suggesting technical artefacts or ribosomal pseudogenes rather than true lineage divergence.

Given that well-established species such as *E. histolytica* and *E. dispar* differ by only one CBC, we consider the presence of a single CBC to be the minimum structural requirement to support independent evolutionary lineages. To avoid spurious species recognition based on chance CBCs arising from limited divergence, CBCs are evaluated in combination with a minimum divergence threshold (>0.010 or the 4× rule). This integrated framework reduces unjustified lineage splitting and promotes a consistent interpretation of substitution accumulation across species. Under this framework, RLs are delimited using objective divergence criteria supported by explicit structural characters, replacing the subjective bootstrap-based criteria previously applied [20,21]. The performance and limitations of this framework can be further assessed when applied to closely related taxa within specific phylogenetic contexts.

### 4.5. On the Species Validity Within the Entamoeba Histolytica-like Clade

The distinction between species delimitation and species differentiation becomes particularly relevant when applying the proposed framework to closely related taxa within the *E. histolytica*-like clade. Species differentiation may rely on diagnostic sequence barcodes that allow practical discrimination among lineages, even when divergence is shallow. In contrast, species delimitation requires evidence that lineages have evolved independently for a sufficient period of time to reasonably ensure that they correspond to separate and independent populations. Under our SSU rRNA gene-based framework, *E. histolytica* and *E. dispar* are clearly distinguished by both genetic divergence and structural criteria. In contrast, although analyses of genetic divergence support the recognition of *E. nuttalli* as a distinct lineage, the structural criterion does not consistently separate this species from *E. histolytica* or *E. dispar*. Within *E. nuttalli*, overall genetic variability is low, and depending on the sequence variant considered, a CBC may differentiate it from *E. histolytica* but not from *E. dispar*, or may be absent altogether. This indicates that, in this case, species delimitation cannot be reliable based on the SSU rRNA gene alone and requires the use of additional or alternative markers. Independent evidence supporting the species status of *E. nuttalli* has been provided by Tachibana and colleagues [14,126].

This pattern illustrates an inherent limitation of a deliberately conservative framework that requires both sufficient genetic divergence and the presence of at least one CBC. As expected under evolutionary theory, even lineages that diverged millions of years ago (the split between *E. histolytica* and *E. nuttalli* has been estimated at approximately 5.93 ± 0.28 million years ago [43]) may exhibit shallow contemporary diversity if extant variation coalesces to a much more recent common ancestor (in *E. histolytica*, approximately 160,000 years ago [23]) due to demographic processes, lineage turnover, or genetic drift. Consequently, recently diverged species (or species with limited present-day diversity) may not yet exhibit strong structural signals such as CBCs, nor exceed conservative divergence thresholds. This does not contradict the evolutionary or pragmatic species concepts applied to *Entamoeba* but rather highlights that young lineages may require additional or alternative markers for reliable delimitation. Importantly, when both genetic divergence and CBC thresholds are met, they provide high-confidence evidence of species-level independence even if no other gene markers have been considered. Conversely, failure to meet these criteria should not be interpreted as evidence of conspecificity, but as an indicator that additional data are required.

### 4.6. On Species Validity Within Entamoeba Polecki s.l.

As in the *Entamoeba histolytica*-like clade, the distinction between species delimitation and species differentiation is central to evaluating taxonomic hypotheses within *E. polecki* s.l. However, in contrast to the former group, the taxonomic history of the pig amoebae is marked by long-standing instability and by the inconsistent application of species criteria since their earliest descriptions [6].

Early classifications grouped all uninucleate *Entamoeba* species infecting cattle, goats, sheep, and pigs within *E. polecki* [9]. Subsequent molecular studies demonstrated that the *Entamoeba* species infecting ruminants represent distinct taxa, now generally recognised as *E. bovis* [21,28], whereas the species infecting pigs are identified as *E. polecki* or *E. suis* depending on their SSU rRNA sequences [13]. In parallel, *E. chattoni* and *E. struthionis* were described as separate species [16,127] and distinct SSU rRNA sequences are available [16,25].

The subsequent proposal to synonymise *E. chattoni* and *E. struthionis* with *E. polecki* [13] was motivated by their phylogenetic proximity and by their detection in human samples [128]. However, as previously noted [51], this proposal effectively applied different standards to uninucleate species than to tetranucleate cyst-forming species such as *E. histolytica*, *E. nuttalli*, and *E. dispar*, despite comparable or greater levels of genetic divergence. Moreover, the synonymization was not supported by explicit analytical criteria, but rather justified by a perceived lack of host specificity and by a “small amount of sequence divergence” [11] (p. 286), [129].

The acceptance of this taxonomic interpretation was reinforced in the subspecific nomenclature of *Entamoeba* [20], whereby “well-supported clusters within a defined species” were designated as STs. Under this framework, *E. polecki*, *E. struthionis*, and *E. chattoni* were treated as *E. polecki* subtypes. This nomenclature has been adopted primarily by these authors and collaborators [11,21,130,131] and subsequently used in several studies [132,133,134,135]. At the same time, other authors have continued to recognise the original species [18,136,137,138,139] or have employed mixed nomenclatures combining species names and ST designations [126,140]. Notably, Stensvold et al. (2023) [141] have more recently acknowledged that many STs likely correspond to distinct species, and that the terminology reflects the presence of a species complex rather than a single species exhibiting cryptic diversity.

When the framework proposed here is applied to *E. polecki* s.l., the results provide consistent and strong support for species-level differentiation. Clear genetic and structural differences were observed among sequences assigned to *E. polecki*, *E. struthionis*, and *E. chattoni*, with statistical support exceeding that observed among species within the *E. histolytica*-like clade. In the comparison between *E. polecki* and *E. struthionis*, the most closely related taxa within this group, the π value of the combined alignment exceeds the proposed cut-off threshold, both AMOVA and partitioning analyses support their separation as independent lineages, and six CBCs were identified in comparisons of their SSU rRNA secondary structures. Taken together, these results satisfy both components of the delimitation framework applied in this study (minimum genetic divergence and the presence of CBCs) and therefore provide high-confidence evidence that *E. polecki*, *E. struthionis*, and *E. chattoni* represent distinct biological entities. In contrast to the situation observed for *E. nuttalli* within the *E. histolytica*-like clade, species delimitation within *E. polecki* s.l. is not constrained by shallow divergence or ambiguous structural signals, but instead reflects deeper and more consistent evolutionary separation.

## 5. Conclusions

It is essential that researchers ensure both the quality of sequence data and the correct assignment of sequences to species before submitting them to public databases. The high rate of erroneous assignments detected in this study (approximately 12%) should be regarded as a warning to both data submitters and database users, highlighting the need for greater caution in sequence annotation and interpretation.

We propose an evolutionary species concept for *Entamoeba* that explicitly allows the use of genetic data within an integrative taxonomic framework, treating molecular evidence as an independent and equally valid criterion for species delimitation, rather than as subordinate to morphological traits or host association. Within this framework, we applied a novel approach based on the analysis of genetic diversity in SSU rRNA gene sequences as an initial step to detect levels of intraspecific variability compatible with the presence of cryptic species, and to assess whether the sequence of a given isolate corresponds to a previously described species or ribosomal lineage. This method incorporates quantitative parameters that provide a more objective basis for sequence assignment than the distance-based criteria underlying the framework currently in use.

Our results indicate that there is no single, predefined region of the SSU rRNA gene that can serve as a universal target for species differentiation or delimitation in *Entamoeba*. Instead, the entire gene should be considered, as substitutions and compensatory base changes may occur throughout its length. Although our approach based on a genetic diversity cut-off value may be less sensitive to detecting very recent divergence than Birky’s 4× rule, it offers a more robust and conservative basis for species validation. When combined with the detection of structural changes (specifically compensatory base changes) in the secondary structure of SSU rRNA molecules, this framework provides a practical and reproducible method for species delimitation based on genetic data. While broadly applicable, it is particularly useful in situations where cryptic species are likely to occur. Applying this approach, we demonstrate that *E. polecki*, *E. struthionis*, and *E. chattoni* represent distinct species rather than subtypes of a single taxon.

Finally, we propose refined secondary-structure models of the SSU rRNA molecules for *Entamoeba* species, developed through a combination of experimental evidence and in silico analyses. These models are essential for the accurate alignment of related sequences (particularly in regions containing insertions and deletions) and represent a valuable resource for improving alignment quality in phylogenetic and taxonomic studies.

## Figures and Tables

**Table 1:** SSU rDNA sequences of *Entamoeba* species used as the reference dataset in this study. Sequences were obtained from the original species description or from the earliest available publication reporting the complete SSU rDNA sequence for the species. Species are grouped according to morphological categories (following [10]) and are listed in alphabetical order.

*Entamoeba* Species	Accession Number	Reference
Tetranucleate mature cysts		
*E. bangladeshi*	KR025411	[21]
*E. dispar*	Z49256	[24]
*E. ecuadoriensis*	DQ286373	[13]
*E. equi*	DQ286371	[13]
*E. hartmanni*	AF149907	[25]
*E. histolytica*	X65163	[26]
*E. insolita*	AF149909	[25]
*E. invadens*	AF149905	[25]
*E. marina*	LC031816	[18]
*E. moshkovskii*	AF149906	[25]
*E. nuttalli*	AB282657	[14]
*E. ranarum*	AF149908	[25]
*E. terrapinae*	AF149910	[25]
Octonucleate mature cysts		
*E. coli*	AF149914	[25]
*E. muris*	AB445018	[27]
Uninucleate mature cysts		
*E. bovis*	FN666248	[28]
E. chattoni *	AF149912	[25]
*E. chiangraiensis*	MK652887	[19]
*E. polecki* sensu stricto *	AF149913	[25]
E. struthionis *	AJ566411	[16]
*E. suis*	DQ286372	[13]
No cyst-forming species		
*E. gingivalis*	D28490	[29]

* For *Entamoeba polecki* sensu lato, the reference sequence is that of *E. polecki* sensu stricto.

**Table 2:** Number of SSU rDNA sequences of *Entamoeba* species available in GenBank and included in this study.

*Entamoeba* Species	Number of Sequences Downloaded (*n* = 1844)	Number of Valid Sequences After Reassignment (*n* = 1746)	Nucleotide Diversity (π) (Mean ± Standard Deviation)
*E. histolytica*	310	167	0.00628 ± 0.02194
*E. nuttalli*	13	126	0.00319 ± 0.00372
*E. dispar*	90	100	0.00396 ± 0.00731
*E. bangladeshi*	7	7	0.00172 ± 0.00135
*E. moshkovskii*	181	183	0.01166 ± 0.01780
*E. harmanni*	76	75	0.02817 ± 0.03423
*E. invadens*	12	12	0.00170 ± 0.00304
*E. ranarum*	7	7	0.02269 ± 0.02032
*E. terrapinae*	6	6	0.10569 ± 0.12274
*E. suis*	53	53	0.00276 ± 0.00444
*E. polecki sensu stricto*	130	40	0.00316 ± 0.00726
*E. chattoni*	8	60	0.01846 ± 0.02989
*E. struthionis*	4	41	0.00123 ± 0.00240
*E. bovis*	489	489	0.08290 ± 0.03280
*E. coli*	197	120	0.15670 ± 0.16325
*E. gingivalis*	251	251	0.10435 ± 0.10963
*E. chiangraiensis*	2	2	-
*E. ecuadoriensis*	2	1	-
*E. equi*	2	2	-
*E. marina*	1	1	-
*E. muris*	2	2	-
*E. insolita*	1	1	-

**Table 3:** Nucleotide diversity (π) estimated for individual species and cumulative species combinations within the *Entamoeba histolytica*-like clade and *Entamoeba polecki* s.l. Analyses were performed using two datasets: one including pairwise genetic distances derived from sequences overlapping at least 100 nucleotide positions, and another including distances from sequences covering more than 75% of the alignment length.

	Dataset: 100 Bases Overlap		Dataset: >75% Alignment Coverture	
Species in the Alignment	Number of Sequences	Nucleotide Diversity (π) (Mean ± Standard Deviation)	Number of Sequences	Nucleotide Diversity (π) (Mean ± Standard Deviation)
*Entamoeba histolytica*-like clade				
*Entamoeba histolytica*	167	0.00648 ± 0.02194	20	0.00041 ± 0.00122
*Entamoeba nuttalli*	126	0.00319 ± 0.00372	13	0.00023 ± 0.00039
*Entamoeba dispar*	100	0.00396 ± 0.00731	13	0.00189 ± 0.00123
*Entamoeba bangladeshi*	7	0.00172 ± 0.00135	2	0.00058
*Entamoeba moshkovskii*	183	0.01166 ± 0.01780	26	0.03092 ± 0.01920
*E. histolytica + E. nuttalli* (2SP)	293	0.00820 ± 0.01591	33	0.00408 ± 0.00387
2SP* + E. dispar* (3SP)	393	0.01259 ± 0.01654	46	0.01044 ± 0.00833
3SP * + E. bangladeshi* (4SP)	400	0.01492 ± 0.02094	48	0.01702 ± 0.02339
4SP * + E. moshkovski* (5SP)	583	0.07735 ± 0.09114	74	0.10318 ± 0.09079
*Entamoeba polecki* sensu lato				
*Entamoeba polecki* sensu stricto	40	0.00316 ± 0.00726	17	0.00134 ± 0.00150
*Entamoeba struthionis*	41	0.00123 ± 0.00240	11	0.00490 ± 0.00682
*Entamoeba chattoni*	60	0.01846 ± 0.02989	11	0.00169 ± 0.00142
*E. polecki* s.s. + *E. struthionis* (2SP)	81	0.03505 ± 0.05345	28	0.02426 ± 0.02272
*2SP+ E. chattoni* (*E. polecki s.l.*)	141	0.06372 ± 0.07897	39	0.03541 ± 0.02383

**Table 4:** Summary of statistical tests applied to pairwise genetic distance distributions in single-species and combined-species alignments of the *Entamoeba histolytica*-like clade. Pairwise distances were filtered to retain only comparisons between sequences overlapping by at least 100 nucleotide positions.

Analysis	Test	Statistic	*p*-Value
Increased trend in genetic distance across the combined alignments	Jonckheere–Terpstra	JT = 2.07 × 10^10^	<0.001
Comparison of all genetic distances’ distributions (single and mixed species alignments)	Kruskal–Wallis	χ^2^ = 62,633 df = 8	<0.001
Post hoc comparisons—single vs. combined species distributions (20 contrasts)	Wilcoxon (Holm correction)		<0.001 (all)
Combined vs. combined species distributions (6 contrasts)			<0.001 (all)

**Table 5:** Summary of statistical tests applied to pairwise genetic distance distributions in single-species and combined-species alignments of the *Entamoeba histolytica*-like clade. Pairwise distances were filtered to retain only comparisons between sequences covering at least 1500 nucleotide positions.

Analysis	Test	Statistic	*p*-Value
Increased trend in genetic distance across the combined alignments	Jonckheere–Terpstra	JT = 9.28 × 10^6^	<0.001
Comparison of all genetic distances’ distributions (single and mixed species alignments)	Kruskal–Wallis	χ^2^ = 2061 df = 8	<0.001
Post hoc comparisons—single vs. combined species distributions (20 contrasts)	Wilcoxon (Holm correction)		<0.001 (all) (*)
Combined vs. combined species distributions (6 contrasts)			<0.001 (all)

(*) Comparisons involving *E. bangladeshi* returned nonsignificant *p*-values (*p* > 0.05) in all cases. These results are attributable to the extremely low number of valid pairwise distances for this species (two sequences, *n* = 1 pairwise distance in the ≥1500 bases dataset), which limits the statistical power of rank-based tests.

**Table 6:** Summary of change-point analyses based on minimization of the within-segment sum of squared errors (SSE) applied to nucleotide diversity (π) in the *Entamoeba histolytica*-like clade. Codes: sp1: *Entamoeba histolytica*; sp2: *Entamoeba nuttalli*; sp3: *Entamoeba dispar*; sp4: *Entamoeba bangladeshi*.

Dataset	Series Examined	Change-Point Position (SSE)	π Values at the Change Point	*p*-Value (Permutation Test)
Overlap-filtered	sp1/(sp1 + sp2)/(sp1 + sp2 + sp3)/(sp1 + sp2 + sp3 + sp4)	(sp1 + sp2)/(sp1 + sp2 + sp3)	0.00820 → 0.01259	0.327
Length-filtered		(sp1 + sp2)/(sp1 + sp2 + sp3)	0.00408 → 0.01044	0.330

**Table 7:** Results of applying the 4× rule to all species pairs (A-B) in the *Entamoeba histolytica*-like clade. Values in the upper-right side correspond to the length-filtered dataset (≥75% alignment coverage) and values in the lower-left cells correspond to the overlap-filtered dataset (≥100 bp overlap). Values shown are mean interspecific distance (K_AB_) with 95% confidence interval (CI) from filtered pairwise comparisons between sequences of A and B, and the ratio R = K_AB_/θ_AB_ with its 95% CI. The θ_AB_ values correspond to the higher π_A_-π_B_ (Table 3).

	*E. histolytica*	*E. nuttalli*	*E. dispar*	*E. bangladeshi*	*E. moshkovskii*
*E. histolytica*	-	K_AB_ = 0.00787 (0.00779–0.00797)	K_AB_ = 0.02081 (0.02056–0.02108)	K_AB_ = 0.09620 (0.09444–0.09781)	K_AB_ = 0.20223 (0.20092–0.20351)
		R = 19.33 (13.24–31.52)	R = 11.03 (9.48–13.20)	R = 165.00 (161.19–167.79)	R = 6.54 (6.14–7.01)
*E. nuttalli*	K_AB_ = 0.01164 (0.01141–0.01188)	-	K_AB_ = 0.01708 (0.01696–0.01719)	K_AB_ = 0.08875 (0.08777–0.08983)	K_AB_ = 0.19577 (0.19433–0.19703)
	R = 1.79 (1.67–1.94)		R = 9.05 (7.79–10.94)	R = 152.22 (150.57–154.11)	R = 6.33 (5.92–6.78)
*E. dispar*	K_AB_ = 0.02325 (0.02290–0.02361)	K_AB_ = 0.02042 (0.02021–0.02063)	-	K_AB_ = 0.07992 (0.07833–0.08135)	K_AB_ = 0.18723 (0.18572–0.18877)
	R = 3.59 (3.33–3.86)	R = 5.16 (4.82–5.51)		R = 42.36 (36.20–50.85)	R = 6.05 (5.67–6.54)
*E. bangladeshi*	K_AB_ = 0.07055 (0.06796–0.07340)	K_AB_ = 0.06194 (0.05978–0.06418)	K_AB_ = 0.05859 (0.05608–0.06135)	-	K_AB_ = 0.22403 (0.21871–0.22927)
	R = 10.88 (10.08–11.81)	R = 19.40 (18.49–20.32)	R = 14.80 (13.61–16.13)		R = 7.25 (6.73–7.77)
*E. moshkovskii*	K_AB_ = 0.01652 (0.16524–0.16783)	K_AB_ = 0.13578 (0.13491–0.13665)	K_AB_ = 0.13976 (0.13839–0.14123)	K_AB_ = 0.11923 (0.11492–0.12364)	-
	R = 14.28 (13.89–14.64)	R = 11.64 (11.35–11.95)	R = 11.98 (11.67–12.28)	R = 10.22 (9.79–10.68)	

**Table 8:** Results of applying the 4× rule to all species pairs (A-B) within *Entamoeba polecki* sensu lato. Values in the upper-right side correspond to the length-filtered dataset (≥75% alignment coverage) and values in the lower-left cells correspond to the overlap-filtered dataset (≥100 bp overlap). Values shown are mean interspecific distance (K_AB_) with 95% confidence interval (CI) from filtered pairwise comparisons between sequences of A and B, and the ratio R = K_AB_/θ_AB_ with its 95% CI. The θ_AB_ values correspond to the higher π_A_-π_B_ (Table 3).

	*E. polecki* Sensu Stricto	*E. struthionis*	*E. chattoni*
*E. polecki* s.s.	-	K_AB_ = 0.04633 (0.04598–0.04669)	K_AB_ = 0.04957 (0.04922–0.04991)
		R = 27.49 (21.87–34.24)	R = 10.12 (6.83–18.25)
*E. struthionis*	K_AB_ = 0.06116 (0.05908–0.06343)	-	K_AB_ = 0.06153 (0.06099–0.06206)
	R = 19.34 (16.26- 23.16)		R = 12.56 (8.48–20.66)
*E. chattoni*	K_AB_ = 0.09643 (0.09264–0.10023)	K_AB_ = 0.09510 (0.09147–0.09914)	-
	R = 5.22 (4.70–5.79)	R = 5.15 (4.66–5.74)	

**Table 9:** Analysis of Molecular Variance (AMOVA) for pairs of *Entamoeba* species (*Entamoeba histolytica-Entamoeba nuttalli* and *Entamoeba polecki-Entamoeba struthionis*) including sequences covering at least 75% of the alignment of the complete SSU-rRNA gene.

Source of Variation	Degrees of Freedom	Sum of Squares	Variance Components	Percentage of Variation	Fixation Index (ΦST)	*p*-Value
*Entamoeba histolytica—Entamoeba nuttalli*						
Among populations	1	102.780	6.44722	84.45	0.84452	<0.001
Within populations	31	36.796	1.18697	15.55		
*Entamoeba polecki* sensu stricto—*Entamoeba struthionis*						
Among populations	1	560.852	41.87561	96.51	0.96511	<0.001
Within populations	26	39.356	1.51371	3.49		

**Table 10:** Analysis of Molecular Variance (AMOVA) for pairs of *Entamoeba* species (*Entamoeba histolytica-Entamoeba nuttalli* and *Entamoeba polecki-Entamoeba struthionis*) including sequences covering at least 75% of the alignment of each structural domain of the SSU-rRNA molecule.

Region	Source of Variation	Degrees of Freedom	Variance Components	Percentage of Variation	Fixation Index (ΦST)	*p*-Value
***Entamoeba histolytica—Entamoeba nuttalli***						
I	Among populations	1	0.90494	53.62	0.53625	<0.001
	Within populations	58	0.78261	46.38		
II	Among	1	2.90000	92.59	0.92594	<0.001
	Within	55	0.23196	7.41		
III	Among	1	1.66178	59.45	0.59453	<0.001
	Within	130	1.13331	40.55		
IV	Among	1	0	0	0	1000
	Within	22	0	0		
***Entamoeba polecki* sensu stricto—*Entamoeba struthionis***						
I	Among	1	16.54905	97.97	0.97969	<0.001
	Within	63	0.34305	2.03		
II	Among	1	9.90788	98.36	0.95356	<0.001
	Within	39	0.48248	4.64		
III	Among	1	14.76195	96.18	0.96176	<0.001
	Within	27	0.58690	3.82		
IV	Among	1	3.87624	77.52	0.77518	<0.001
	Within	18	1.12418	22.48		

## Data Availability

The original contributions presented in the study are included in the article; further inquiries can be directed to the corresponding author.

## Supplementary Materials

The following supporting information can be downloaded at: https://www.mdpi.com/article/10.3390/microorganisms14020360/s1, File S1: Comparative analyses o in silico-predicted secondary and tertiary structures of the expansion segments ES2-ES3 of *Entamoeba histolytica* SSU rRNA; File S2: Secondary structure diagrams of the small subunit ribosomal RNA molecule of *Entamoeba* species. File S3: Partition analysis of SSU rRNA gene sequences from *Entamoeba histolytica*, *Entamoeba nuttalli* and *Entamoeba dispar* performed using ASAP. File S4: Partition analysis of SSU rRNA gene sequences from *Entamoeba polecki* sensu stricto, *Entamoeba struthionis* and *Entamoeba chattoni* performed using ASAP. File S5: Secondary structure diagrams of the SSU rRNA molecule of *Entamoeba histolytica*, *Entamoeba nuttalli*, *Entamoeba dispar*, *Entamoeba polecki* sensu stricto, *Entamoeba struthionis* and *Entamoeba chattoni* highlighting variable positions; File S6: Comparisons of the secondary structures of helices 16 to 19 of the SSU rRNA molecule in *Entamoeba chattoni* (sequences AF149912 and PP064054).

## References

[1] Collins R.A., Cruickshank R.H. (2013). The seven deadly sins of DNA barcoding. Mol. Ecol. Res..

[2] Zachos F.E., Christidis L., Gammett S.T. (2020). Mammalia species and the twofold nature of taxonomy: A comment on Taylor et al. 2019. Mammalia.

[3] Sites J.W., Marshall J. (2004). Operational Criteria for Delimiting Species. Annu. Rev. Ecol. Evol. Syst..

[4] Carstens B.C., Pelletier T.A., Reid N.M., Satler J.D. (2013). How to fail at species delimitation. Mol. Ecol..

[5] Esteban-Sánchez L., Martínez-Díaz R.A., Ponce-Gordo F. (2026). The Taxonomy of the Genus *Entamoeba* (Archamoebea: Endamoebidae): A Historical and Nomenclatural Review. Pathogens.

[6] Ponce-Gordo F., Martínez-Díaz R.A. (2010). Artículo de Revisión Taxonomía y filogenia del género Entamoeba. Una revisión histórica. Rev. Ibero-Latinoam. Parasitol..

[7] Hooshyar H., Rostamkhani P., Rezaeian M. (2015). An Annotated Checklist of the Human and Animal *Entamoeba* (Amoebida: Endamoebidae) Species—A Review Article. Iran. J. Parasitol..

[8] Stensvold C.R., Nielsen M., Baraka V., Lood R., Fuursted K., Nielsen H.V. (2021). *Entamoeba gingivalis*: Epidemiology, genetic diversity and association with oral microbiota signatures in North Eastern Tanzania. J. Oral Microbiol..

[9] Noble G.A., Noble E.R. (1952). Entamoebae in farm animals. J. Parasitol..

[10] Levine N.D. (1961). Protozoan Parasites of Domestic Animals and of Man.

[11] Elsheikha H.M., Regan C.S., Clark C.G. (2018). Novel *Entamoeba* findings in nonhuman primates. Trends Parasitol..

[12] Diamond L.S., Clark C.G. (1993). A redescription of *Entamoeba histolytica* Schaudinn, 1903 (Emend Walker, 1911) separating it from *Entamoeba dispar* Brumpt, 1925. J. Eukaryot. Microbiol..

[13] Clark C.G., Kaffashian F., Tawari B., Windsor J.J., Twigg-Flesner A., Davies-Morel M.C.G., Blessmann J., Ebert F., Peschel B., Van A.L. (2006). New insights into the phylogeny of *Entamoeba* species provided by analysis of four new small-subunit rRNA genes. Int. J. Syst. Evol. Microbiol..

[14] Tachibana H., Yanagi T., Pandey K., Cheng X.J., Kobayashi S., Sherchand J.B., Kanbara H. (2007). An *Entamoeba* sp. strain isolated from rhesus monkey is virulent but genetically different from *Entamoeba histolytica*. Mol. Biochem. Parasitol..

[15] Clark C.G., Diamond L.S. (1997). Intraspecific variation and phylogenetic relationships in the genus *Entamoeba* as revealed by riboprinting. J. Eukaryot. Microbiol..

[16] Ponce-Gordo F., Martínez-Díaz R.A., Herrera S. (2004). *Entamoeba struthionis* n.sp. (Sarcomastigophora: Endamoebidae) from ostriches (*Struthio camelus*). Vet. Parasitol..

[17] Royer T.L., Gilchrist C., Kabir M., Arju T., Ralston K.S., Haque R., Clark C.G., Petri W.A. (2012). *Entamoeba bangladeshi* nov. sp., Bangladesh. Emerg. Infect. Dis..

[18] Shiratori T., Ishida K. (2016). *Entamoeba marina* n. sp.; a new species of *Entamoeba* isolated from tidal flat sediment of Iriomote Island, Okinawa, Japan. J. Eukaryot. Microbiol..

[19] Jinatham V., Popluechai S., Clark C.G., Gentekaki E. (2019). *Entamoeba chiangraiensis* n. sp. (Amoebozoa: Entamoebidae) isolated from the gut of Asian swamp eel (*Monopterus albus*) in northern Thailand. Parasitology.

[20] Stensvold C.R., Lebbad M., Victory E.L., Verweij J.J., Tannich E., Alfellani M., Legarraga P., Clark C.G. (2011). Increased Sampling Reveals Novel Lineages of *Entamoeba*: Consequences of Genetic Diversity and Host Specificity for Taxonomy and Molecular Detection. Protist.

[21] Jacob A.S., Busby E.J., Levy A.D., Komm N., Clark C.G. (2016). Expanding the *Entamoeba* Universe: New Hosts Yield Novel Ribosomal Lineages. J. Eukaryot. Microbiol..

[22] Esteban-Sánchez L., García-Rodríguez J.J., Ponce-Gordo F. (2025). Unusual Findings of Human-Associated Tetranucleate *Entamoeba* Species in Captive Wild Animals. Animals.

[23] Wilson I.W., Weedall G.D., Lorenzi H., Howcroft T., Hon C.C., Deloger M., Guillén N., Paterson S., Clark C.G., Hall N. (2019). Genetic Diversity and Gene Family Expansions in Members of the Genus *Entamoeba*. Genome Biol. Evol..

[24] Novati S., Sironi M., Granata S., Bruno A., Gatti S., Scaglia M., Bandi C. (1996). Direct sequencing of the PCR amplified SSU rRNA gene of *Entamoeba dispar* and the design of primers for rapid differentiation from *Entamoeba histolytica*. Parasitology.

[25] Silberman J.D., Clark C.G., Diamond L.S., Sogin M.L. (1999). Phylogeny of the genera *Entamoeba* and *Endolimax* as deduced from small-subunit ribosomal RNA sequences. Mol. Biol. Evol..

[26] Ramachandran S., Bhattacharya A., Bhattacharya S. (1993). Nucleotide sequence analysis of the rRNA transcription unit of a pathogenic *Entamoeba histolytica* strain HM-1:IMSS. Nucleic Acids Res..

[27] Kobayashi S., Suzuki J., Takeuchi T. (2009). Establishment of a continuous culture system for *Entamoeba muris* and analysis of the small subunit rRNA gene. Parasite.

[28] Stensvold C.R., Lebbad M., Clark C.G. (2010). Genetic characterisation of uninucleated cyst-producing *Entamoeba* spp. from ruminants. Int. J. Parasitol..

[29] Yamamoto A., Kikuta N., Hashimoto T., Oyaizu H., Goto N. (1995). Nucleotide sequence of the SrRNA gene of *Entamoeba gingivalis*: Applications for construction of a species-specific DNA probe and phylogenetic analysis. Microbiol. Immunol..

[30] Edgar R.C. (2004). MUSCLE: Multiple sequence alignment with high accuracy and high throughput. Nucleic Acids Res..

[31] Kumar S., Stecher G., Li M., Knyaz C., Tamura K. (2018). MEGA X: Molecular Evolucionary Genetics Analysis across Computing Platforms. Mol. Biol. Evol..

[32] Lu X.J., Bussemaker H.J., Olson W.K. (2015). DSSR: An integrated software tool for dissecting the spatial structure of RNA. Nucleic Acids Res..

[33] Sharma S., Mishra S., Gourinath S., Kaushal P.S. (2025). Cryo-EM structure of ribosome from pathogenic protozoa *Entamoeba histolytica* reveals unique features of its architecture. Nat. Commun..

[34] Abramson J., Adler J., Dunger J., Evans R., Green T., Pritzel A., Ronneberger O., Willmore L., Ballard A.J., Bambrick J. (2024). Accurate structure prediction of biomolecular interactions with AlphaFold 3. Nature.

[35] Lorenz R., Bernhart S.H., zu Siederdissen H., Tafer H., Flamm C., Stadler P.F., Hofacker I.L. (2011). ViennaRNA Package 2.0. Algorithms Mol. Biol..

[36] Seibel P.N., Müller T., Dandekar T., Wolf M. (2008). Synchronous visual analysis and editing of RNA sequence and secondary structure alignments using 4SALE BMC. Res. Notes.

[37] Ankenbrand M.J., Keller A., Wolf M., Schultz J., Förster F. (2015). ITS2 database V: Twice as much. Mol. Biol. Evol..

[38] De Rijk P., Wuyts J., De Wachter R. (2003). RnaViz2: An improved representation of RNA secondary structure. Bioinformatics.

[39] Nei M., Li W.H. (1979). Mathematical model for studying genetic variation in terms of restriction endonucleases. Proc. Natl. Acad. Sci. USA.

[40] Python Software Foundation Python 3.14.2 Documentation 2025. https://docs.python.org/3/index.html.

[41] Cock P.J.A., Antao T., Chang J.T., Chapman B.A., Cox C.J., Dalke A., Friedberg I., Hamelryck T., Kauff F., Wilczynski B. (2009). Biopython: Freely available Python tools for computational molecular biology and bioinformatics. Bioinformatics.

[42] R Core Team (2021). R: A Language and Environment for Statistical Computing.

[43] Romero M., Cerritos R., Ximenez C. (2016). Horizontal Gene Transfers from Bacteria to *Entamoeba* Complex: A Strategy for Dating Events along Species Divergence. J. Parasitol. Res..

[44] Wickham H., Averick M., Bryan J., Chang W., McGowan L.D., François R., Grolemund G., Hayes A., Henry L., Hester J. (2019). Welcome to the tidyverse. J. Open Source Softw..

[45] Kassambara A. (2023). rstatix: Pipe-Friendly Framework for Basic Statistical Tests. R Package Version 0.7.2. https://rpkgs.datanovia.com/rstatix/.

[46] Seshan V.E., Whiting K. (2023). Clinfun: Clinical Trial Design and Data Analysis Functions. https://cran.r-project.org/web/packages/clinfun/index.html.

[47] Birky C.W., Wolf C., Maughan H., Herbertson L., Henry E. (2005). Speciation and selection without sex. Hydrobiologia.

[48] Petrov A.S., Bernier C.R., Gulen B., Waterbury C.C., Hershkovits E., Hsiao C., Harvey S.C., Hud N.V., Fox G.E., Wartell R.M. (2014). Secondary structures of rRNAs from all three domains of life. PLoS ONE.

[49] Excoffier L., Lischer H.E.L. (2010). Arlequin suite ver 3.5: A new series of programs to perform population genetics analyses under Linux and Windows. Mol. Ecol. Res..

[50] Puillandre N., Brouillet S., Achaz G. (2021). ASAP: Assemble species by automatic partitioning. Mol. Ecol. Resour..

[51] Ponce-Gordo F., Martínez-Díaz R.A. (2007). On the identification of some *Entamoeba* species. Comments on a recent paper. Int. J. Syst. Evol. Microbiol..

[52] Mayr E. (1982). The Growth of Biological Thought: Diversity, Evolution and Inheritance.

[53] Weedal G.D., Clark C.G., Koldkjaer P., Kay S., Bruchhaus I., Tannich E., Paterson S., Hall N. (2012). Genomic diversity of the human intestinal parasite *Entamoeba histolytica*. Genome Biol..

[54] Weedal G.D., Hall N. (2015). Sexual reproduction and genetic exchange in parasitic protists. Prasitology.

[55] Simspon G.G. (1951). The species concept. Evolution.

[56] De Queiroz K. (2007). Species concepts and species delimitation. Syst. Biol..

[57] Seifert B. (2014). A pragmatic species concept applicable to all eukaryotic organisms independent from their mode of reproduction or evolutionary history. Soil Org..

[58] Miralles A., Puillandre N., Vences M., DeSalle R. (2024). DNA barcoding in species delimitation: From genetic distances to integrative taxonomy. DNA Barcoding: Methods and Protocols. Methods in Molecular Biology.

[59] Barraclough T.G., Birky C.W., Burt A. (2003). Diversification in sexual and asexual organisms. Evolution.

[60] Birky C.W., Adams J., Gemmel M., Perry J. (2010). Using Population Genetic Theory and DNA Sequences for Species Detection and Identification in Asexual Organisms. PLoS ONE.

[61] Bickford D., Lohman D.J., Sodhi N.S., Ng P.K., Meier R., Winker K., Ingram K.K., Das I. (2007). Cryptic species as a window on diversity and conservation. Trends Ecol. Evol..

[62] Garcia G., Ramos F., Maldonado J., Fernandez A., Yanez J., Hernandez L., Yáñez J., Gaytán P. (2018). Prevalence of two *Entamoeba gingivalis* ST1 and ST2-kamaktli subtypes in the human oral cavity under various conditions. Parasitol. Res..

[63] Dayrat B. (2005). Towards integrative taxonomy. Biol. J. Linnean Soc..

[64] Goldstein P.Z., DeSalle R. (2010). Integrating DNA barcode data and taxonomic practice: Determination, discovery, and description. Bioassays.

[65] Padial J.M., Miralles A., De la Riva I., Vences M. (2010). The integrative future of taxonomy. Front. Zool..

[66] Warren A., Patterson D.J., Dunthorn M., Clamp J.C., Achilles-Day U.E.M., Aescht E., Al-Farraj S.A., AlQuraishy S., Al-Rasheid K., Carr M. (2017). Beyond the “Code”: A guide to the description and documentation of biodiversity in ciliated protist (Alveolata, Ciliophora). J. Eukaryot. Microbiol..

[67] Leliaert F., Verbruggen H., Vanormelingen P., Steen F., López-Bautista J.M., Zuccarello G.C., de Clerk O. (2014). DNA-based species delimitation in algae. Eur. J. Phycol..

[68] Birky C.W. (2013). Species detection and identification in sexual organisms using population genetic theory and DNA sequences. PLoS ONE.

[69] DeSalle R., Egan M.G., Siddall M. (2005). The unholy trinity: Taxonomy, species delimitation and DNA barcoding. Philos. Trans. R. Soc. B Biol. Sci..

[70] Ebach M.C., Holdrege C. (2005). DNA barcoding is no substitute for taxonomy. Nature.

[71] International Comission on Zoological Nomenclature (1999). International Code of Zoological Nomenclature.

[72] International Commission on Zoological Nomenclature (2017). Declaration 45—Addition of Recommendations to Article 73 and of the term “specimen, preserved” to the Glossary. Bull. Zool. Nomenclat..

[73] International Comission on Zoological Nomenclature Frequently Asked Questions—Can DNA Be a Type Specimen?. https://www.iczn.org/outreach/faqs.

[74] Tedersoo L., Geisen S., Chang Y., Nilsson R.H. (2026). Toward DNA-based taxonomy of prokaryotes and microeukaryotes. Trends Genet..

[75] Rheindt F.E., Bouchard P., Pyle R.L., Welter-Schultes F., Aescht E., Ahyong S.T., Ballerio A., Bourgoin T.Y., Ceriaco L.M.P., Dmitriev D. (2023). Tightening the requirements for species diagnoses would help integrate DNA-based descriptions in taxonomic practice. PLoS Biol..

[76] Evenhuis N.L. (2008). A compendium of Zoological Type Nomenclature: A Reference Source Bishop Museum Technical Report 41.

[77] Liao D. (1999). Concerted evolution: Molecular mechanism and biological implications. Am. J. Hum. Genet..

[78] Haig D. (2021). Concerted evolution of ribosomal DNA: Somatic peace amid germinal strife. BioEssays.

[79] Eickbush T.H., Eickbush D.G. (2007). Finely Orchestrated Movements: Evolution of the Ribosomal RNA Genes. Genetics.

[80] Paloi S., Luangsa-ard J.J., Mhuantong W., Standler M., Kobmoo N. (2022). Intragenomic variation in nuclear ribosomal markers and its implication in species delimitation, identification and barcoding in fungi. Fungal Biol. Rev..

[81] Wang W., Zhang X., Garcia S., Leitch A.R., Kovařík A. (2023). Intragenomic rDNA variation—The product of concerted evolution, mutation, or something in between?. Heredity.

[82] Sultanov D., Hochwagen A. (2022). Varying strength of selection contributes to the intragenomic diversity of rRNA genes. Nat. Commun..

[83] Xue S., Barna M. (2012). Specialized ribosomes: A new frontier in gene regulation and organismal biology. Nat. Rev. Mol. Cell Biol..

[84] Genuth N.R., Barna M. (2018). The Discovery of Ribosome Heterogeneity and Its Implications for Gene Regulation and Organismal Life. Mol. Cell.

[85] Welfer G.A., Brady R.A., Natchiar S.K., Watson Z.L., Rundlet E.J., Alejo J.L., Singh A.P., Mishra N.K., Altman R.B., Blanchard S.C. (2025). Impacts of ribosomal RNA sequence variation on gene expression and phenotype. Philos. Trans. R. Soc. B.

[86] Zuriaga M.A., Mas-Coma S., Bargues M.D. (2015). A nuclear ribosomal DNA pseudogene in triatomines opens a new research field of fundamental and applied implications in Chagas disease. Mem. Inst. Oswaldo Cruz.

[87] Robicheau B.M., Susko E., Harrigan A.M., Snyder M. (2017). Ribosomal RNA genes contribute to the formation of pseudogenes and junk DNA in the human genome. Genome Biol. Evol..

[88] Bhattacharya S., Bhattacharya A., Diamond L.S., Soldo A.T. (1989). Circular DNA of *Entamoeba histolytica* encodes ribosomal RNA. J. Protozool..

[89] Wesche P.L., Gaffney D.J., Keightley P.D. (2004). DNA sequence error rates in Genbak records estimated using the mouse genome as reference. DNA Seq..

[90] Nilsson R.H., Ryberg M., Kristiansson E., Abarenkov K., Larsson K.H., Kõljalg U. (2006). Taxonomic Reliability of DNA Sequences in Public Sequence Databases: A Fungal Perspective. PLoS ONE.

[91] Lin Y.H., Chang B.C., Chiang P.W., Tang S.L. (2008). Questionable 16S ribosomal RNA gene annotations are frequent in completed microbial genomes. Gene.

[92] Schnoes A.M., Brownm S.D., Dodevski I., Babbitt P.C. (2009). Annotation Error in Public Databases: Misannotation of Molecular Function in Enzyme Superfamilies. PLoS Comput. Biol..

[93] Edgar R. (2018). Taxonomy annotation and guide tree errors in 16S rRNA databases. PeerJ.

[94] Locatelli N.S., McIntyre P.B., Therkildsen N.O., Baetscher D.S. (2020). GenBank’s reliability is uncertain for biodiversity researchers seeking species-level assignment for eDNA. Proc. Natl. Acad. Sci. USA.

[95] Richterich P. (1998). Estimation of errors in “raw” DNA sequences: A validation study. Genome Res..

[96] Schirmer M., Ijaz U.Z., D’Amore R., Hall N., Sloan W.T., Quince C. (2015). Insight into biases and sequencing errors for amplicon sequencing with the Illumina MiSeq platform. Nucleic Acids Res..

[97] Ma X., Shao Y., Tian L., Flasch D.A., Mulder H.L., Edmonson M.N., Liu Y., Chen X., Newman S., Nakitandwe J. (2019). Analysis of error profiles in deep next-generation sequencing data. Genome Biol..

[98] Alachiotis N., Vogiatzi E., Pavlidis P., Stamatakis A. (2013). ChromatoGate: A Tool for Detecting Base Mis-Calls in Multiple Sequence Alignments by Semi-Automatic Chromatogram Inspection. Comput. Struct. Biotechnol. J..

[99] Elyazghi Z., Loubna E., Sadki K., Fouzia R. (2017). ABI Base Recall: Automatic correction and ends trimming of DNA sequences. IEEE Trans. NanoBiosci..

[100] Sipos R., Székely A.J., Palatinszky M., Révész S., Márialigeti K., Nikolausz M. (2007). Effect of primer mismatch, annealing temperature and PCR cycle number on 16S rRNA gene-targetting bacterial community analysis. FEMS Microbiol. Ecol..

[101] Kjer K.M. (1995). Use of rRNA secondary structure in phylogenetic studies to identify homologous positions: An example of alignment and data presentation from the frogs. Mol. Phylogenet. Evol..

[102] Gillespie J.J., Yoder M.J., Wharton R.A. (2005). Predicted secondary structure for 28S and 18S rRNA from Ichneumonoidea (Insecta: Hymenoptera: Apocrita): Impact on sequence alignment and phylogeny estimation. J. Mol. Evol..

[103] Kjer K.M., Gillespie J.J., Ober K.A. (2007). Opinions on multiple sequence alignment, and an empirical comparison of repeatability and accuracy between POY and structural alignment. Syst. Biol..

[104] Voigt O., Erpenbeck D., Wörheide G. (2008). Molecular evolution of rDNA in early diverging Metazoa: First comparative analysis and phylogenetic application of complete SSU rRNA secondary structures in Porifera. BMC Evol. Biol..

[105] Keller A., Förster F., Müller T., Dandekar T., Schultz J., Wolf M. (2010). Including RNA secondary structures improves accuracy and robustness in reconstruction of phylogenetic trees. Biol. Direct..

[106] Alfonso S., Martínez-Díaz R.A., Ponce-Gordo F. (2012). Estructura secundaria y mapa de variabilidad de la subunidad pequeña del ARNr de *Entamoeba*. Posibles implicaciones para la taxonomía del género. Rev. Ibero-Latinoam. Parasitol..

[107] Ohta T. (1973). Slightly deleterious mutant substitutions in evolution. Nature.

[108] Ohta T. (1992). The nearly neutral theory of molecular evolution. Annu. Rev. Ecol. Evol. Syst..

[109] Smit S., Widmann J., Knight R. (2007). Evolutionary rates vary among rRNA structural elements. Nucleic Acids Res..

[110] Gerbi S.A. (1986). The evolution of eukaryotic ribosomal DNA. Biosystems.

[111] Gerbi S.A., Zimmermann R.A., Dahlberg A.E. (1996). Expansion segments: Regions of variable size that interrupt the universal core secondary structure of ribosomal RNA. Ribosomal RNA—Structure, Evolution, Processing, and Function in Protein Synthesis.

[112] Nelles L., Fang B.-L., Volckaert G., Vandenberghe A., De Wachter R. (1984). Nucleotide sequence of a crustacean 18S RNA gene and secondary structure of eukaryotic small subunit ribosomal RNAs. Nucleic Acids Res..

[113] Neefs J.M., Van de Peer Y., De Rijk P., Chapelle S., De Wachter R. (1993). Compilation of small ribosomal subunit RNA structures. Nucleic Acids Res..

[114] Pawlowski J., Audic S., Adl S., Bass D., Belbahri L., Berney C., Bowser S.S., Cepicka I., Decelle J., Dunthorn M. (2012). CBOL Protist Working Group: Barcoding eukaryotic richness beyond the animal, plant, and fungal kingdoms. PLoS Biol..

[115] Zhang Y., Zhao Y.J., Wang Q., Tang F.H. (2015). New Comparative Analysis Based on the Secondary Structure of SSU-rRNA Gene Reveals the Evolutionary Trend and the Family-Genus Characters of Mobilida (Ciliophora, Peritrichia). Curr. Microbiol..

[116] Du Y.H., Zhao Y.J., Tang F.H. (2018). A New Molecular Approach Based on the Secondary Structure of Ribosomal RNA for Phylogenetic Analysis of Mobilid Ciliates. Curr. Microbiol..

[117] De Luca D., Piredda R., Sarno D., Kooistra W.H.C.F. (2021). Resolving cryptic species complexes in marine protists: Phylogenetic haplotype networks meet global DNA metabarcoding datasets. ISME J..

[118] Wardani R.K., Ahsan R., Shin M.K. (2025). Evolutionary patterns of the SSU rRNA (V4 region) secondary structure in genus *Euplotes* (Ciliophora, Spirotrichea): Insights into cryptic species and primitive traits. PeerJ.

[119] Coleman A.W. (2003). ITS2 is a double-edged tool for eukaryote evolutionary comparisons. Trends Genet..

[120] Coleman A.W. (2007). Pan-eukaryote ITS2 homologies revealed by RNA secondary structure. Nucleic Acids Res..

[121] Coleman A.W. (2009). Is there a molecular key to the level of “biological species” in eukaryotes? A DNA guide. Mol. Phylogenet. Evol..

[122] Müller T., Philippi N., Dandekar T., Schultz J., Wolf M. (2007). Distinguishing species. RNA.

[123] Ahvenniemi P., Wolf M., Lehtonen M.J., Wilson P., German-Kinnari M., Valkonen J.P.T. (2009). Evolutionary diversification indicated by compensatory base changes in ITS2 secondary structures in a complex fungal species, *Rhizoctonia solani*. J. Mol. Evol..

[124] Schill R.O., Förster F., Dandekar T., Wolf M. (2010). Using compensatory base change analysis of internal transcribed spacer 2 secondary structures to identify three new species in *Paramacrobiotus* (Tardigrada). Org. Divers. Evol..

[125] Wolf M., Chen S., Song J., Ankenbrand M., Müller T. (2013). Compensatory base changes in ITS2 secondary structures correlate with the Biological Species Concept despite intragenomic variability in ITS2 sequences—A proof of concept. PLoS ONE.

[126] Tachibana H., Yanaghi T., Feng M., Bandara K.B.A.T., Kobayashi S., Cheng X., Hirayama K., Rajapakse R.P.V.J. (2016). Isolation and molecular characterization of *Entamoeba nuttalli* strains showing novel isoenzyme patterns from wild Toque Macaques in Sri Lanka. J. Eukaryot. Microbiol..

[127] Swellengrebel N.H. (1914). Dierlijke entamoeben uit Deli. Geneesk. Tijdschr. Ned. Indië.

[128] Verweij J.J., Polderman A.M., Clark C.G. (2001). Genetic variation among human isolates of uninucleated cyst-producing *Entamoeba* secies. J. Clin. Microbiol..

[129] Clark C.G., Windsor J.J., Tannich E. (2007). On the identification of some *Entamoeba* species—Response to Ponce-Gordo and Martínez-Díaz. Int. J. Syst. Microbiol. Evol..

[130] Clark C.G., Stensvold C.R., Nozaki T., Bhattacharya A. (2015). The Continuously Expanding Universe of *Entamoeba*. Amebiasis.

[131] Stenvold C.R., Winiecka-Krusnell J., Lier T., Lebbad M. (2018). Evaluation of a PCR method for detection of *Entamoeba polecki*, with an overview of its molecular epidemiology. J. Clin. Microbiol..

[132] Jirků-Pomajbíková K., Čepička I., Kalousová B., Jirků M., Stewart F., Levecke B., Modrý D., Piel A.K., Petrželková K.J. (2016). Molecular identification of *Entamoeba* species in savanna woodland chimpanzees (*Pan troglodytes schweinfurtyhii*). Parasitology.

[133] Hirashima Y., Manchanayake T., Yano T., Kitahara S., Koreeda T., Kamimura S., Sasai K., Matsubayashi M., Shibahara T. (2017). Development of molecular diagnostic protocols for detecting three types of *Entamoeba* from diarrheal and asymptomatic pigs and environmental moist soils. Parasitol. Res..

[134] Villanueva-García C., Gordillo-Chávez E.J., Baños-Ojeda C., Rendón-Franco E., Muñoz-García C.I., Carrero J.C., Córdoba-Aguilar A., Maravilla P., Galian J., Martínez-Hernández F. (2017). New *Entamoeba* group in howler monkeys (*Alouatta* spp.) associated with parasites of reptiles. Parasitol. Res..

[135] Wang Y., Zeng Y., Wu Y., Lu F., Hou X., Shao J., Zhang T., Shao C. (2024). Molecular characterization and zoonotic potential of *Entamoeba* spp., *Enterocytozoon bieneusi* and *Blastocystis* from captive wild animals in northwest China. BMC Vet. Res..

[136] Tachibana H., Yanagi T., Lama C., Pandey K., Feng M., Kobayashi S., Sherchand J.B. (2013). Prevalence of *Entamoeba nuttalli* infection in wild rhesus macaques in Nepal and characterization of the parasite isolates. Parasitol. Int..

[137] Matsubayashi M., Kanamori K., Sadahiro M., Tokoro M., Abe N., Haritani M., Shibahara T. (2015). First molecular identification of *Entamoeba polecki* in a piglet in Japan and implications for aggravation of ileitis by coinfection with *Lawsonia intracellularis*. Parasitol. Res..

[138] Sylvain P.N., Kaur U., Goyal K., Sehgal R., Paul M.F. (2015). Molecular differentiation of *Entamoeba* spp. Isolated from Cameroonian human immunodeficiency virus (HIV) infected and uninfected patient. J. Parasitol. Vector Biol..

[139] Symeonidou I., Diakou A., Papadopoulos E., Ponce-Gordo F. (2019). Endoparasitism of Greek ostriches: First report of *Entamoeba struthionis* and *Balantioides coli*. Vet. Parasitol. Reg. Stud. Rep..

[140] Tuda J., Feng M., Imada M., Kobayashi S., Cheng X., Tachibana H. (2016). Identification of *Entamoeba polecki* with unique 18S rRNA gene sequences from Celebes crested macaques and pigs in Tangkoko Nature Rserve, North Sulawesi, Indonesia. J. Eukaryot. Microbiol..

[141] Stensvold C.R., Berg R.P.K.D., Maloney J.G., Molokin A., Santin M. (2023). Molecular characterization of *Blastocystis* and *Entamoeba* of muskoxen and sheep in Greenland. Int. J. Parasitol..

