# Supplementary material for: Assessing Species Delimitation in Entamoeba (Amoebozoa: Endamoebidae) Using the Small Subunit rRNA Gene: Its Application to the Entamoeba polecki Complex
Source: Microorganisms. 2026 Feb 3;14(2):360. doi: 10.3390/microorganisms14020360 (PMC12942770; doi:10.3390/microorganisms14020360)
Supplement: Supplementary file 1 [file microorganisms-14-00360-s001.zip › Supplementary File 1.pdf]

Supplementary File 1. Comparative analyses of in silico-predicted secondary and tertiary structures of the expansion segments ES2-ES3 of *Entamoeba histolytica* SSU-rRNA.

The partial 3D and 2D models derived from the experimentally determined structure of SSU-rRNA of *E. histolytica* (Protein Data Bank entry 9V24) [1](Figure S1 - A, B) were compared with those generated in silico in the AlphaFold3 server (https://alphafoldserver.com/welcome) [2] and the RNAFold utility [3] implemented in the RNAfold WebServer (http://rna.tbi.univie.ac.at/cgi-bin/RNAWebSuite/RNAfold.cgi) for the *E. histolytica* X65163 sequence (Figure S1 - C, D). The secondary structure models derived from the AlphaFold models were obtained by using DSSR version 2.5.0-2025 [4]; these models and those from the RNAfold WebServer were compared and edited in 4SALE [5].

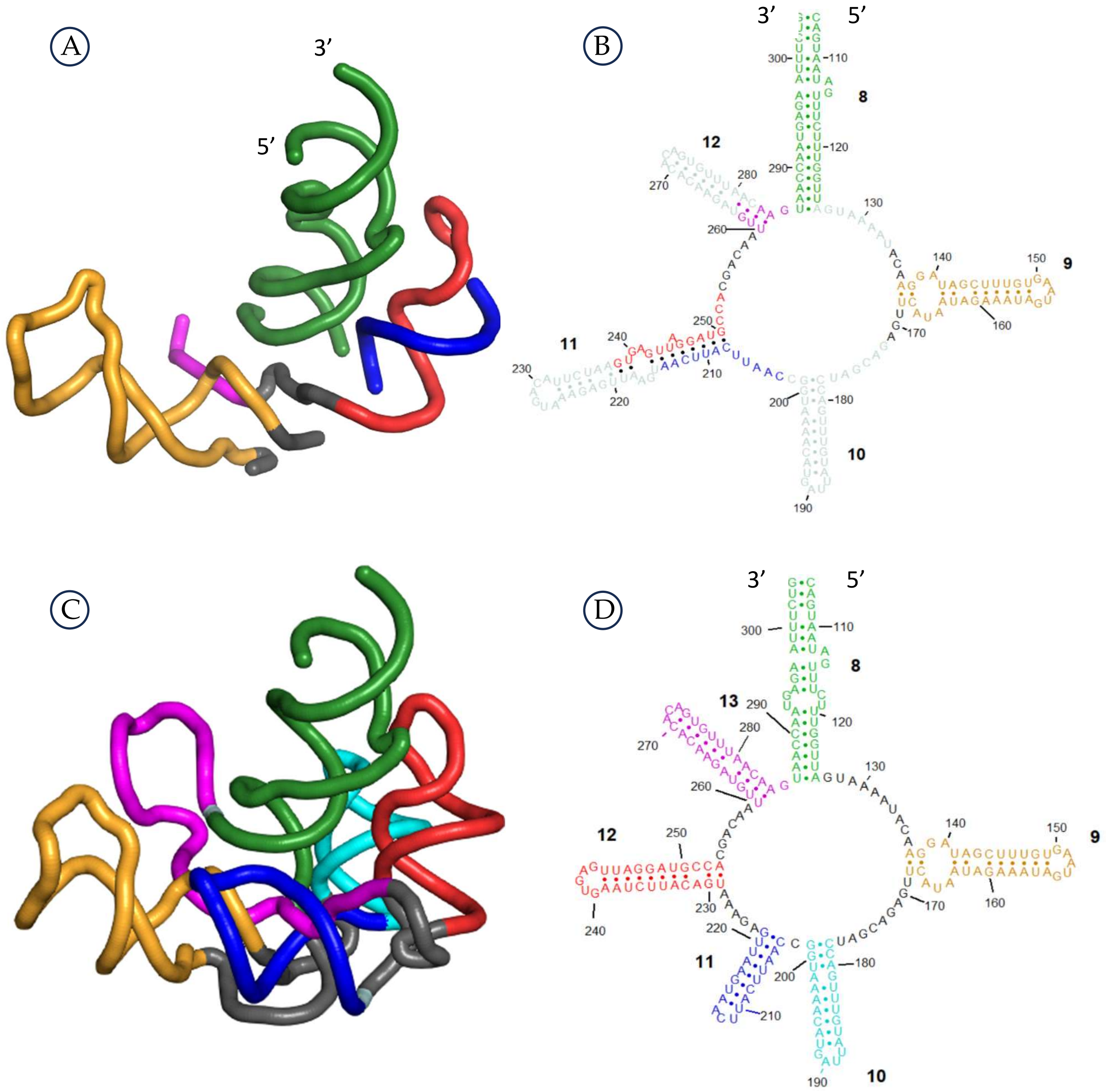

Figure S1. Secondary and tertiary models of the expansion segments 2 and 3 (ES2 and ES3) of *Entamoeba histolytica*. A) Partial 3D model experimentally determined (PDB entry 9V24). B) Secondary structure model derived from the partially determined 3D model in A). C) Three-dimension model predicted with the AlphaFold3 server for the *E. histolytica* X65163 sequence. D) Consensus 2D model derived from the 3D model determined in C) and from the RNAfold webserver. Base positions in B) and D) are given according to the *E. histolytica* X65163 sequence. The same colours are used for the helices in all models. Fragments not available in the 3D model in A) are coloured pale gray in B).

For each model, thermodynamic stability was assessed with the RNAfold version 2.7.0 of the ViennaRNA package (Linux version). The Infernal 1.1.5 package [6] was used to build and align covariance models for each 2D structure; the models were calibrated ("cmcalibrate" command) and searches and alignments ("cmsearch" and "cmalign" commands) were performed using the SSU-rRNA sequences of other *Entamoeba* species (Table 1 in the main text). The presence and significance of covarying pairs in the helices of the two model options were used as evidence supporting evolutionary conservation of specific helices. Covariation analysis was performed with R-scape version 2.6.4 [7]. The results of the analyses are shown in Table S1.

Table S1. Comparative evaluation of alternative secondary-structure models for the region between positions 203–251 of *Entamoeba histolytica* SSU-rRNA (GenBank accession number X65163).

	Model		
Parameter	single helix	two helices	Interpretation / comment
**A. Folding (RNAfold - ViennaRNA v2.6.4)**			
Number of paired positions	15	18	Pairing potential
Minimum free energy (kcal·mol^{-1})	-8.00	-14.00	Lower values indicate higher thermodynamic stability
Ensemble diversity	1.25	1.52	Lower values indicate a more defined structural ensemble
Mean base-pairing probability (p⟩	0.95	0.97	Higher values indicate more stable and probable base pairs
**B. Covariation analysis (R-scape v2.6.4)**			
Total paired positions	15	18	
Covarying pairs ($p < 0.05$)	0	6	Covariation detected only in the two helices hypothesis
Range of *p*-values	---	$4.88e^{-8}$ - $1.26e^{-5}$	Lower values indicate stronger evidence for compensatory substitutions
Mean analysis power	0.23	0.31	Higher values indicate better detection of true covariation
**C. Comparative alignment (Infernal 1.1.5; 21 additional *Entamoeba* spp. sequences)**			
Number of significant hits (E < 0.01)	5	12	Higher number indicates better transferability across related species
Mean bit score (cmsearch)	42.2	34.8	Higher scores indicate better model fit
Mean bit score ± standard deviation (cmalign)	1.47 ± 21.13	10.97 ± 23.54	Higher scores indicate a more consistent sequence-structure compatibility
Alignment quality across *Entamoeba* spp.	Poor, many gaps required	Good, coherent stem–loop boundaries	

The one-helix option was suggested by Sharma and coworkers [1] and is compatible with the truncated 3D model of *E. histolytica*, however it is hardly transferable to sequences of other species. Integrating the outputs of different analyses, the results robustly support that the fragment under analysis forms two independent helices rather than a single continuous stem.

CITED REFERENCES

1. Sharma, S., Mishra, S., Gourinath, S. Kaushal, P.S. Cryo-EM structure of ribosome from pathogenic protozoa *Entamoeba histolytica* reveals unique features of its architecture. *Nat Commun* **2025**, *16*, 7758. Doi: 10.1038/s41467-025-62767-x

2. Abramson, J.; Adler, J.; Dunger, J.; Evans, R.; Green, T.; Pritzel, A.; Ronneberger, O.; Willmore, L.; Ballard, A.J.; Bambrick, J.; *et al.* Accurate structure prediction of biomolecular interactions with AlphaFold 3. *Nature* 2024, 630, 493–500. Doi: 10.1038/s41586-024-07487-w

3. Lorenz, R.; Bernhart, S.H.; zu Siederdissen, H.; Tafer, H.; Flamm, C.; Stadler, P.F.; Hofacker, I.L. ViennaRNA Package 2.0. Algorithms Mol. Biol. 2011, 6, 26. Doi; 10.1186/1748-7188-6-26

4. Lu, X.J.; Bussemaker, H.J.; Olson, W.K. DSSR: an integrated software tool for dissecting the spatial structure of RNA. Nucleic Acids Res. 2015, 43, e142. Doi: 10.1093/nar/gkv716

5. Seibel P.N., Müller T., Dandekar T., Wolf M. Synchronous visual analysis and editing of RNA sequence and secondary structure alignments using 4SALE *BMC Res. Notes* **2008**, *1*, 91. Doi: 10.1186/1756-0500-1-91

6. Nawrocki, E.P., Eddy, S.R. Infernal 1.1: 100-fold faster RNA homology searches. *Bioinformatics* **2013**, *29*, 2933-2935. Doi: 10.1093/bioinformatics/btt509

7. Rivas, E. RNA covariation at helix-level resolution for the identification of evolutionarily conserved RNA structure. *PLOS Comput. Biol.* **2023**, *19*, e111262. Doi: 10.1371/journal.pcbi.1011262.
