# Supplementary material for: Assessing Species Delimitation in Entamoeba (Amoebozoa: Endamoebidae) Using the Small Subunit rRNA Gene: Its Application to the Entamoeba polecki Complex
Source: Microorganisms. 2026 Feb 3;14(2):360. doi: 10.3390/microorganisms14020360 (PMC12942770; doi:10.3390/microorganisms14020360)
Supplement: Supplementary file 1 [file microorganisms-14-00360-s001.zip › Supplementary File 2.pdf]

Supplementary File 2. Secondary structure diagrams of the small subunit ribosomal RNA molecule of *Entamoeba* species.

The diagrams represent the SSU-rRNA secondary structure for the reference sequences selected for each *Entamoeba* species. The diagrams are ordered alphabetically according to the species' morphological group: four-nucleated, eight-nucleated, or one-nucleated mature cyst-forming species, and non-cyst forming species.

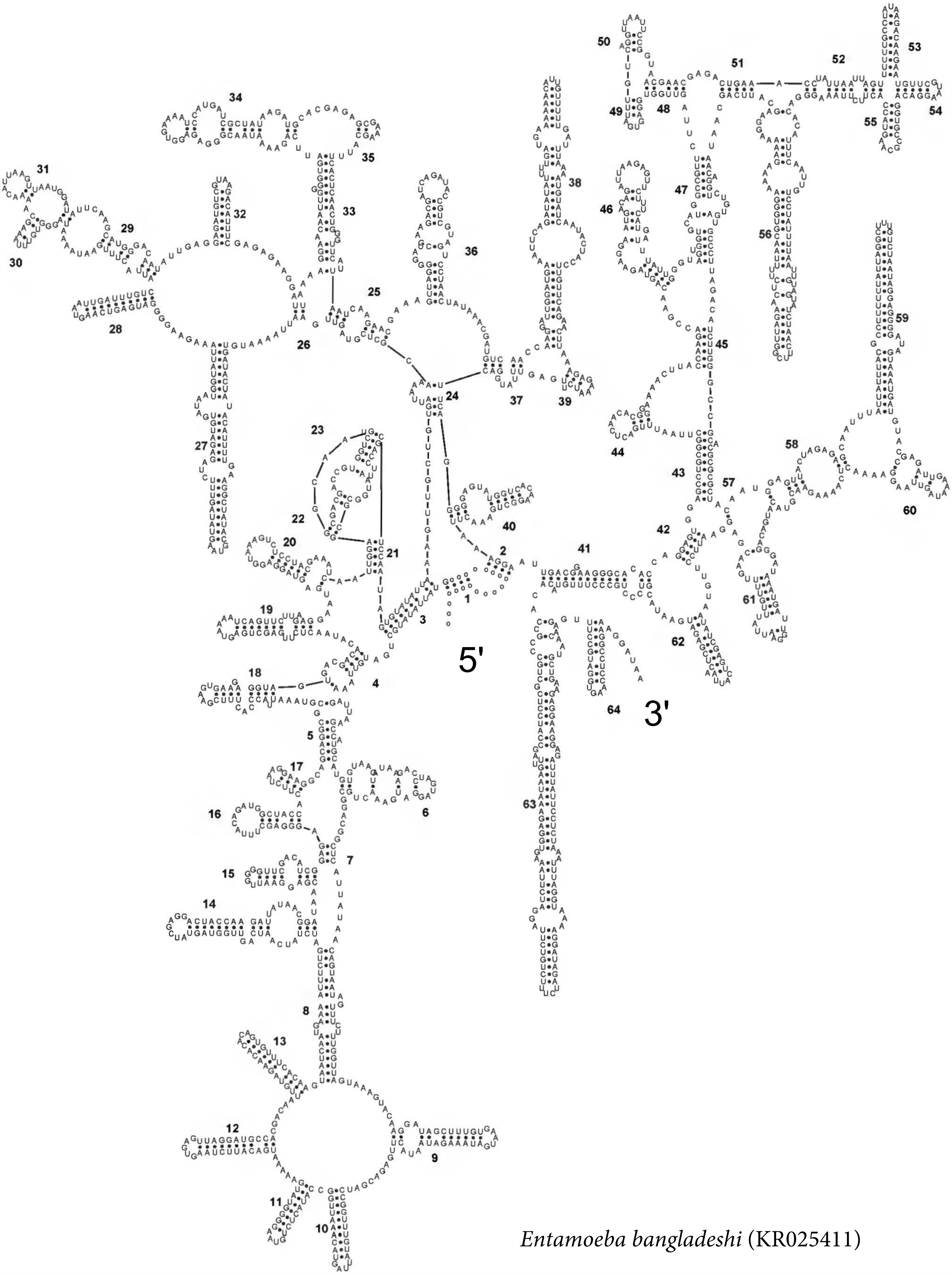

Entamoeba bangladeshi (KR025411)

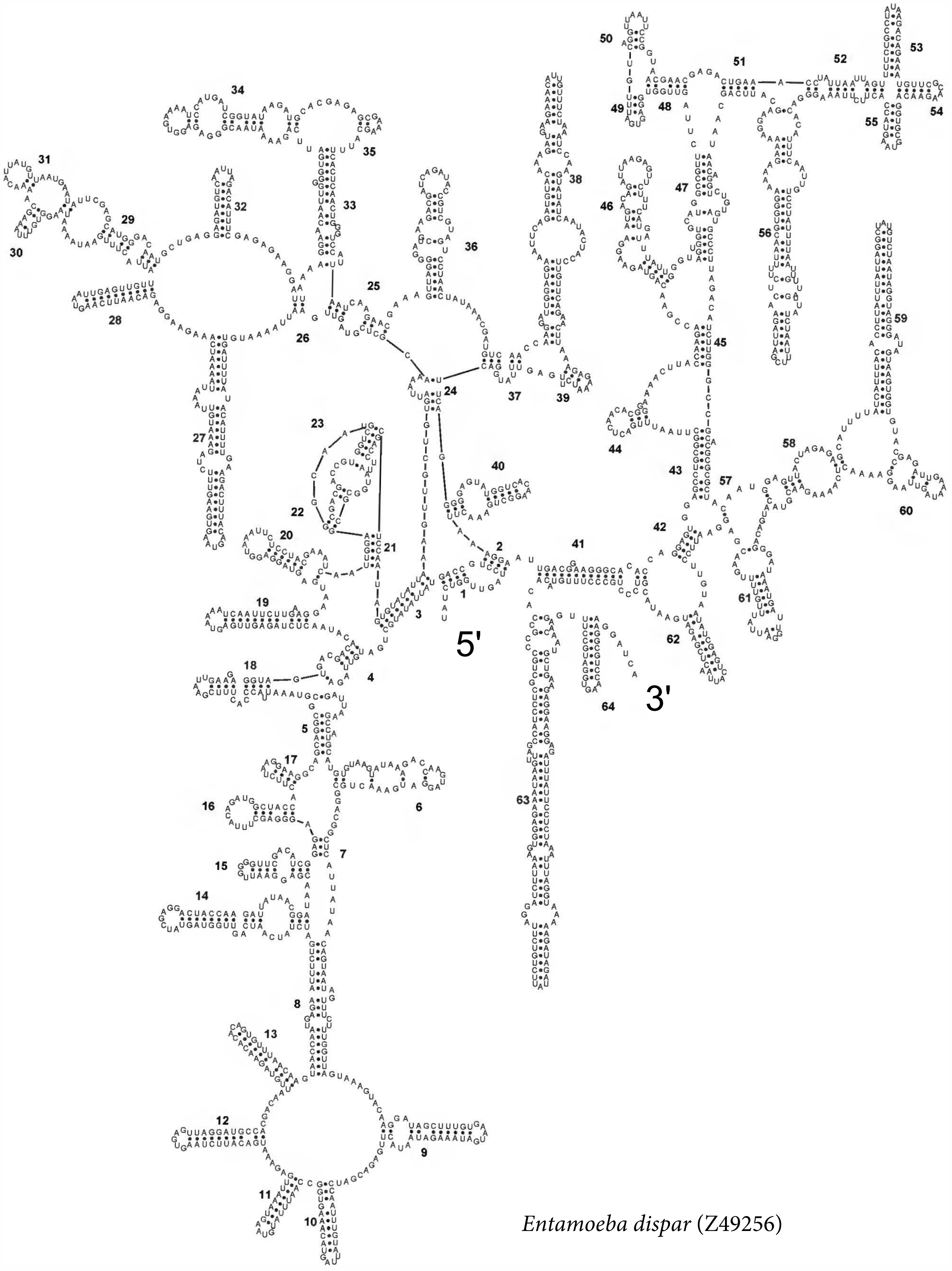

Entamoeba dispar (Z49256)

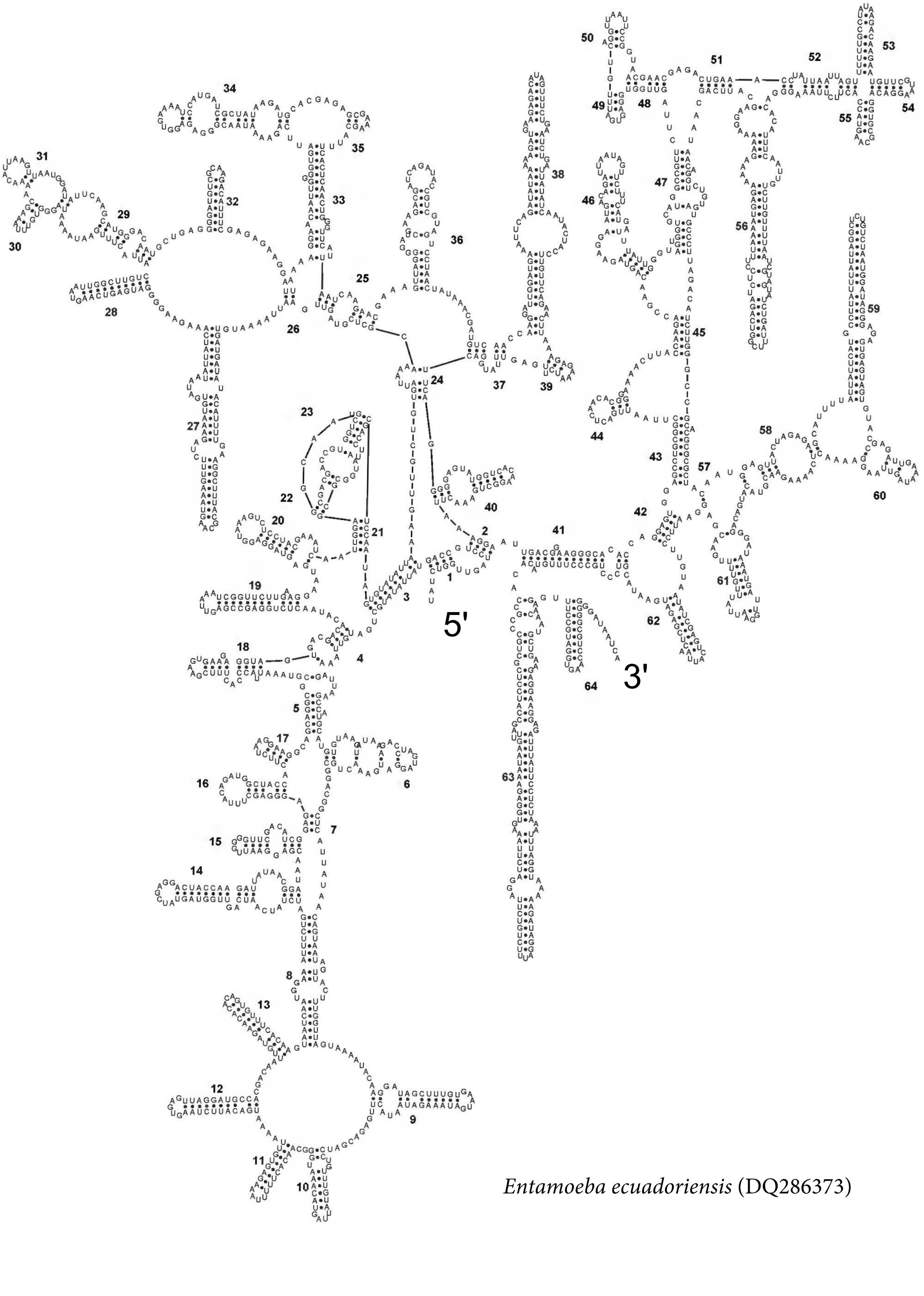

Entamoeba ecuadoriensis (DQ286373)

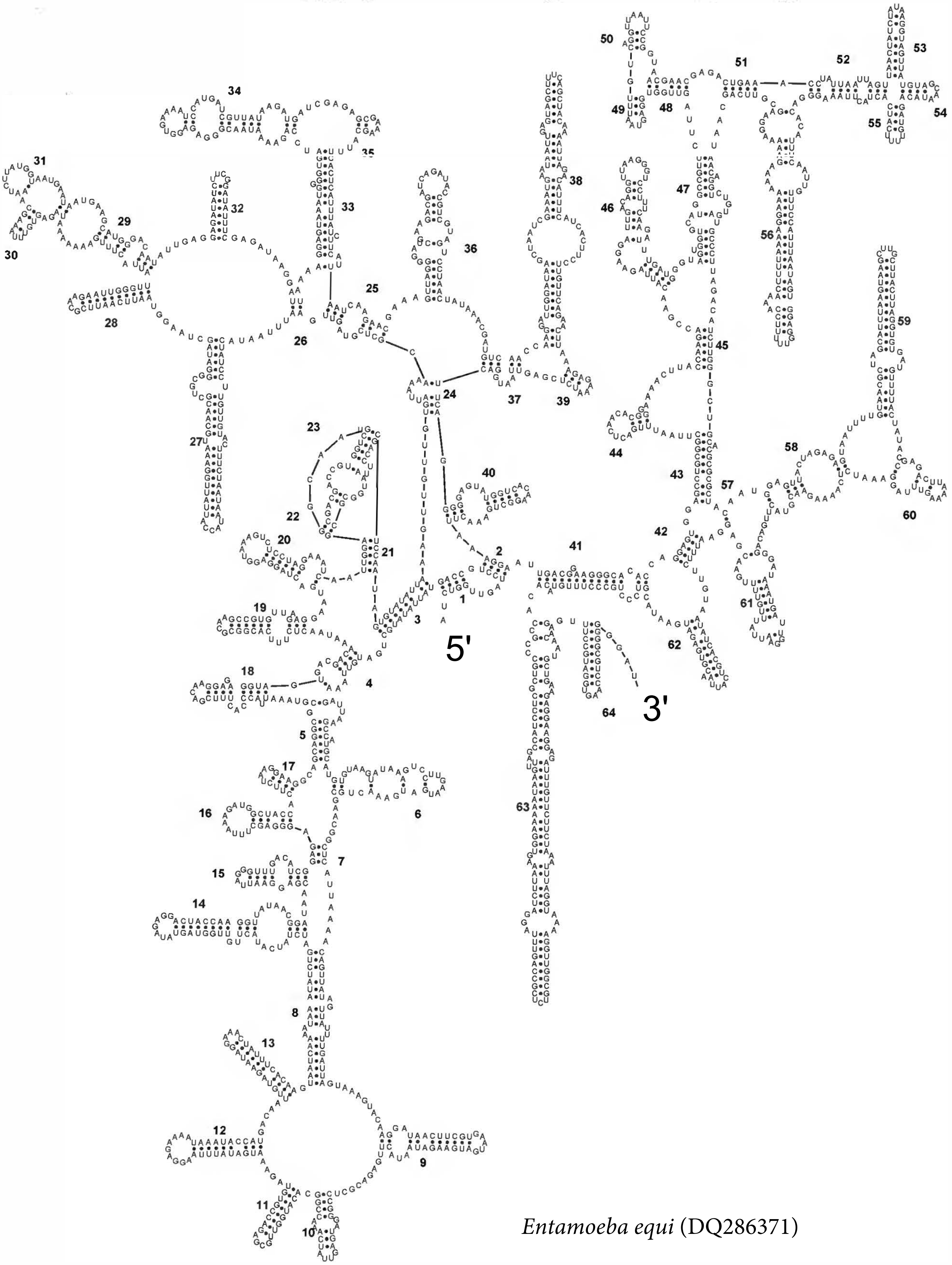

Entamoeba equi (DQ286371)

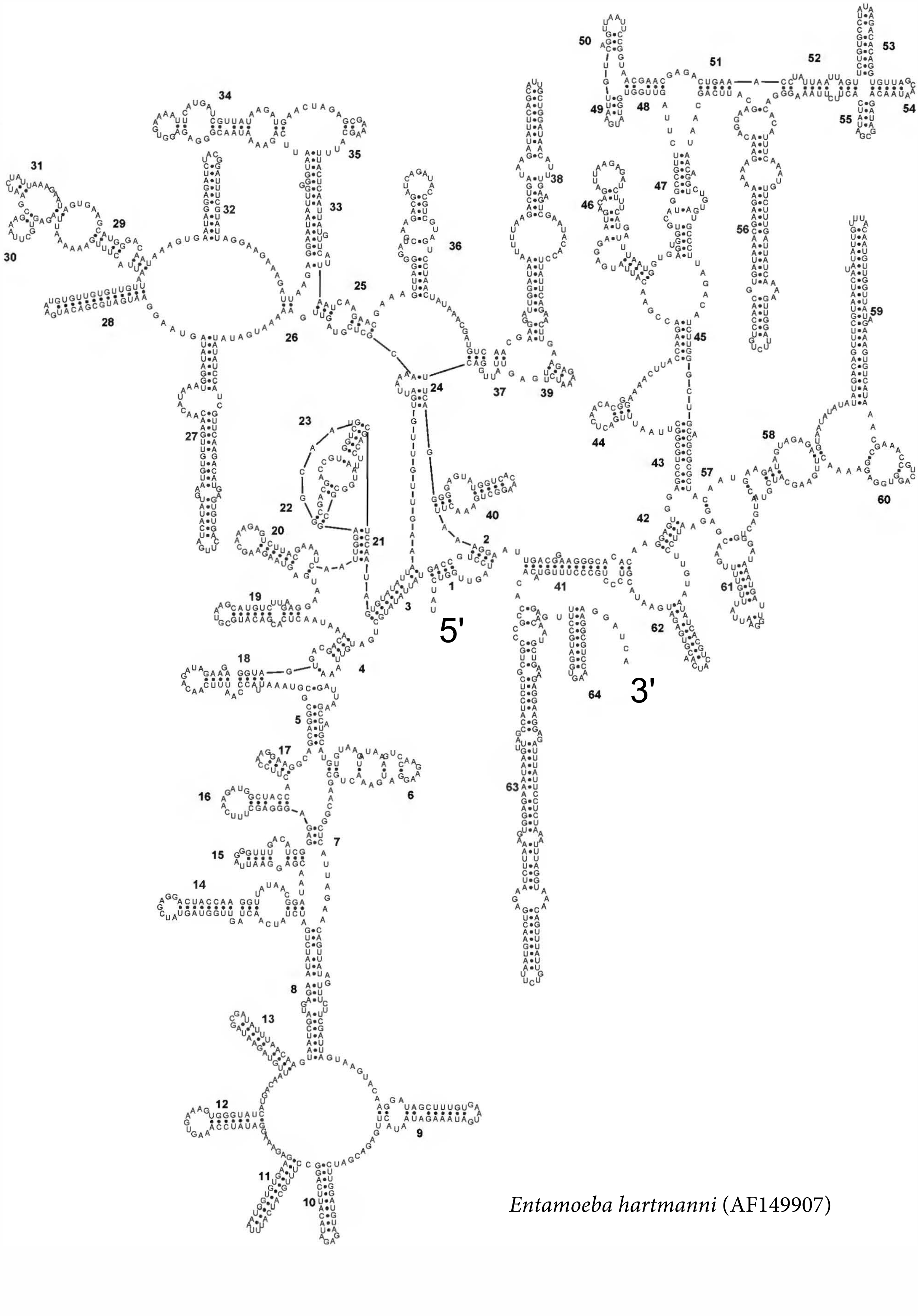

Entamoeba hartmanni (AF149907)

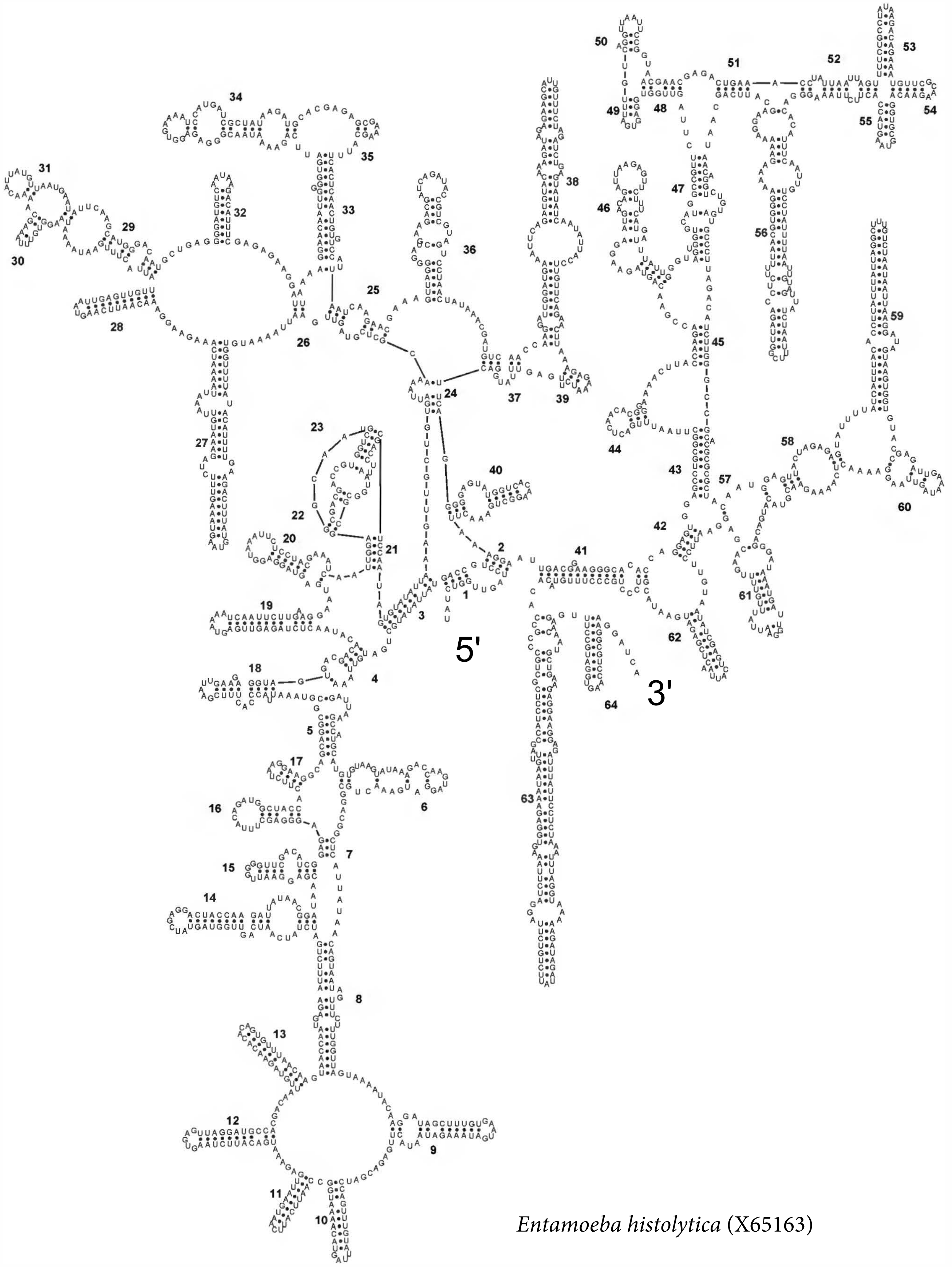

Entamoeba histolytica (X65163)

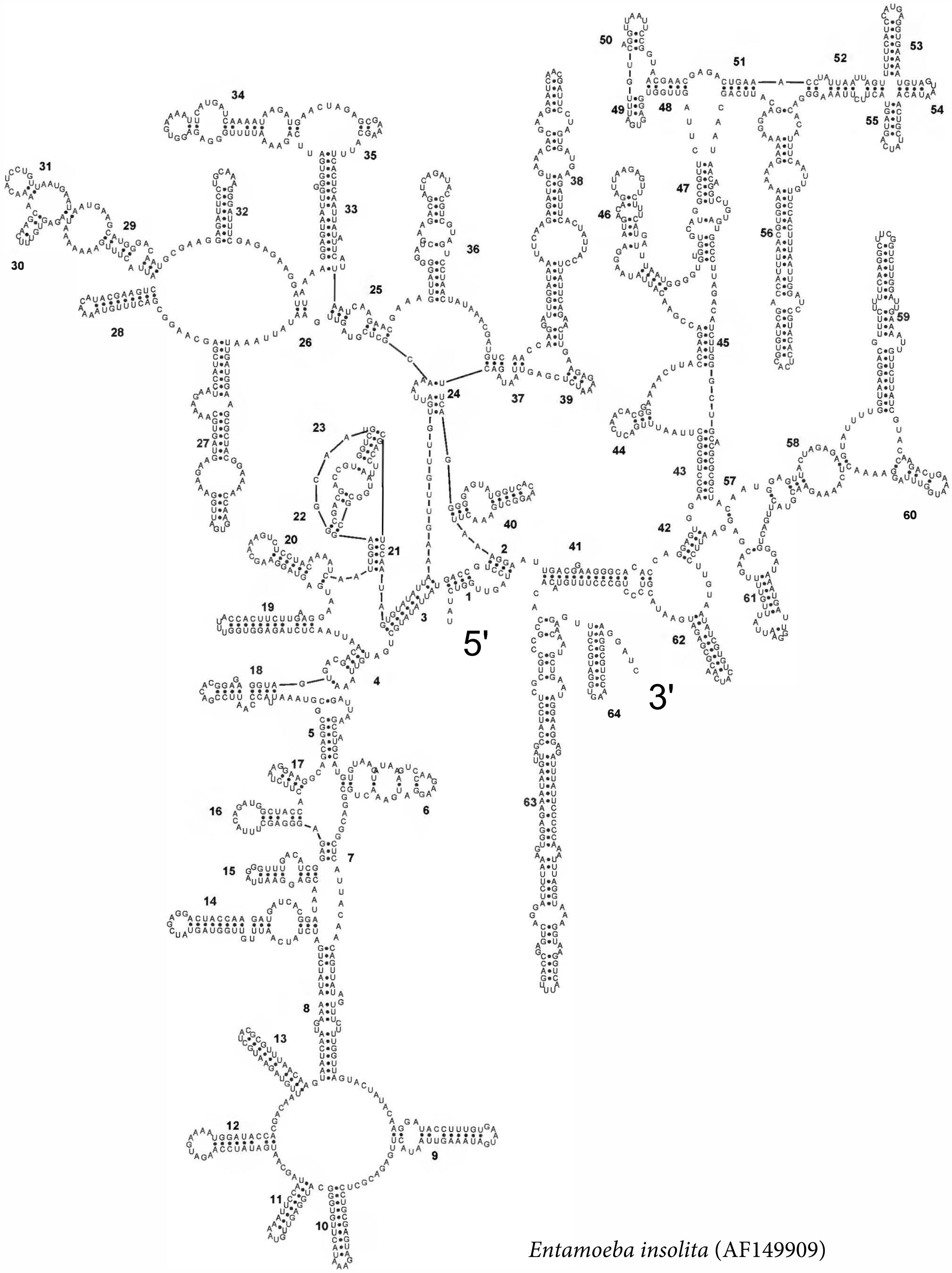

Entamoeba insolita (AF149909)

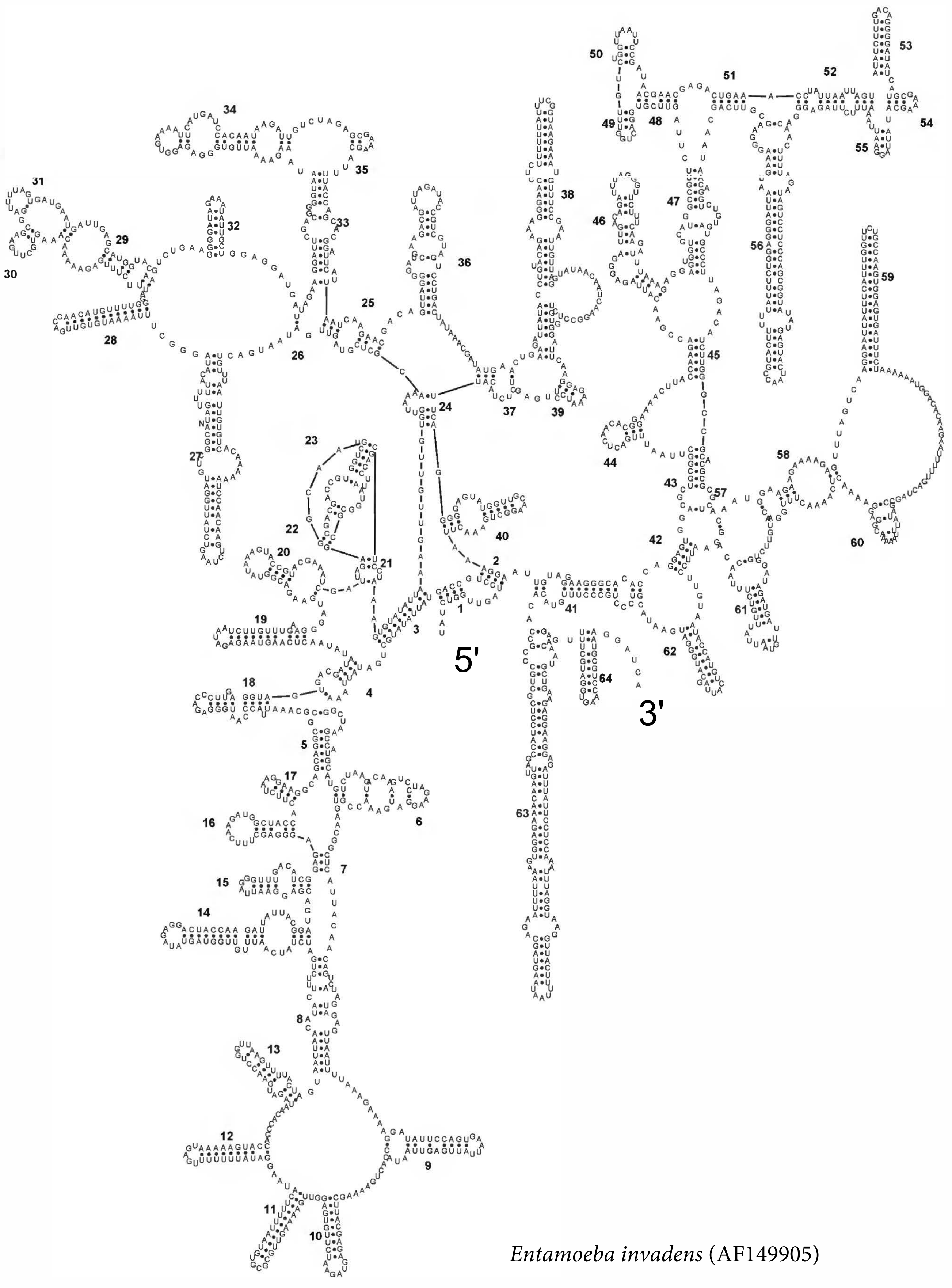

Entamoeba invadens (AF149905)

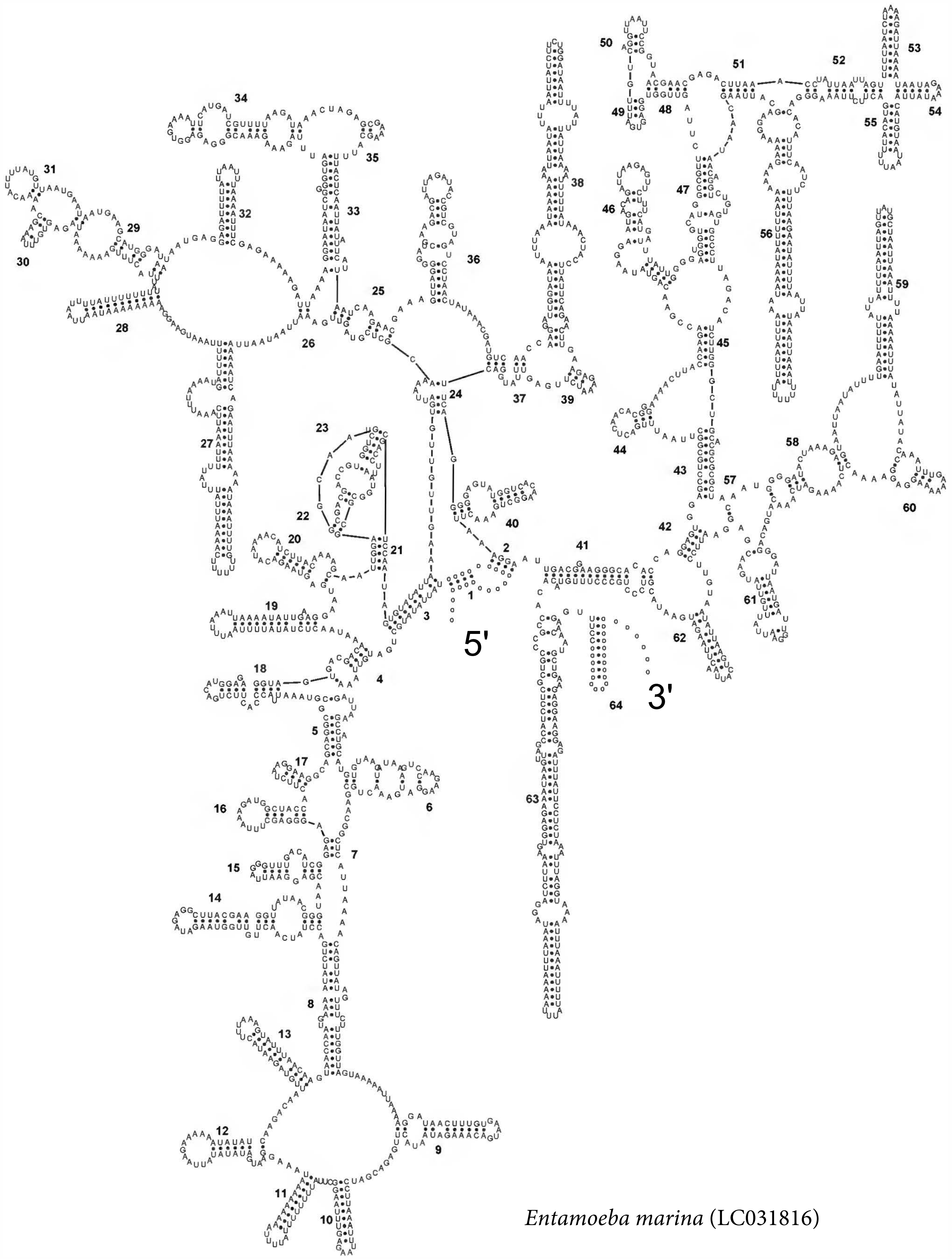

Entamoeba marina (LC031816)

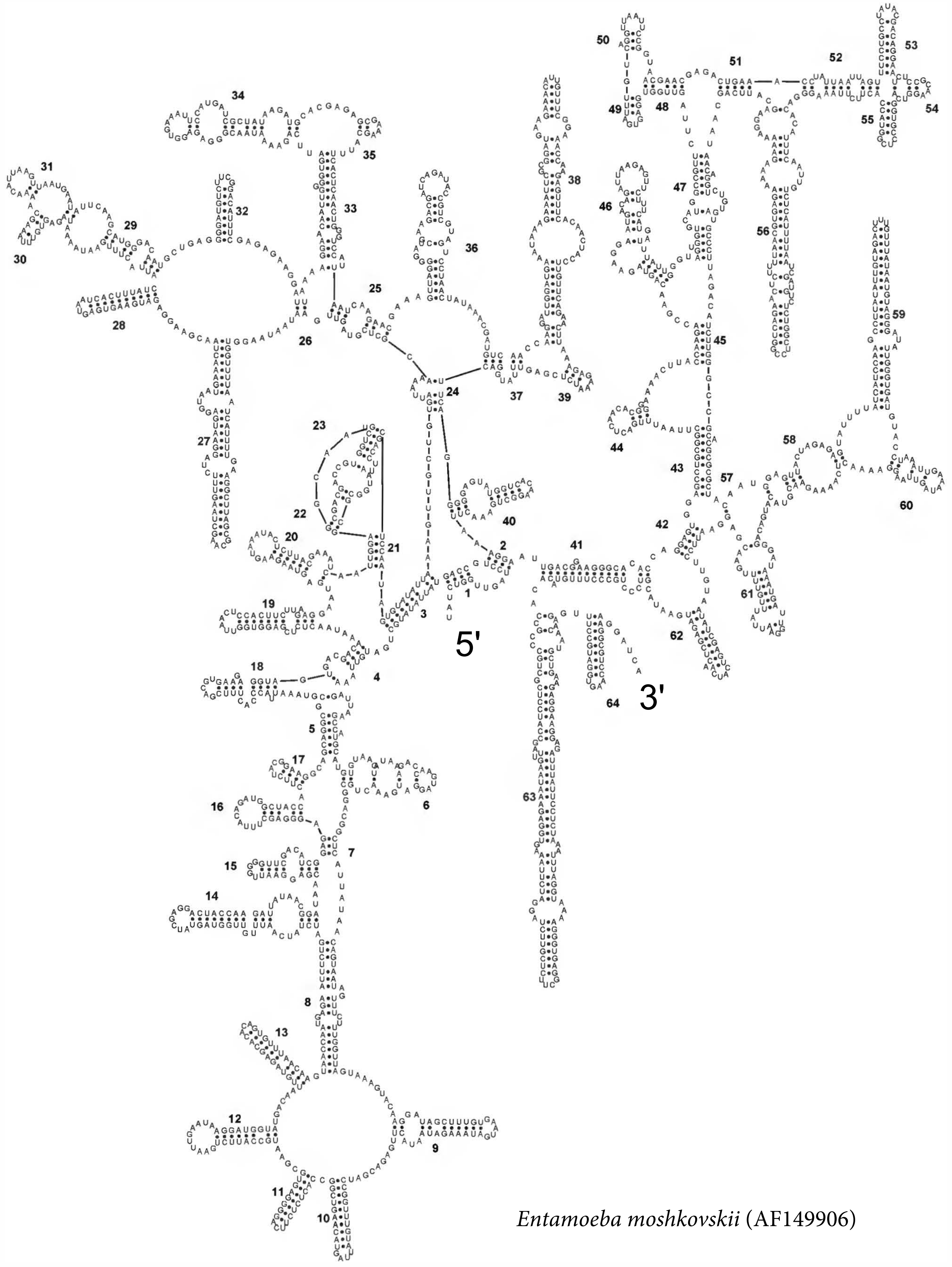

Entamoeba moshkovskii (AF149906)

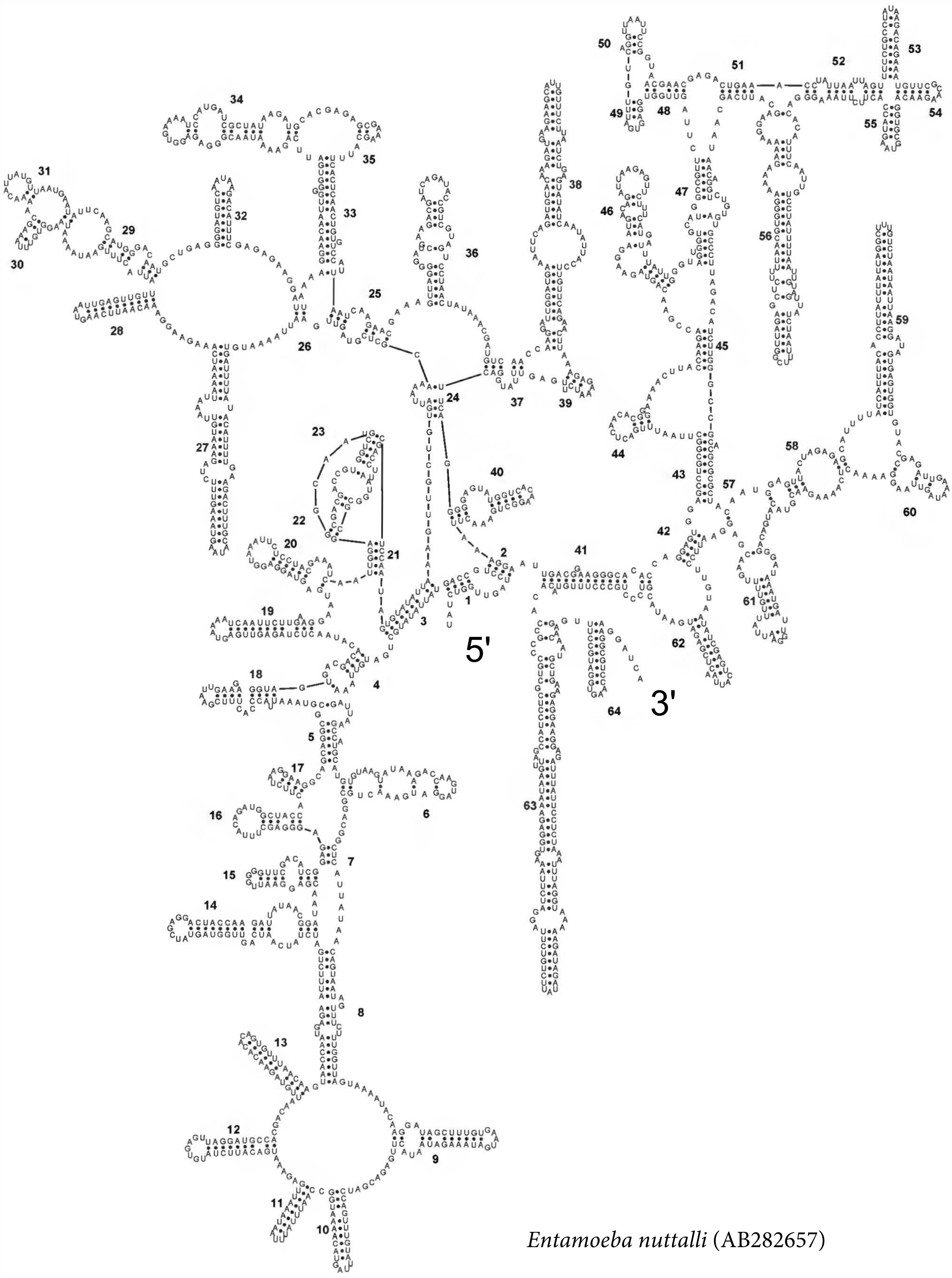

Entamoeba nuttalli (AB282657)

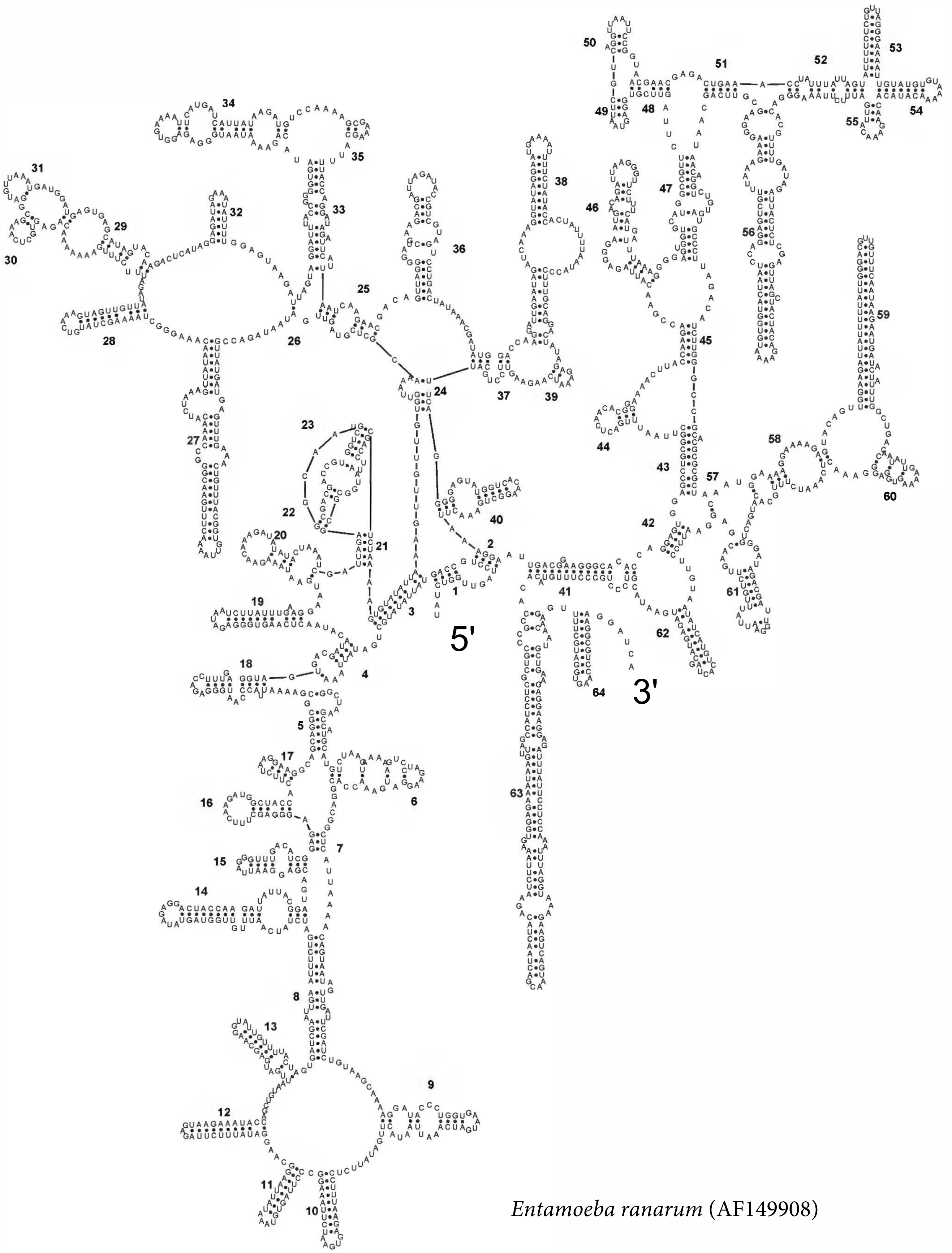

Entamoeba ranarum (AF149908)

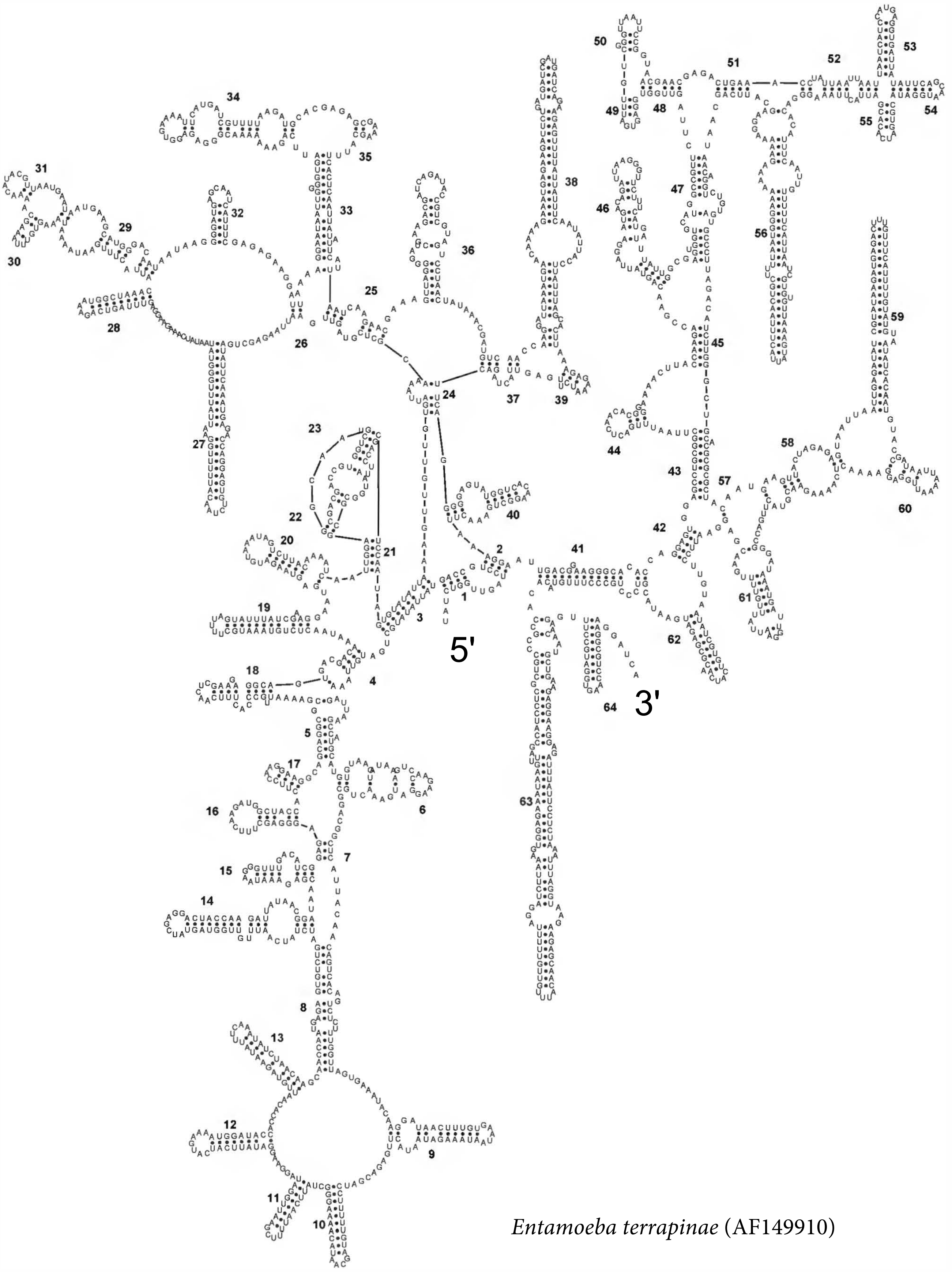

Entamoeba terrapinae (AF149910)

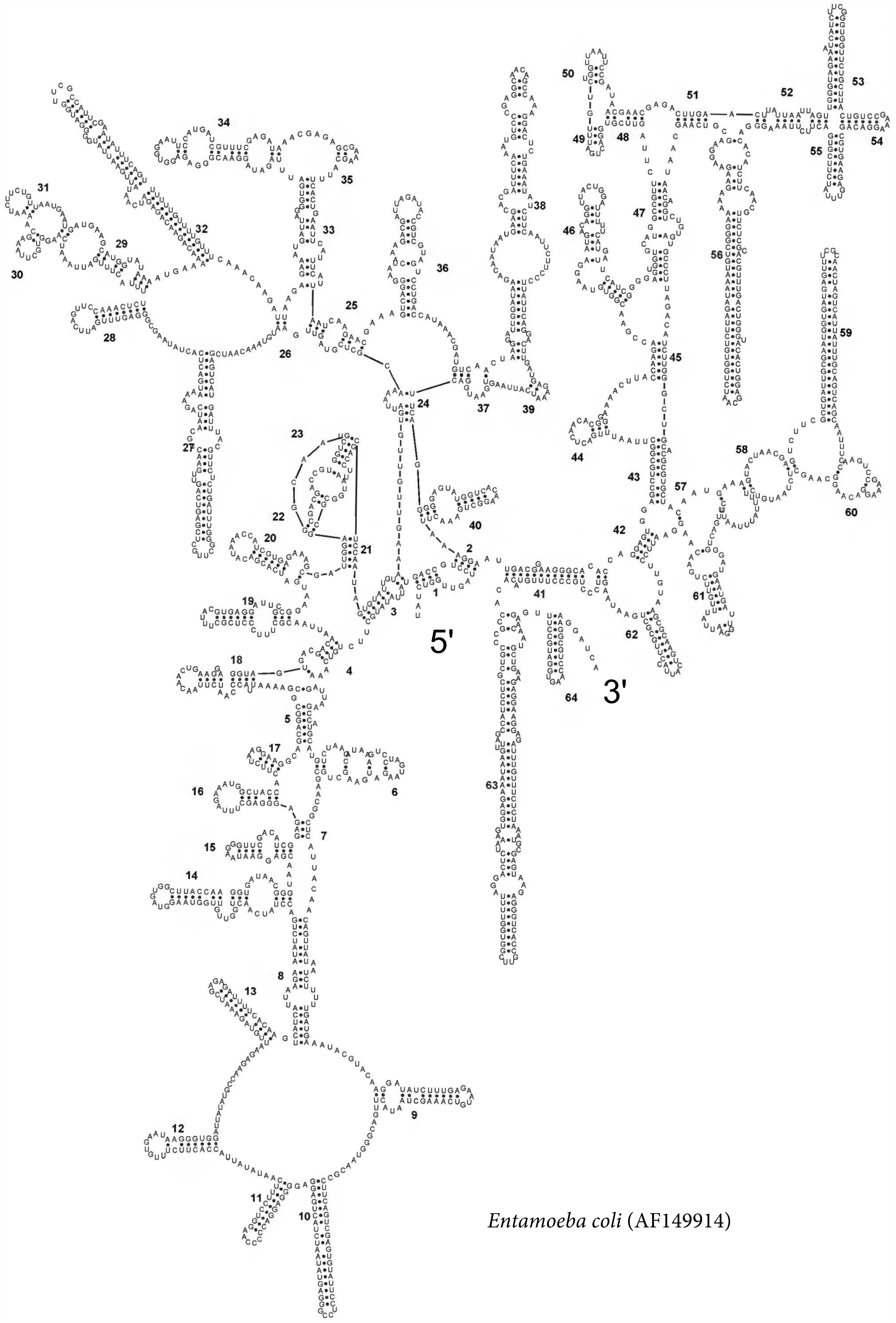

Entamoeba coli (AF149914)

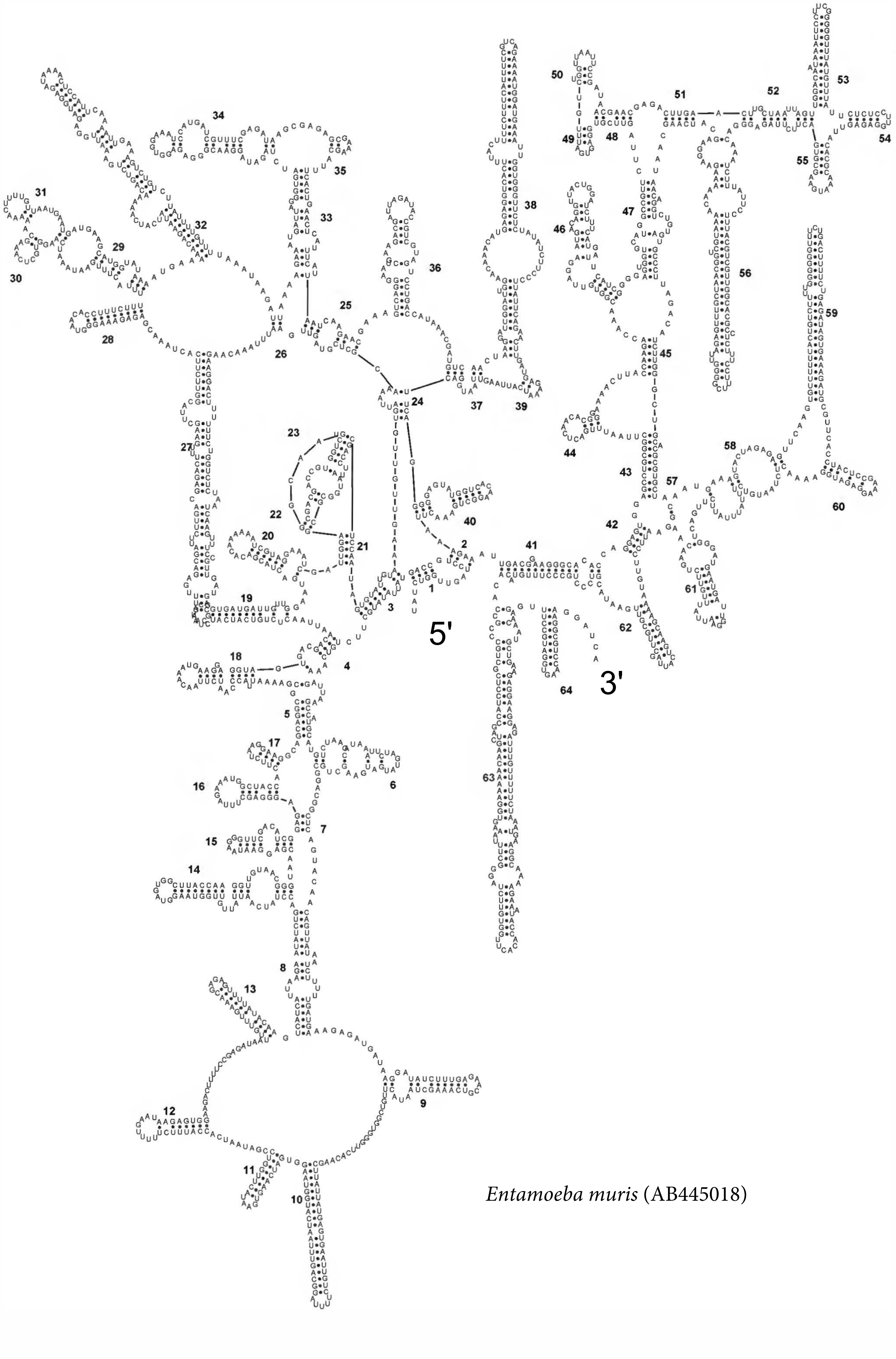

Entamoeba muris (AB445018)

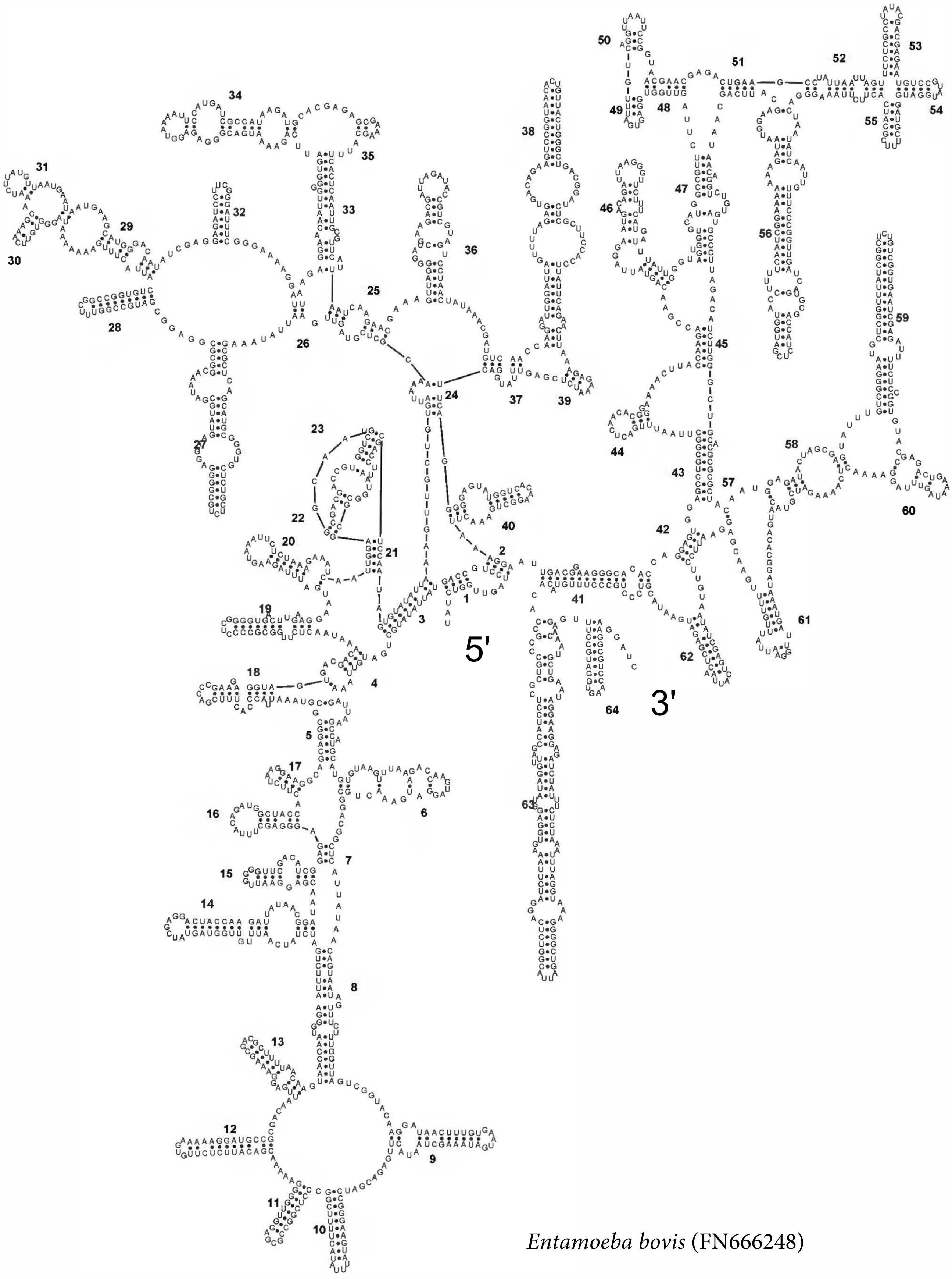

Entamoeba bovis (FN666248)

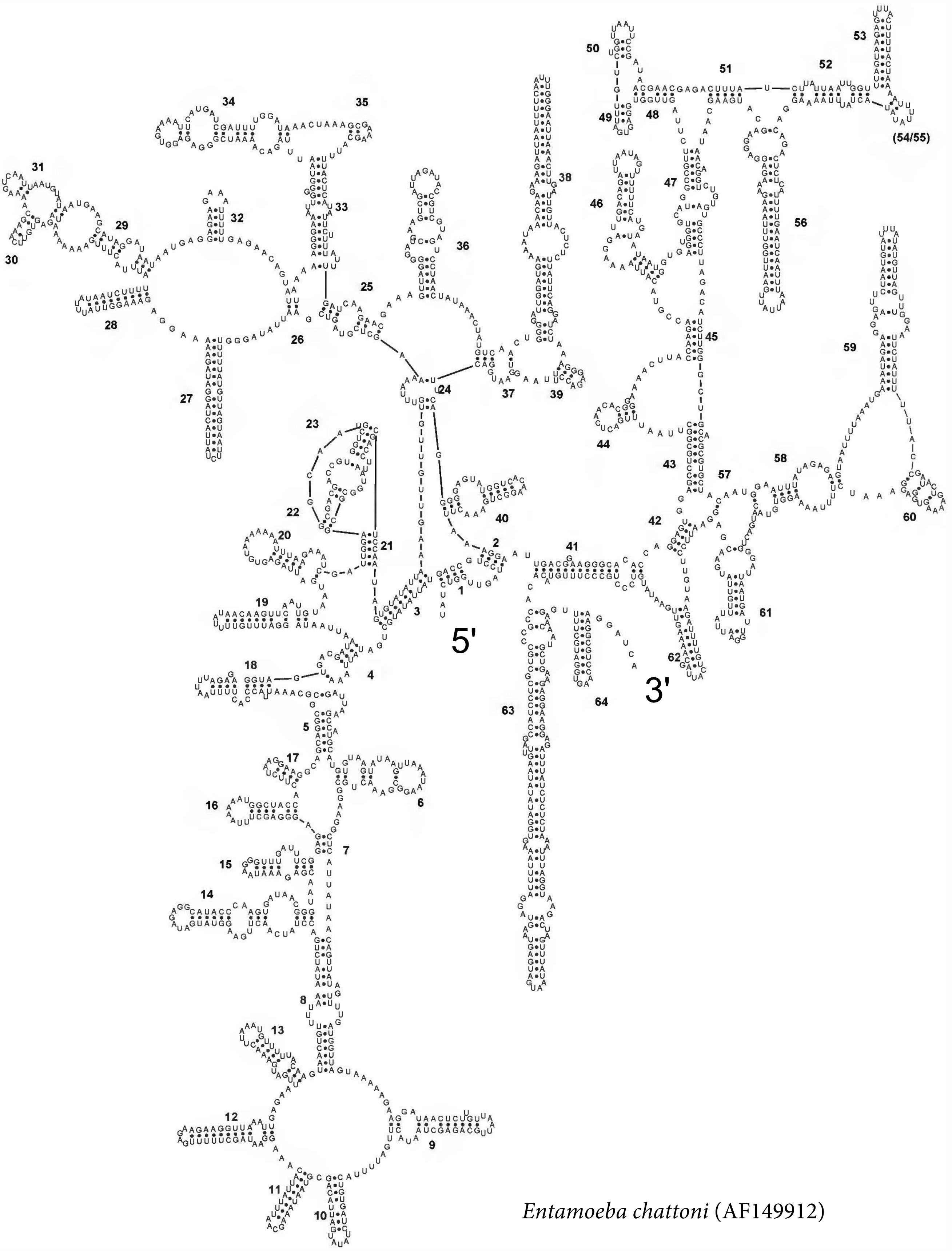

Entamoeba chattoni (AF149912)

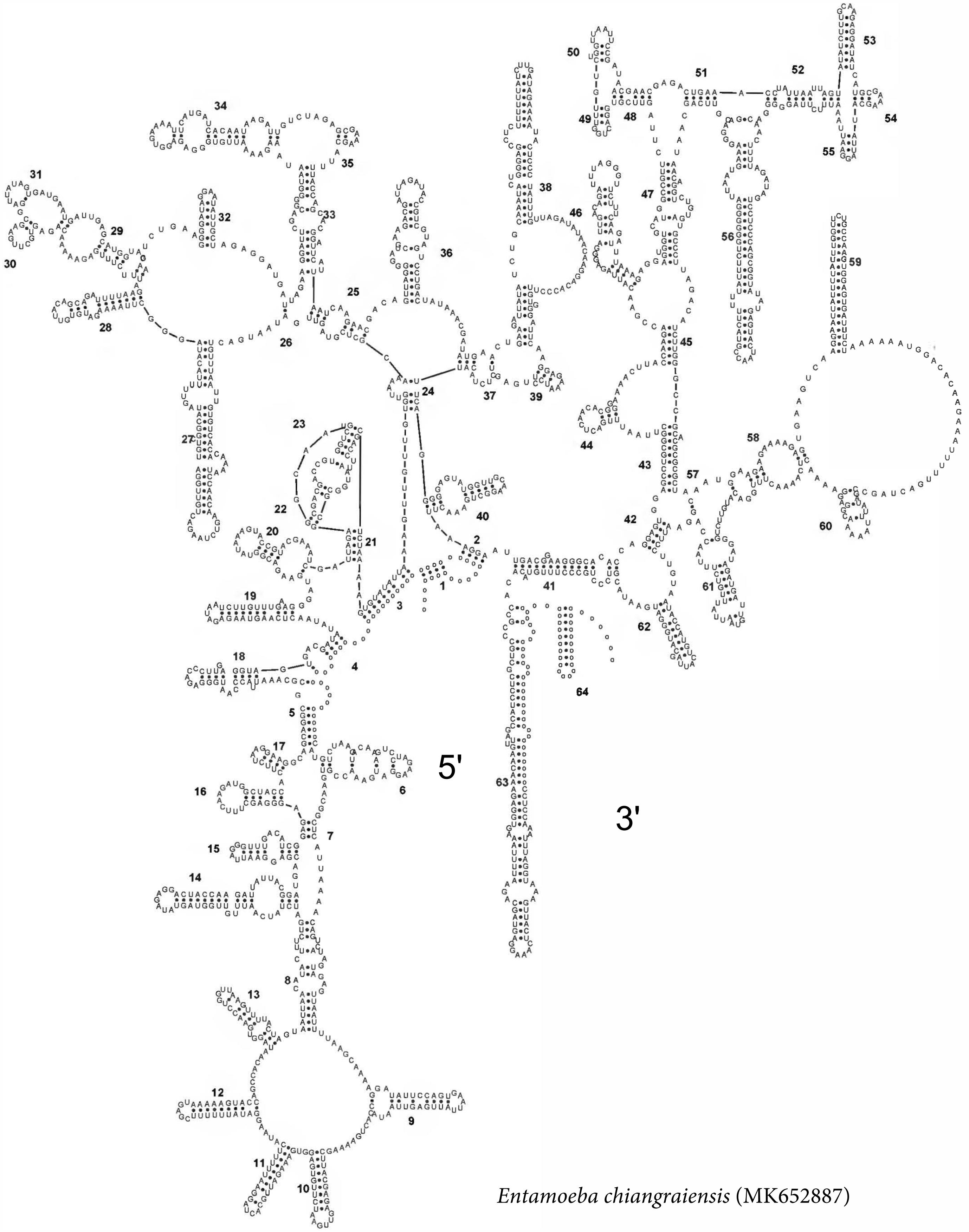

Entamoeba chiangraiensis (MK652887)

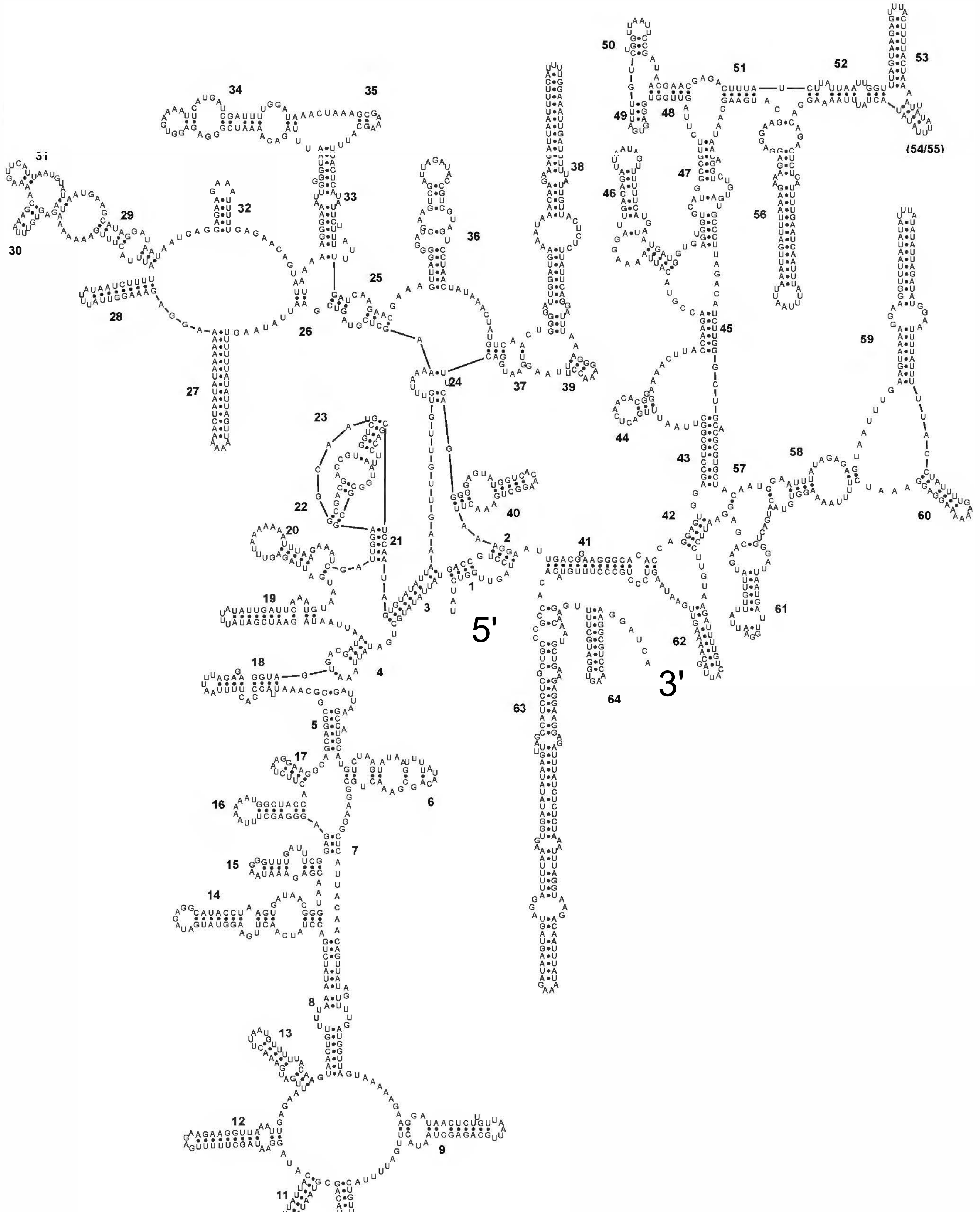

Entamoeba polecki (AF149913)

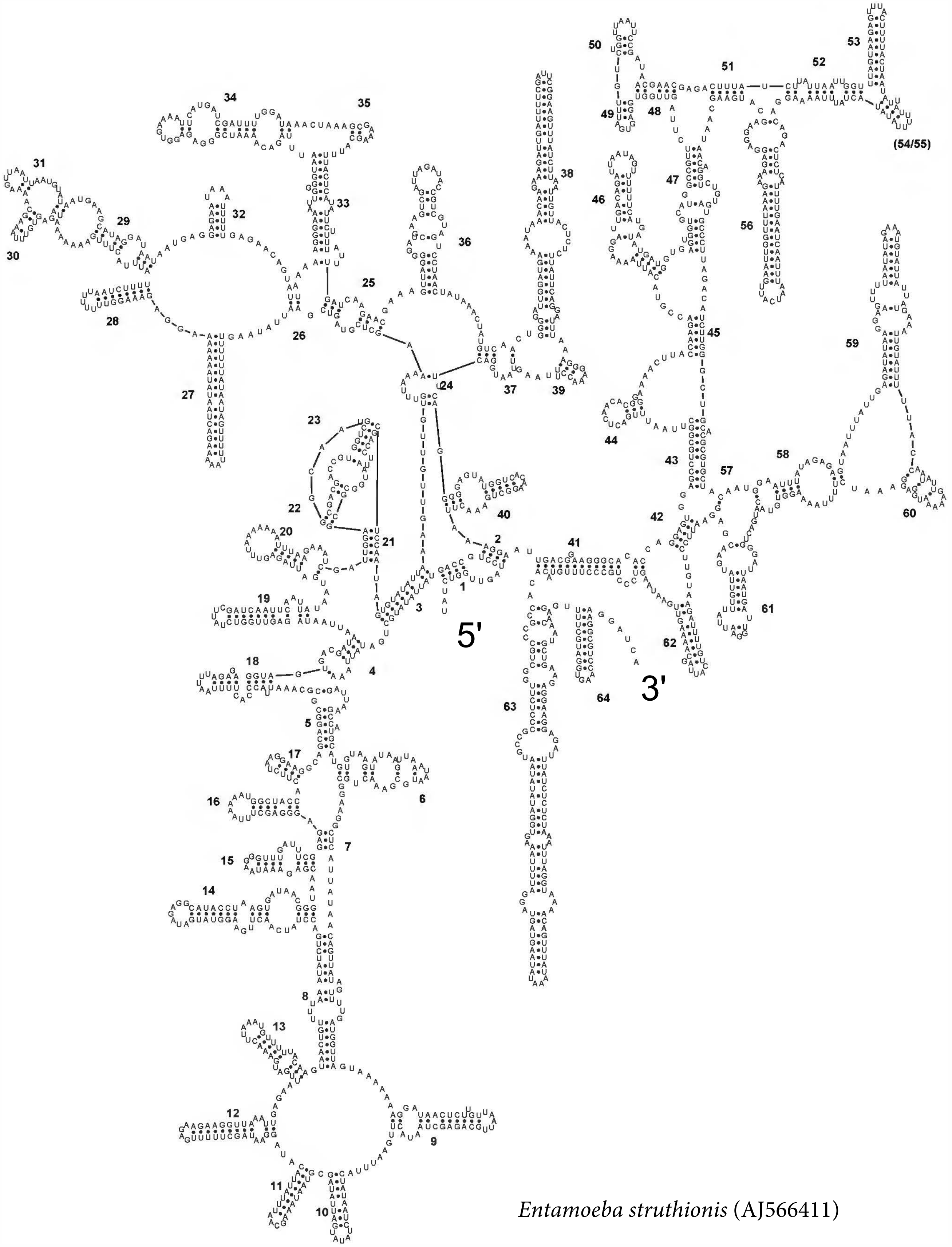

Entamoeba struthionis (AJ566411)

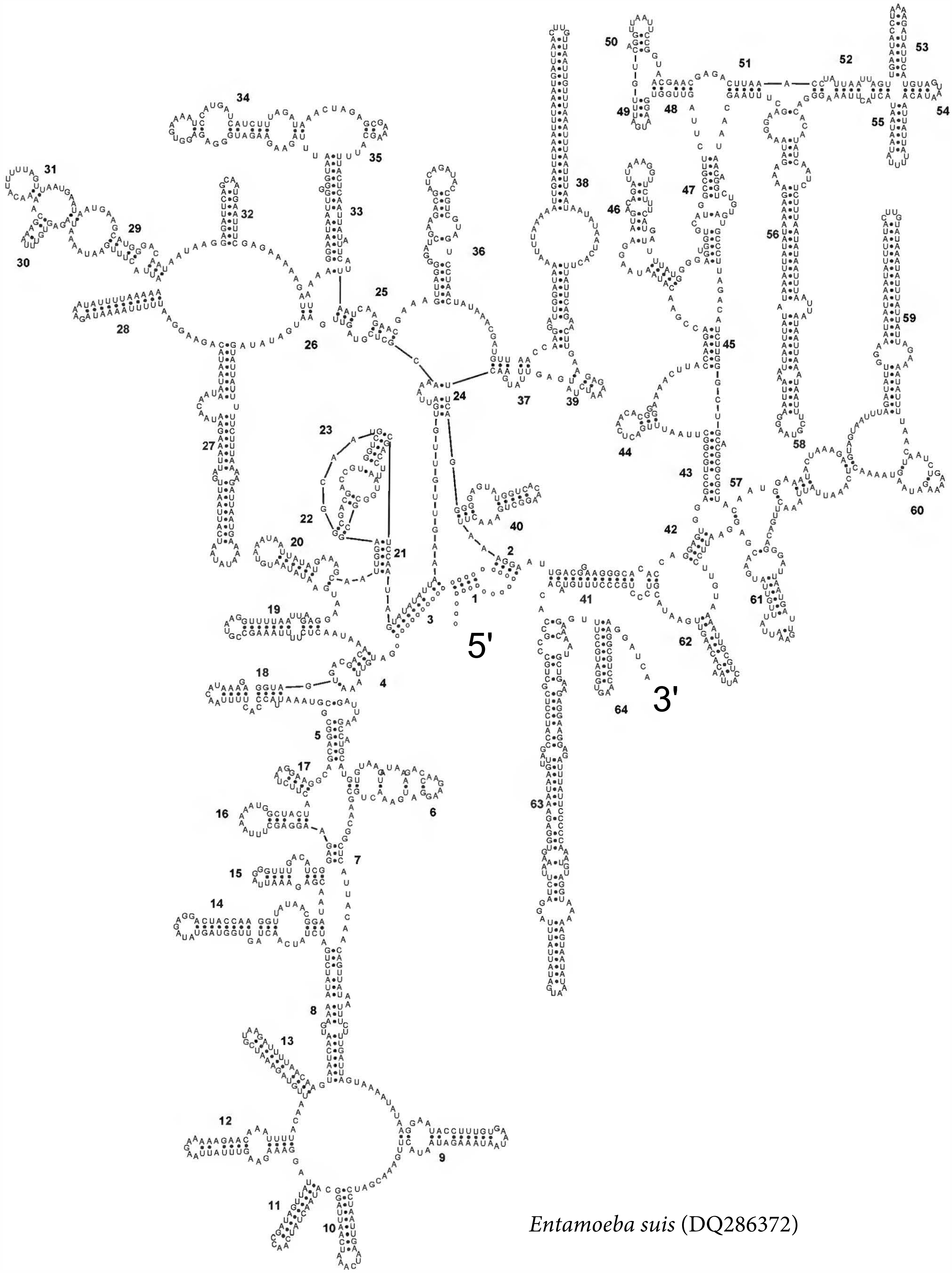

Entamoeba suis (DQ286372)

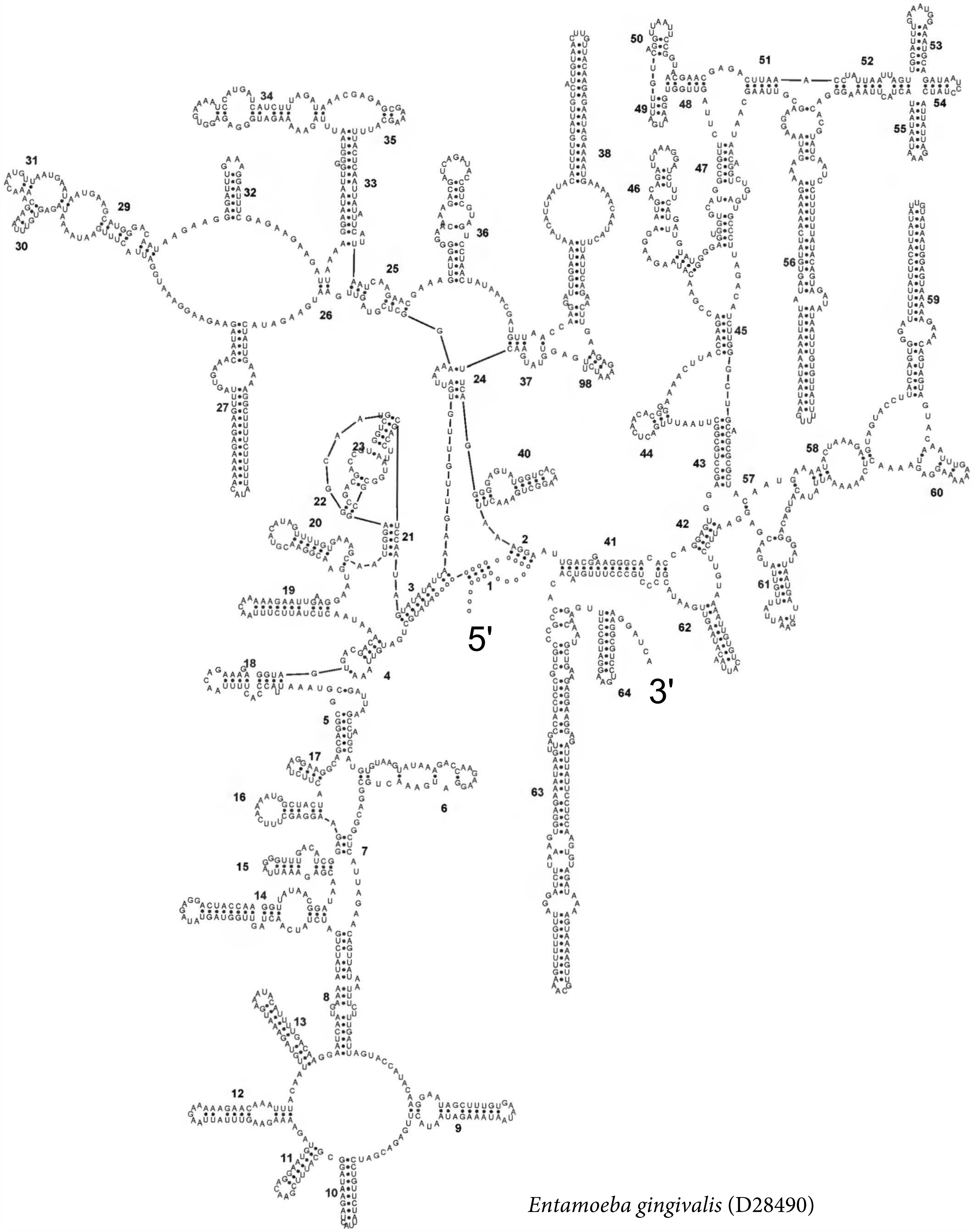

Entamoeba gingivalis (D28490)
