# Supplementary material for: Assessing Species Delimitation in Entamoeba (Amoebozoa: Endamoebidae) Using the Small Subunit rRNA Gene: Its Application to the Entamoeba polecki Complex
Source: Microorganisms. 2026 Feb 3;14(2):360. doi: 10.3390/microorganisms14020360 (PMC12942770; doi:10.3390/microorganisms14020360)

Supplementary File 3. Partition analysis of SSU rRNA gene sequences from *Entamoeba histolytica, Entamoeba nuttalli* and *Entamoeba dispar* performed using ASAP.

Analyses were conducted separately for each of the four structural domains of the SSU rRNA molecule. For each domain, sequences were trimmed according to domain boundaries, and only sequences (or sequence fragments) covering at least 75% of the length of the corresponding domain were included in the analysis. Sequences marked with asterisk (*) were identified in Genbank as belonging to another species but were reannotated in this study. Color dots at the nodes indicate group probability: red, <0.05; yellow: >0.1; grey, not applicable.

DOMAIN: 5′ MAJOR

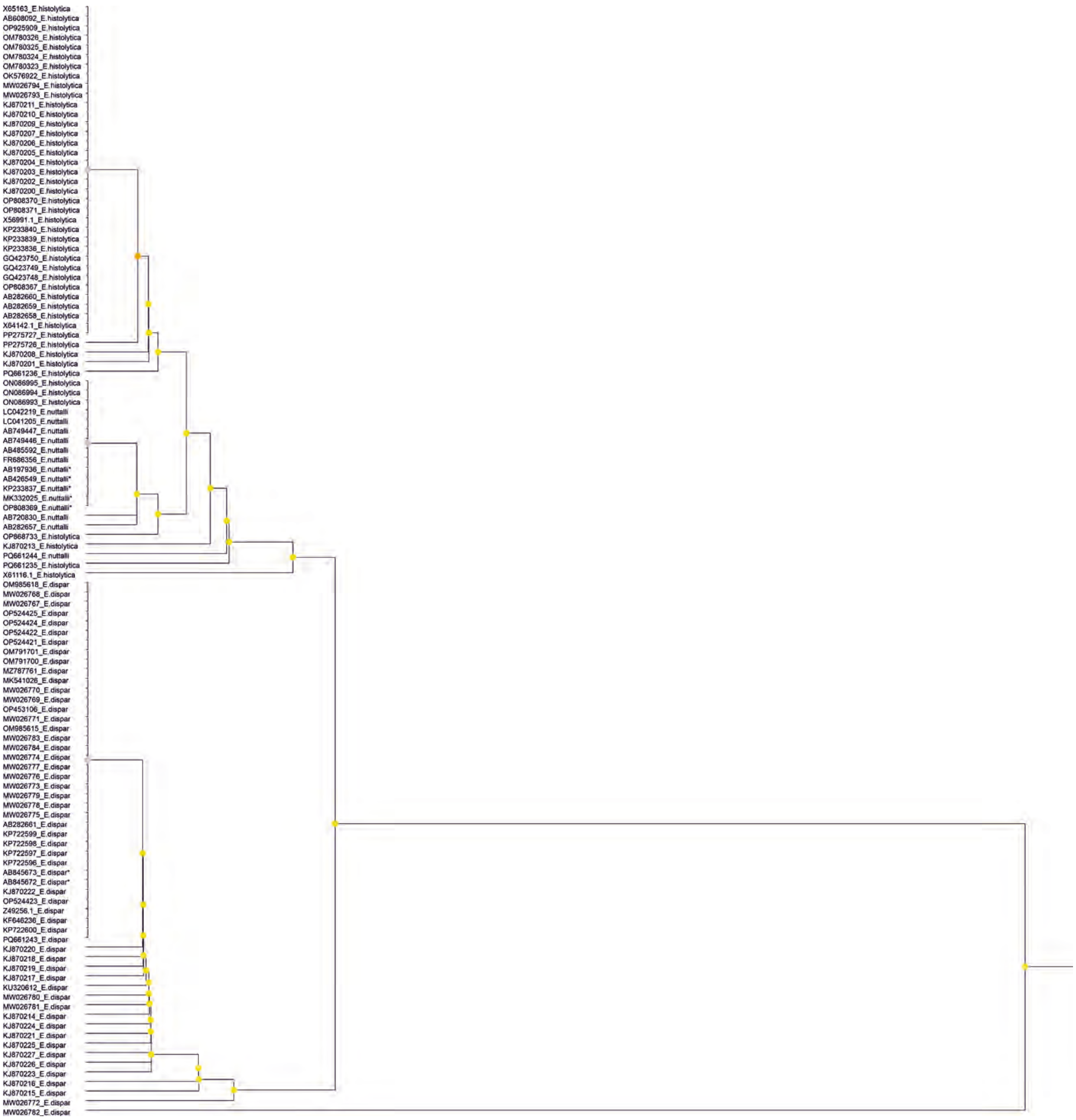

DOMAIN: CENTRAL

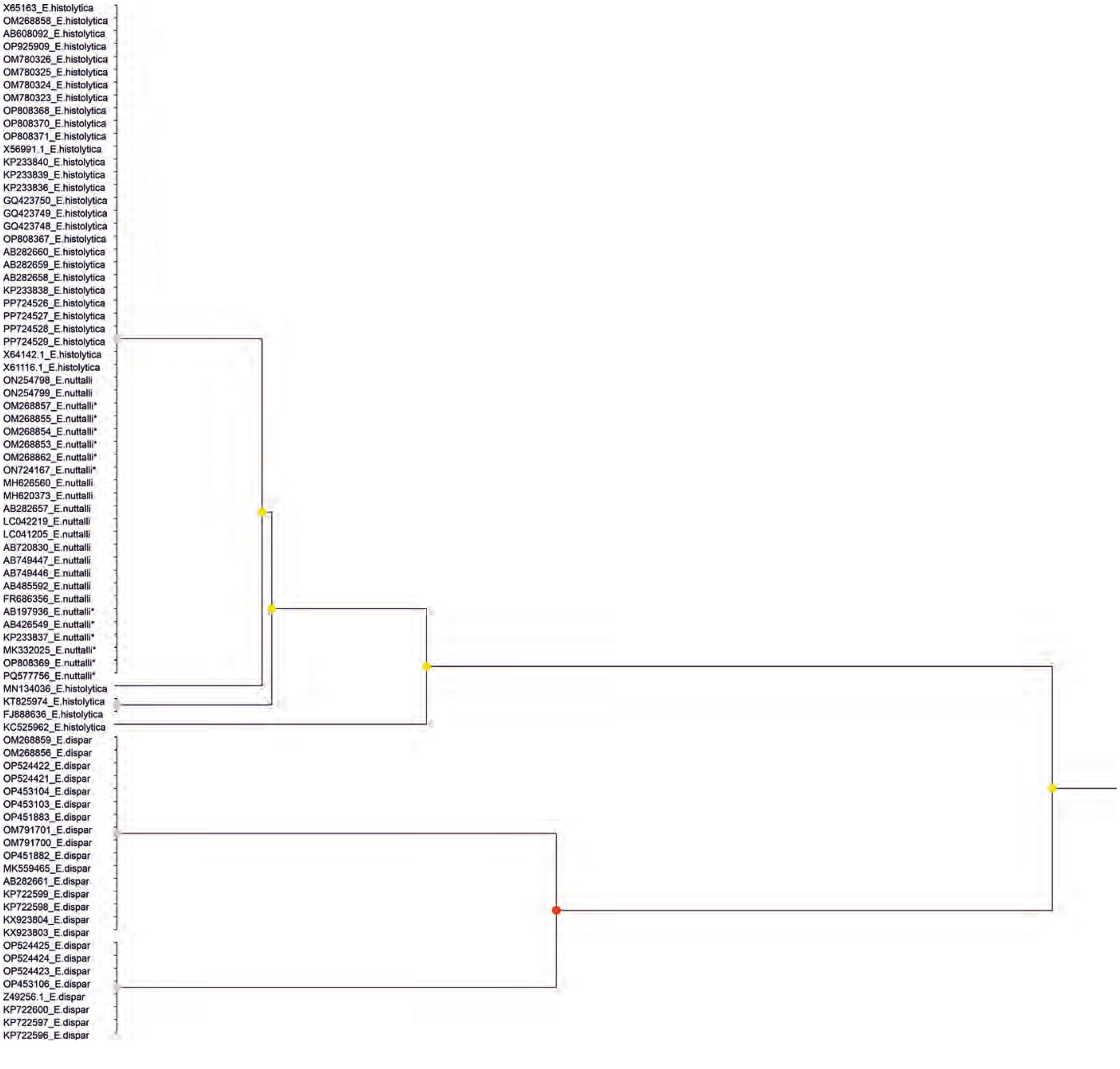

DOMAIN: 3′ MAJOR

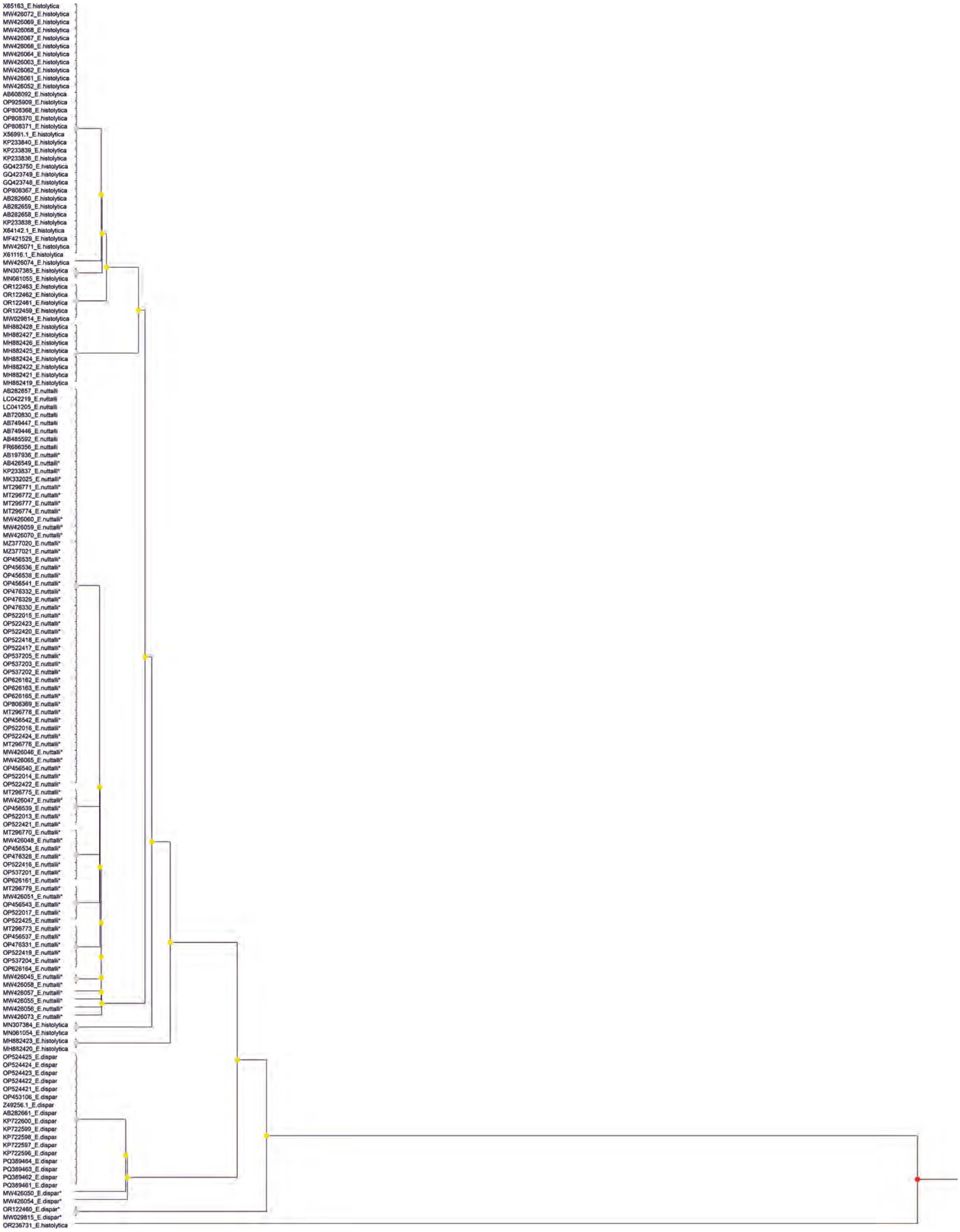

DOMAIN: 5′ MINOR

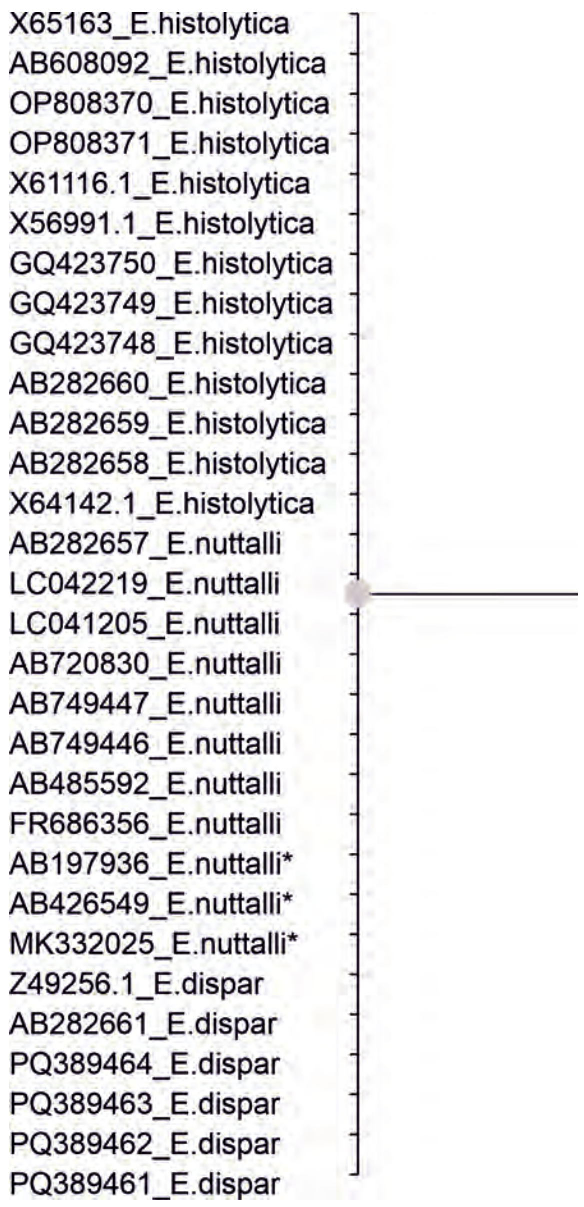

Supplement: Supplementary file 1 [file microorganisms-14-00360-s001.zip › Supplementary File 3.pdf]
