# Supplementary material for: Assessing Species Delimitation in Entamoeba (Amoebozoa: Endamoebidae) Using the Small Subunit rRNA Gene: Its Application to the Entamoeba polecki Complex
Source: Microorganisms. 2026 Feb 3;14(2):360. doi: 10.3390/microorganisms14020360 (PMC12942770; doi:10.3390/microorganisms14020360)
Supplement: Supplementary file 1 [file microorganisms-14-00360-s001.zip › Supplementary File 5.pdf]

Supplementary File 5. Secondary structure diagrams of the SSU rRNA molecule of *Entamoeba histolytica*, *Entamoeba nuttalli*, *Entamoeba dispar*, *Entamoeba polecki* sensu stricto, *Entamoeba struthionis* and *Entamoeba chattoni* highlighting variable positions.

For each species, a consensus sequence was generated using a majority-rule approach at each alignment position. To ensure accurate positional mapping of sequence variation, the consensus sequence retained the full alignment length, with insertion-deletion (indel) positions preserved even when a nucleotide was present in only a single sequence. For each species, two diagrams are shown: the one on the left indicates positions at which nucleotide or indel differences relative to the majority base occur in at least one of the analysed sequences, whereas the one on the right indicates positions at which differences relative to the majority base are present in at least two sequences. Colour codes indicate the frequency of sequence variation relative to the majority base at each position: blue, variation present in <10% of the sequences covering the position; green, 10-20%; orange, 20-30%; purple, 30-40%; red, 40-50%.

In the structures corresponding to *E. polecki*, *E. struthionis* and *E. chattoni*, one helix is absent in the region corresponding to expansion segment 12 / variable region 9. As the aim of this study was not to establish helix-to-helix correspondence, the missing helix was not unambiguously identified as helix 54 or 55 as is therefore provisionally labelled as helix (54/55).

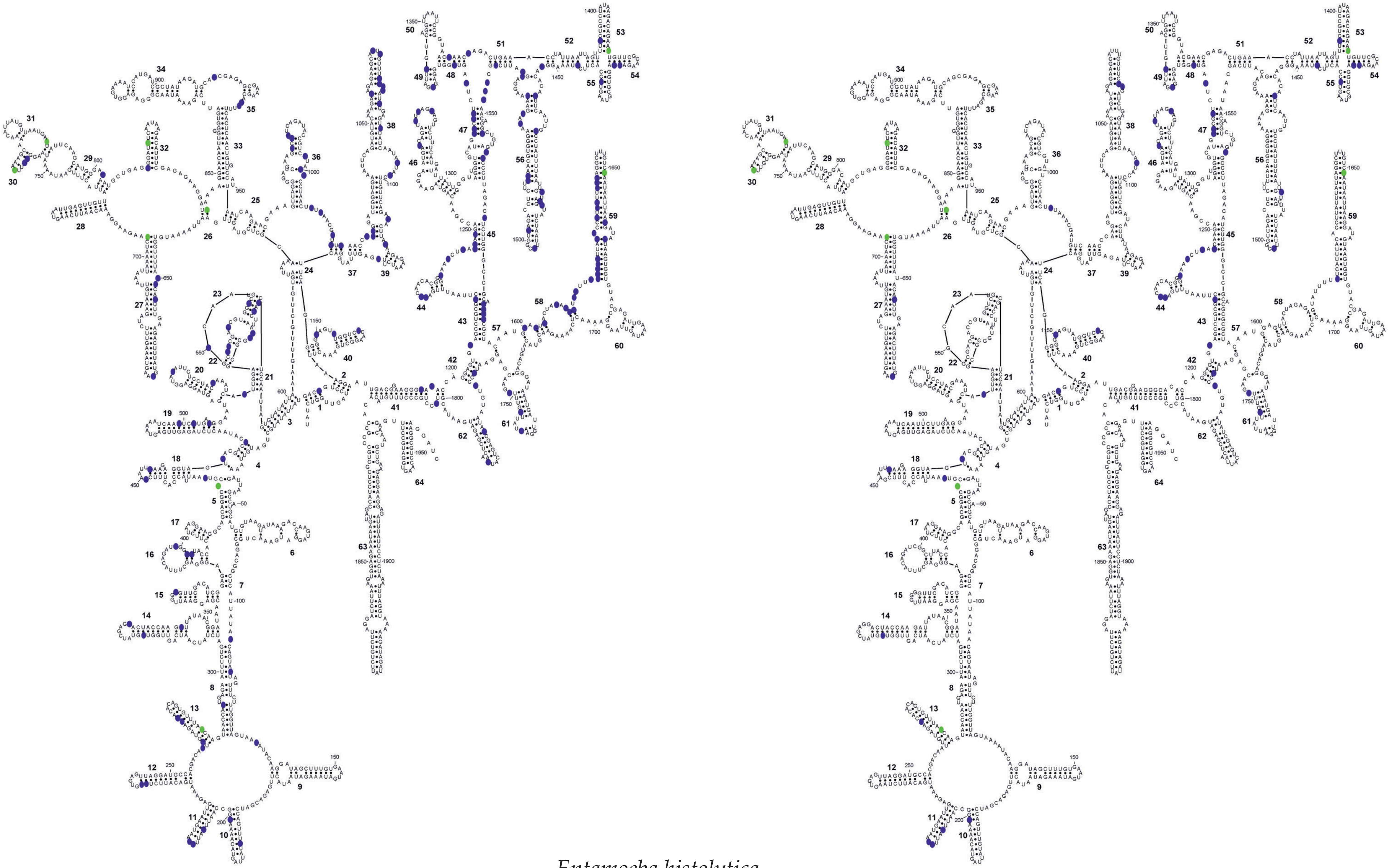

Entamoeba histolytica

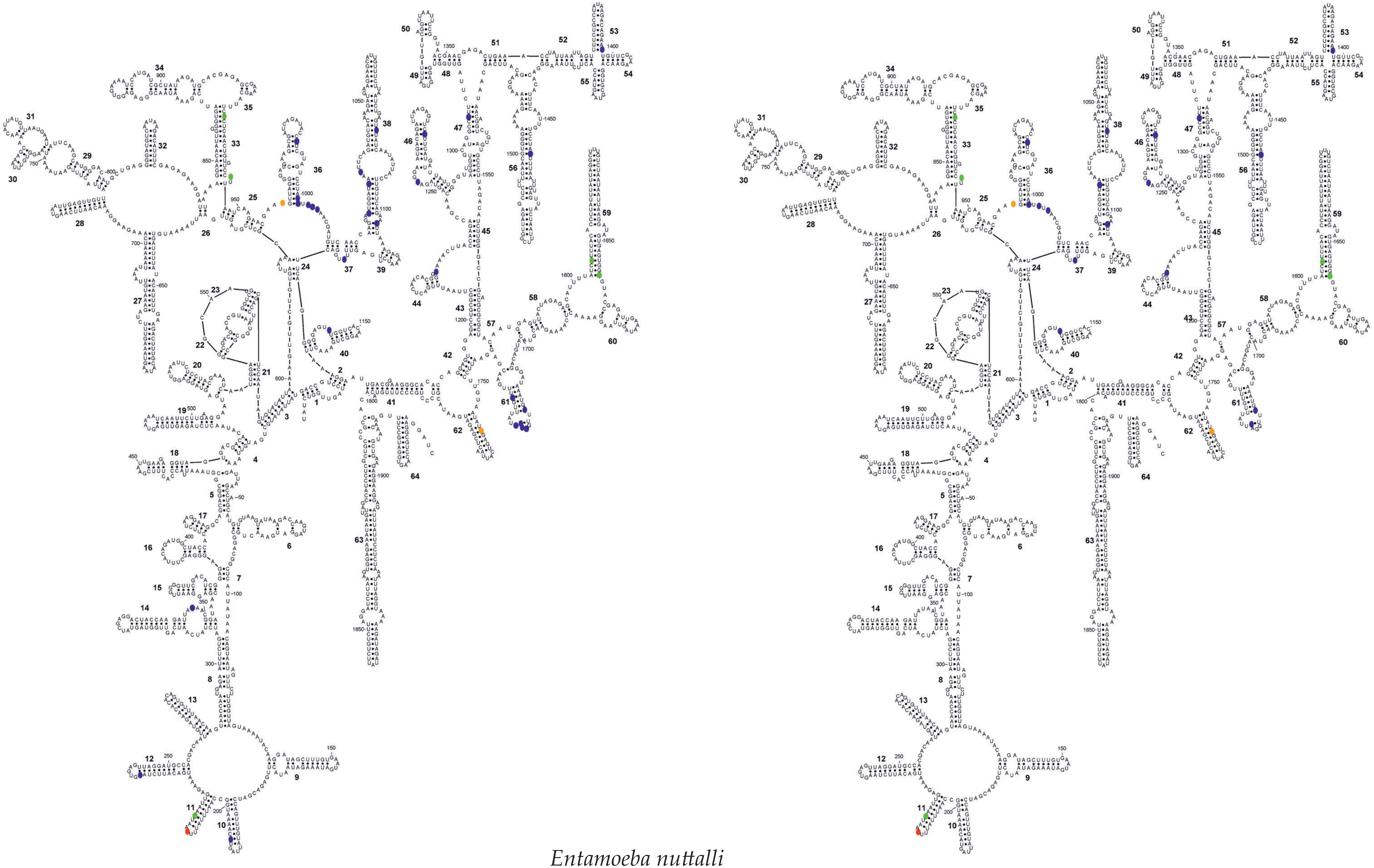

Entamoeba nuttalli

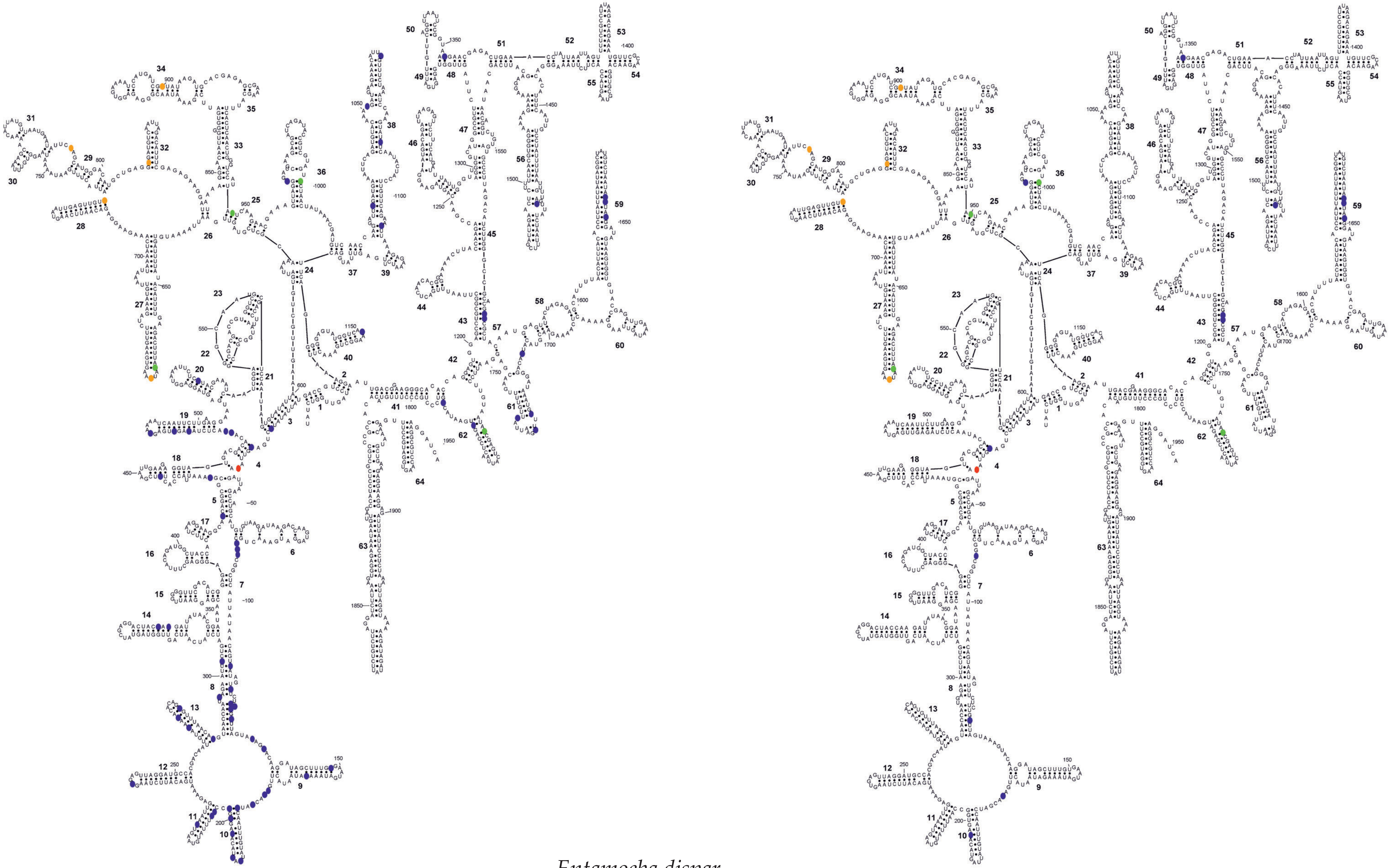

Entamoeba dispar

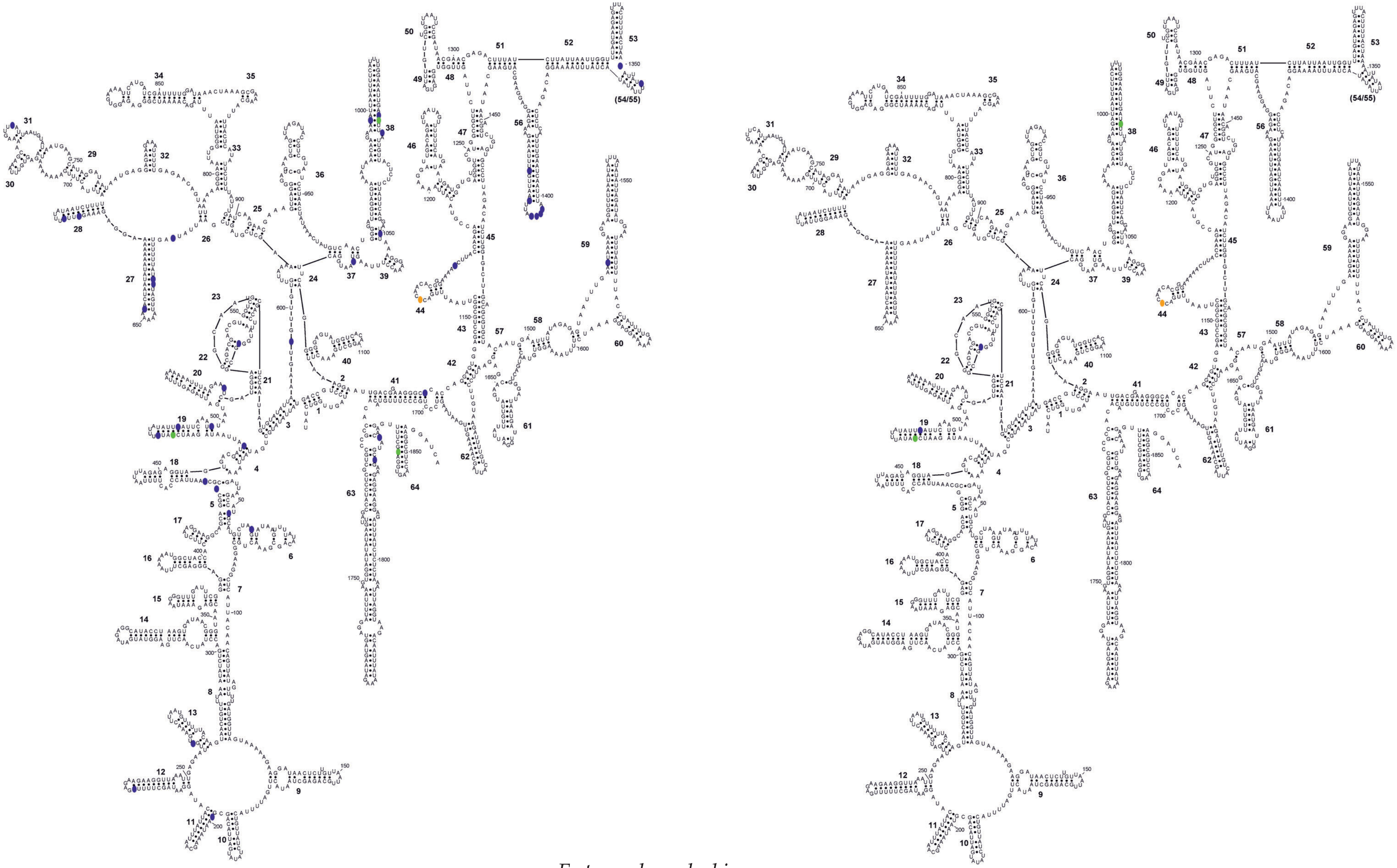

Entamoeba polecki

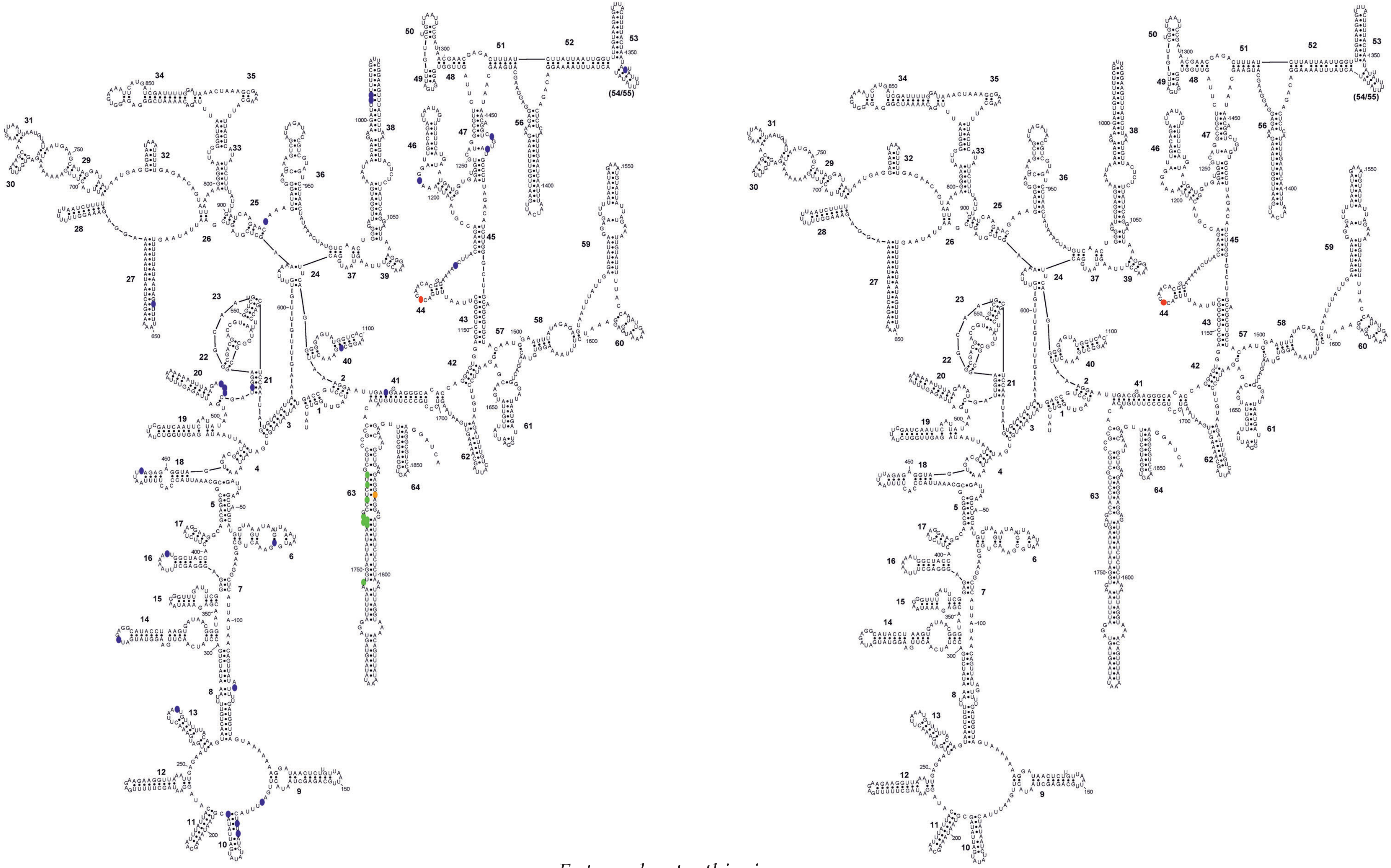

Entamoeba struthionis

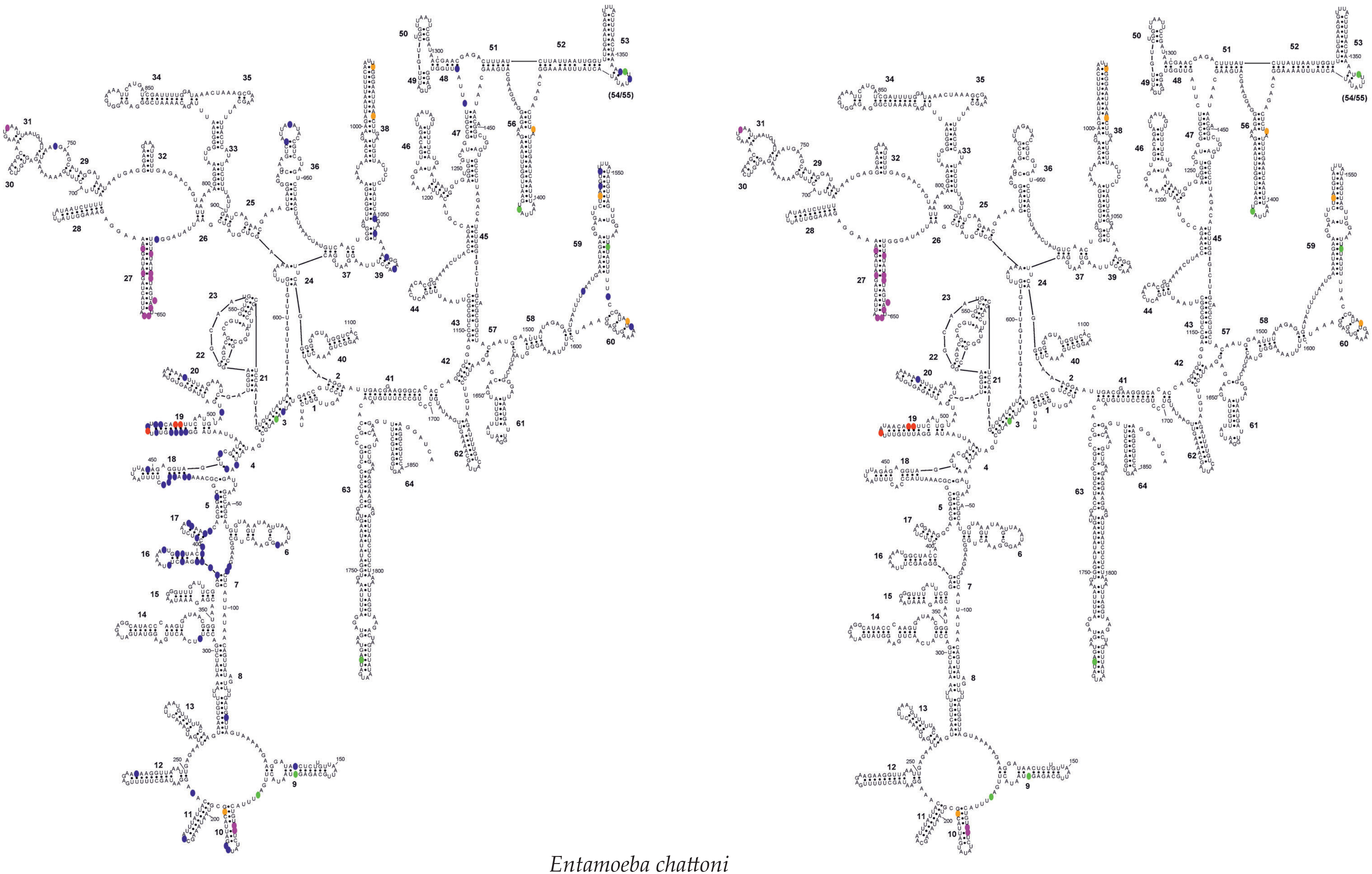

Entamoeba chattoni
