# Supplementary material for: Assessing Species Delimitation in Entamoeba (Amoebozoa: Endamoebidae) Using the Small Subunit rRNA Gene: Its Application to the Entamoeba polecki Complex
Source: Microorganisms. 2026 Feb 3;14(2):360. doi: 10.3390/microorganisms14020360 (PMC12942770; doi:10.3390/microorganisms14020360)
Supplement: Supplementary file 1 [file microorganisms-14-00360-s001.zip › Supplementary File 6.pdf]

Supplementary File 6. Comparisons of the secondary structures of helices 16 to 19 of the SSU rRNA molecule in *Entamoeba chattoni* (sequences AF149912 and PP064054).

Diagrams show the partial SSU-rRNA secondary structure of the reference *E. chattoni* sequence (AF149912) and of sequence PP064054, highlighting positions at which differences between the two sequences occur. Differences in the primary sequence affecting helix base pairs are indicated by blue boxes. Nucleotides involved in a compensatory base change in helix 19 are highlighted by grey circles. Positions are given according to sequence AF149912.

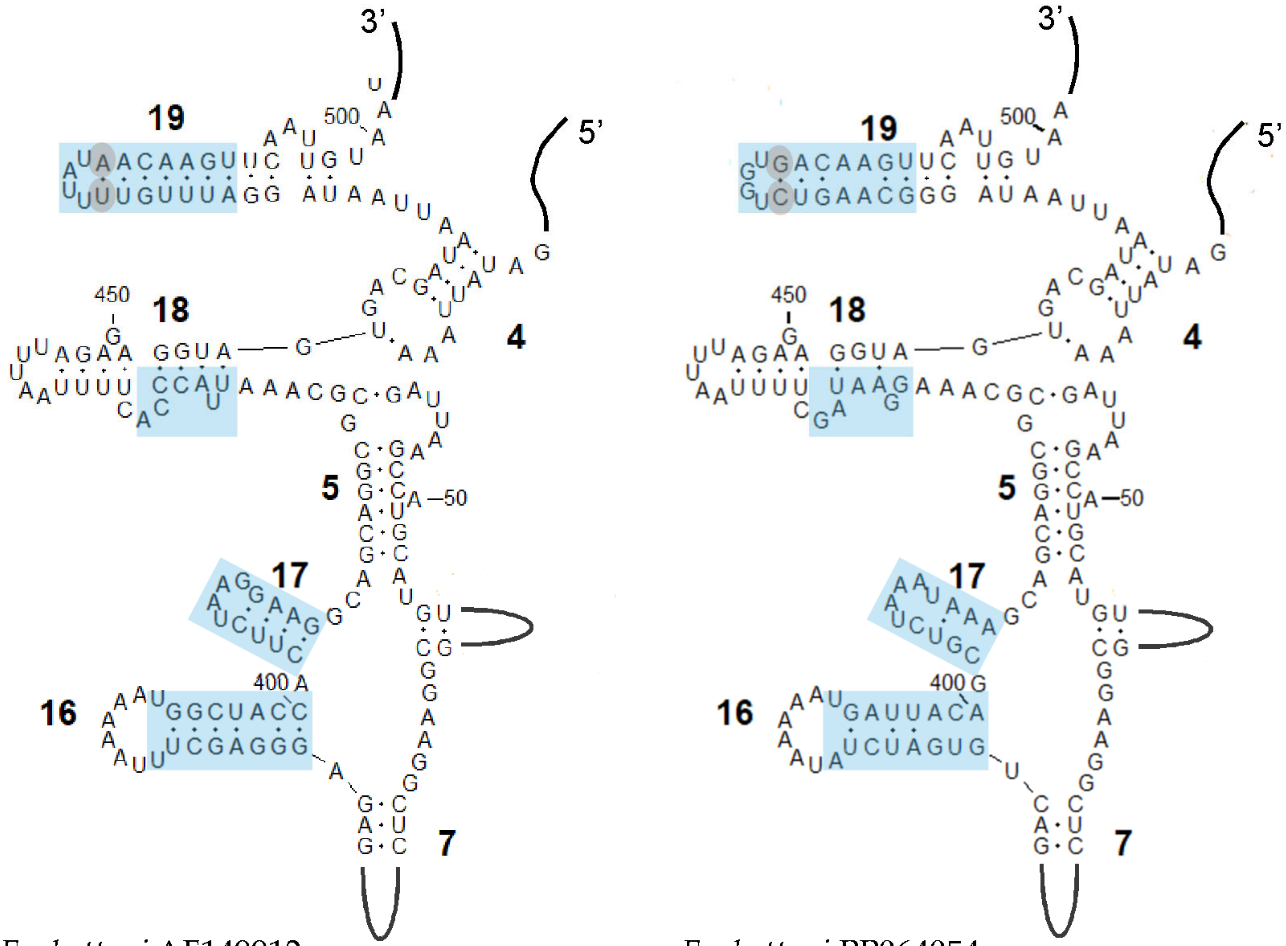

E. chattoni AF149912

E. chattoni PP064054
